# Supplementary material for: vissE: a versatile tool to identify and visualise higher-order molecular phenotypes from functional enrichment analysis
Source: BMC Bioinformatics. 2024 Feb 8;25:64. doi: 10.1186/s12859-024-05676-y (PMC10854147; doi:10.1186/s12859-024-05676-y)
Supplement: Supplementary file 1 — Additional file 1: Additional figures to support the findings reported in this work [file 12859_2024_5676_MOESM1_ESM.pdf]

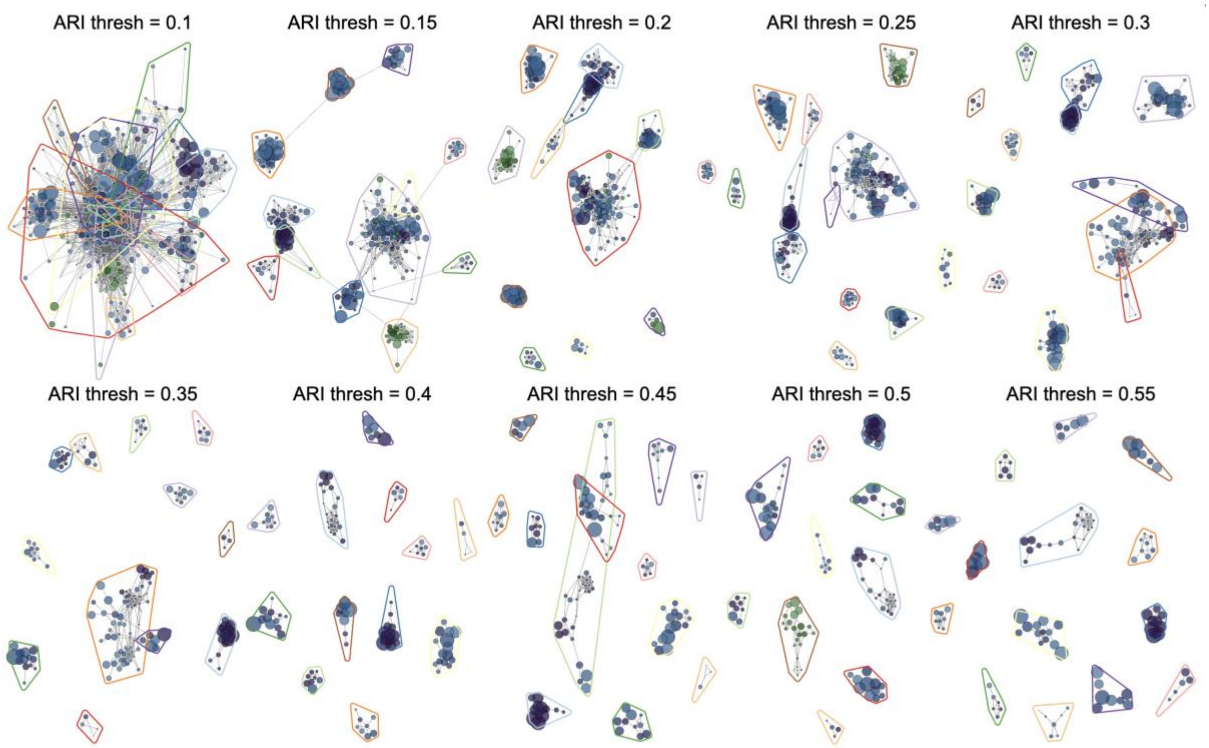

Supplementary Figure 1: Increasing the Adjusted Rand Index (ARI) threshold results in sparser networks therefore smaller more consistent clusters. Ideal thresholds lie within the range of 0.2 and 0.4 as these ensure clusters are large enough to condense information while being consistent in the biology they represent.

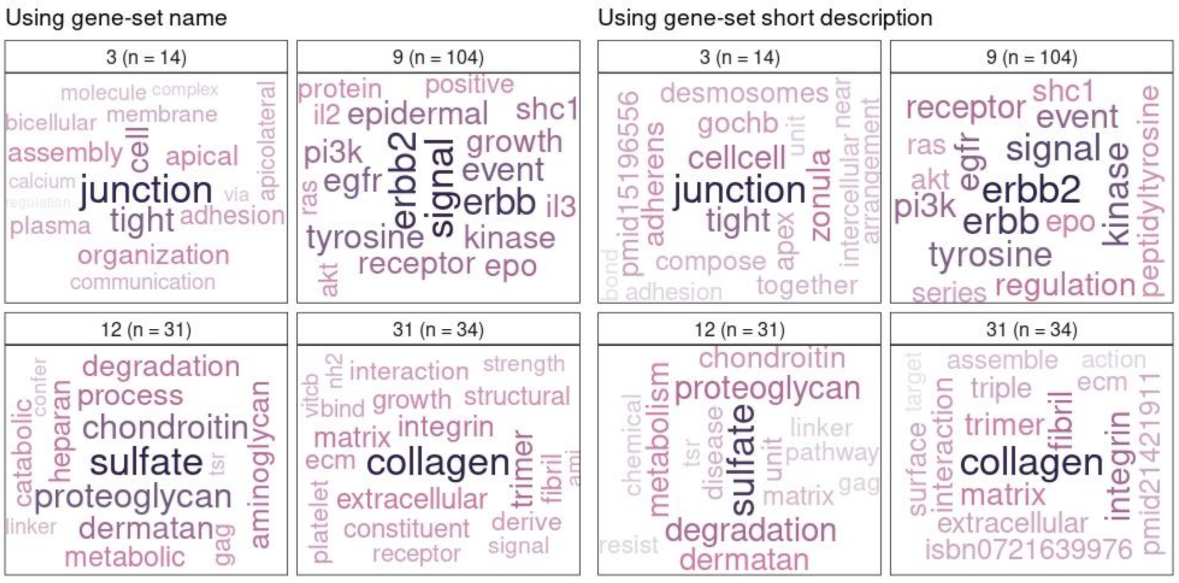

Supplementary Figure 2: The same biological themes are inferred whether using gene-set names or their short descriptions to annotate gene-set clusters identified using vissE.

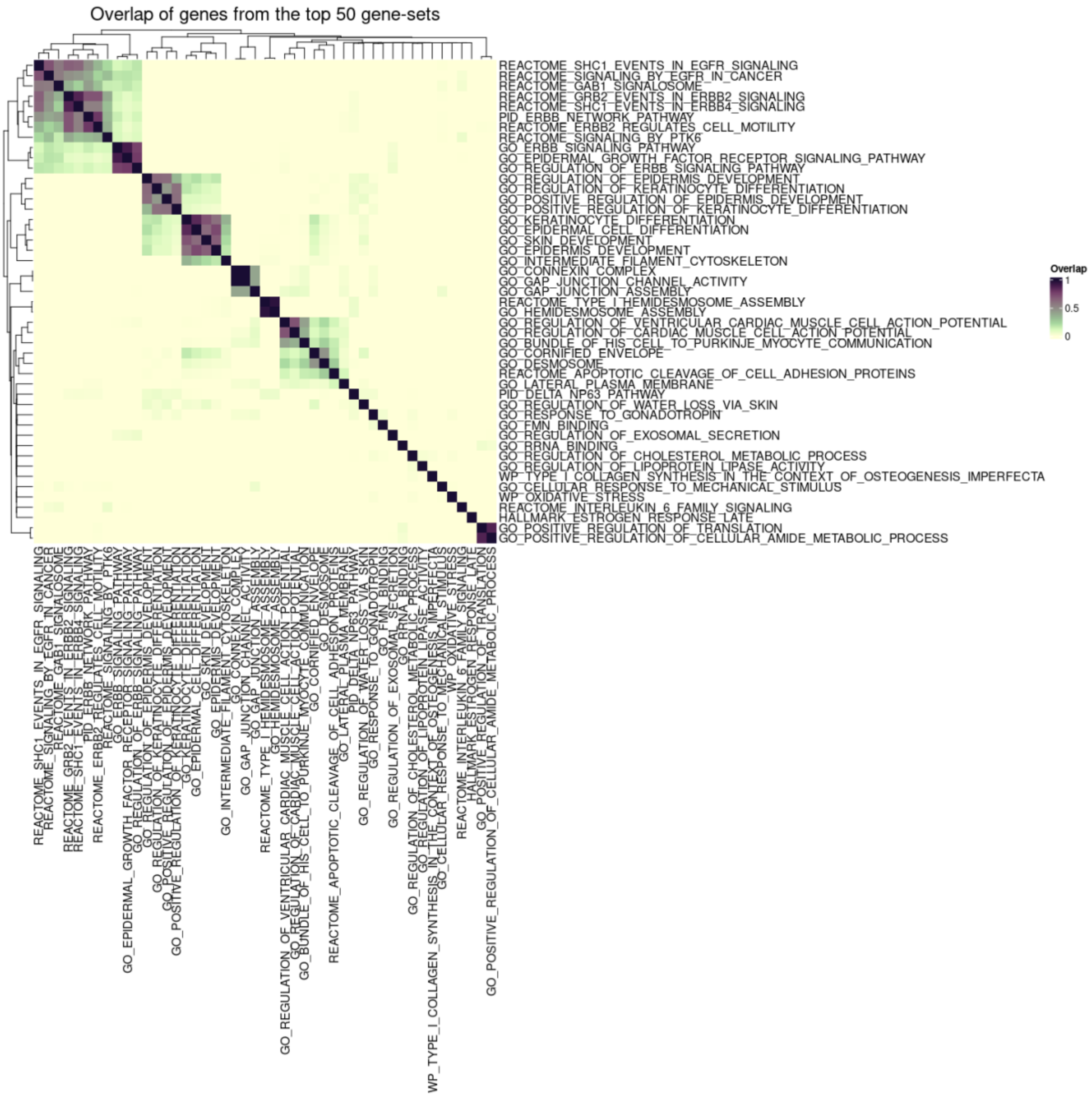

Supplementary Figure 3: The top 50 significant gene-sets in the HMLE system differential expression analysis shared a large number of differentially expressed genes and thus were representing redundant functional information.

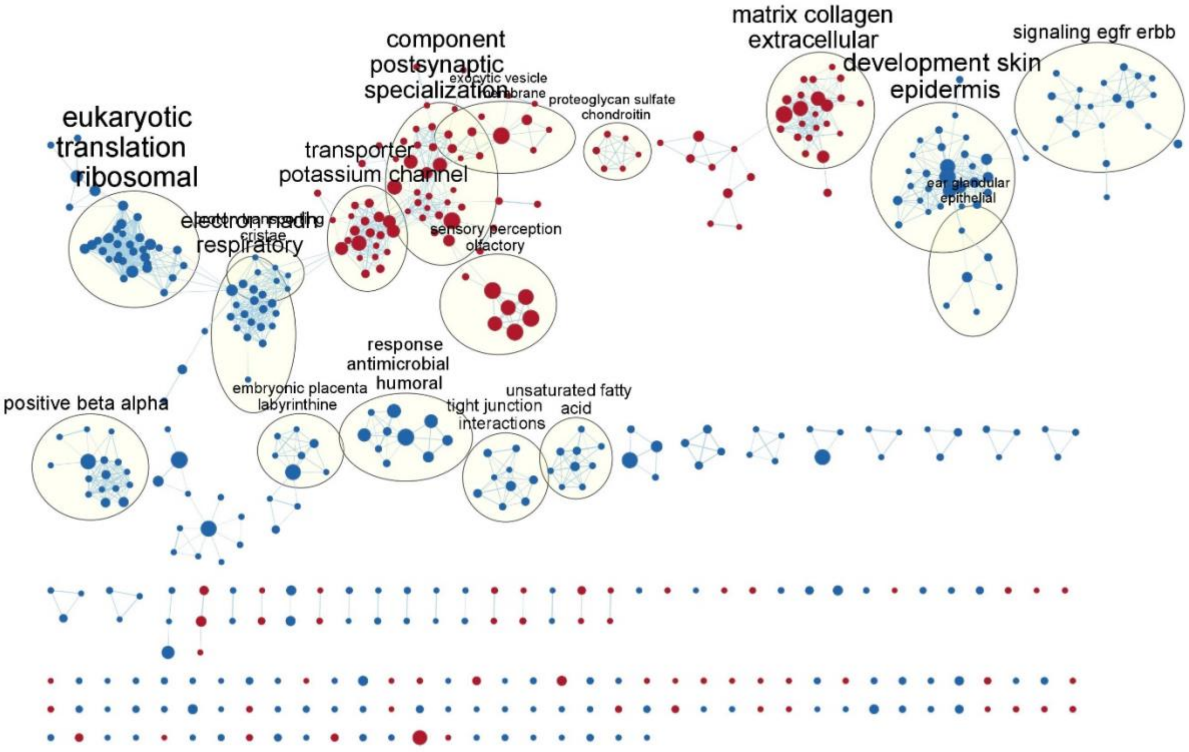

Supplementary Figure 4: Gene-set clusters and their annotations derived from EnrichmentMap to characterise the epithelial to mesenchymal transition in the HMLE system. Each node represents a gene-set and edges connect similar gene-sets. Nodes are coloured based on the direction of change: red – upregulated in mesenchymal cells; blue – upregulated in epithelial cells.

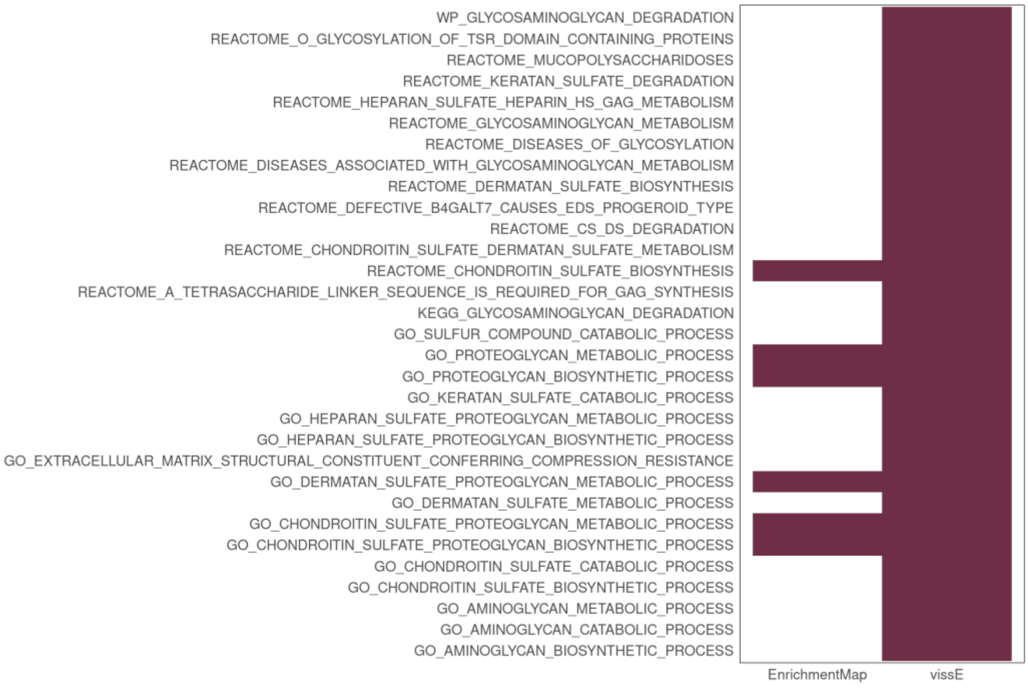

Supplementary Figure 5: Gene-sets identified by the EnrichmentMap pipeline (6) and the vissE pipeline (31) belonging to (shaded) the proteoglycan metabolism biological theme identified by both pipelines.

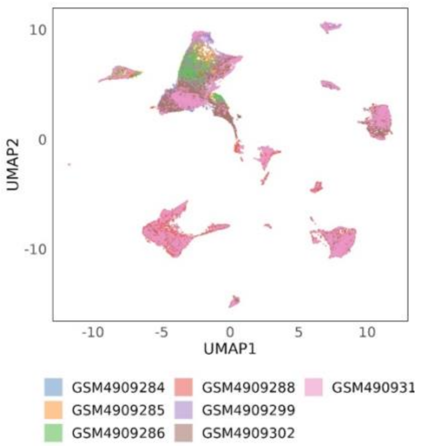

Supplementary Figure 6: UMAP of cells from seven breast cancer patients representing two breast cancer subtypes. Cells are coloured based on the patient they were derived from.

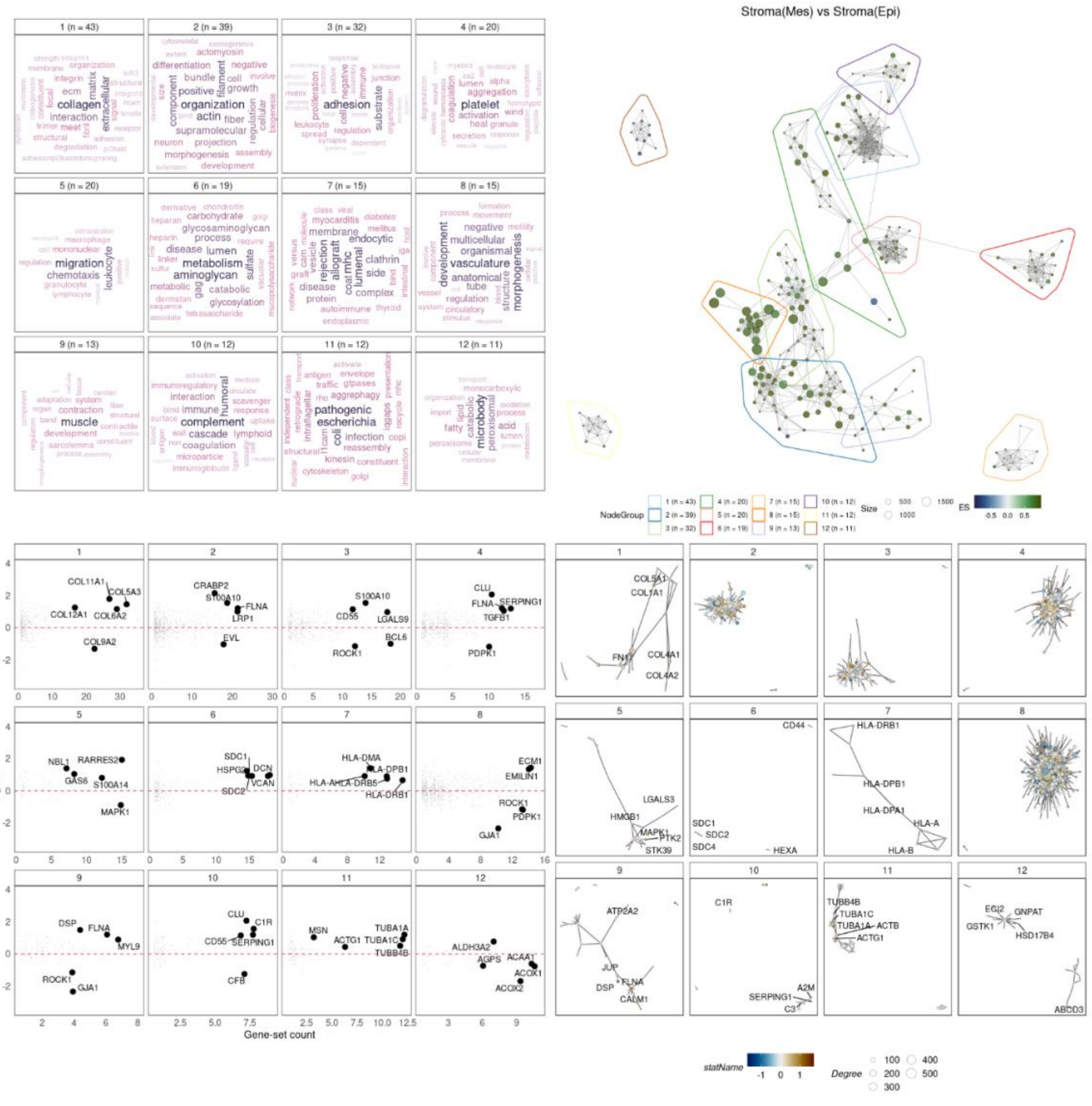

Supplementary Figure 7: The top 12 themes identified from a vissE analysis of the results of a differential expression analysis between stromal spots surrounded by malignant epithelial cells vs. stromal spots surrounded by malignant mesenchymal cells.
