# Supplementary figures and images for: vissE: a versatile tool to identify and visualise higher-order molecular phenotypes from functional enrichment analysis
Source: BMC Bioinformatics. 2024 Feb 8;25:64. doi: 10.1186/s12859-024-05676-y (PMC10854147; doi:10.1186/s12859-024-05676-y)

a)

mesHMLE vs HMLE

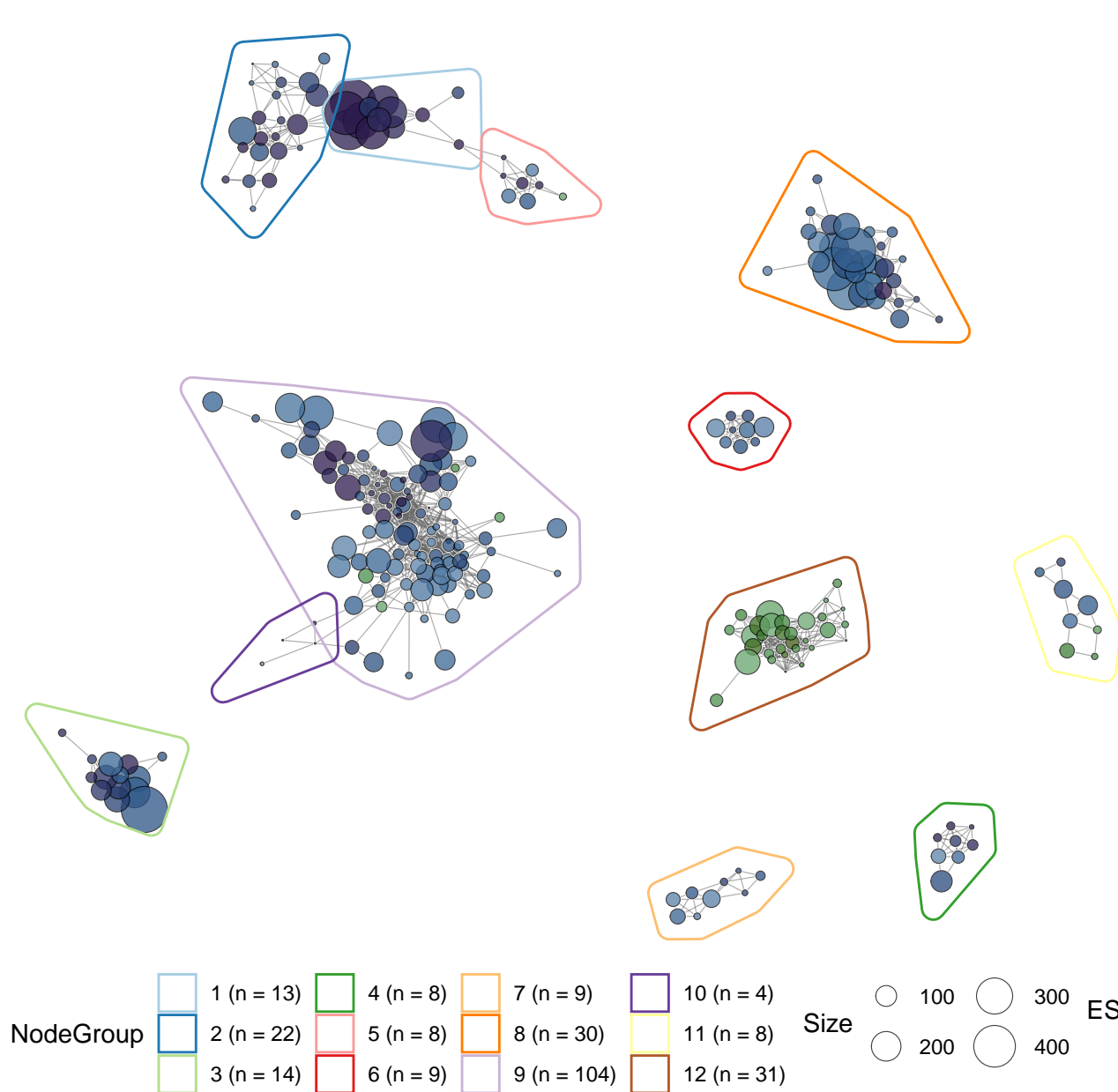

b)

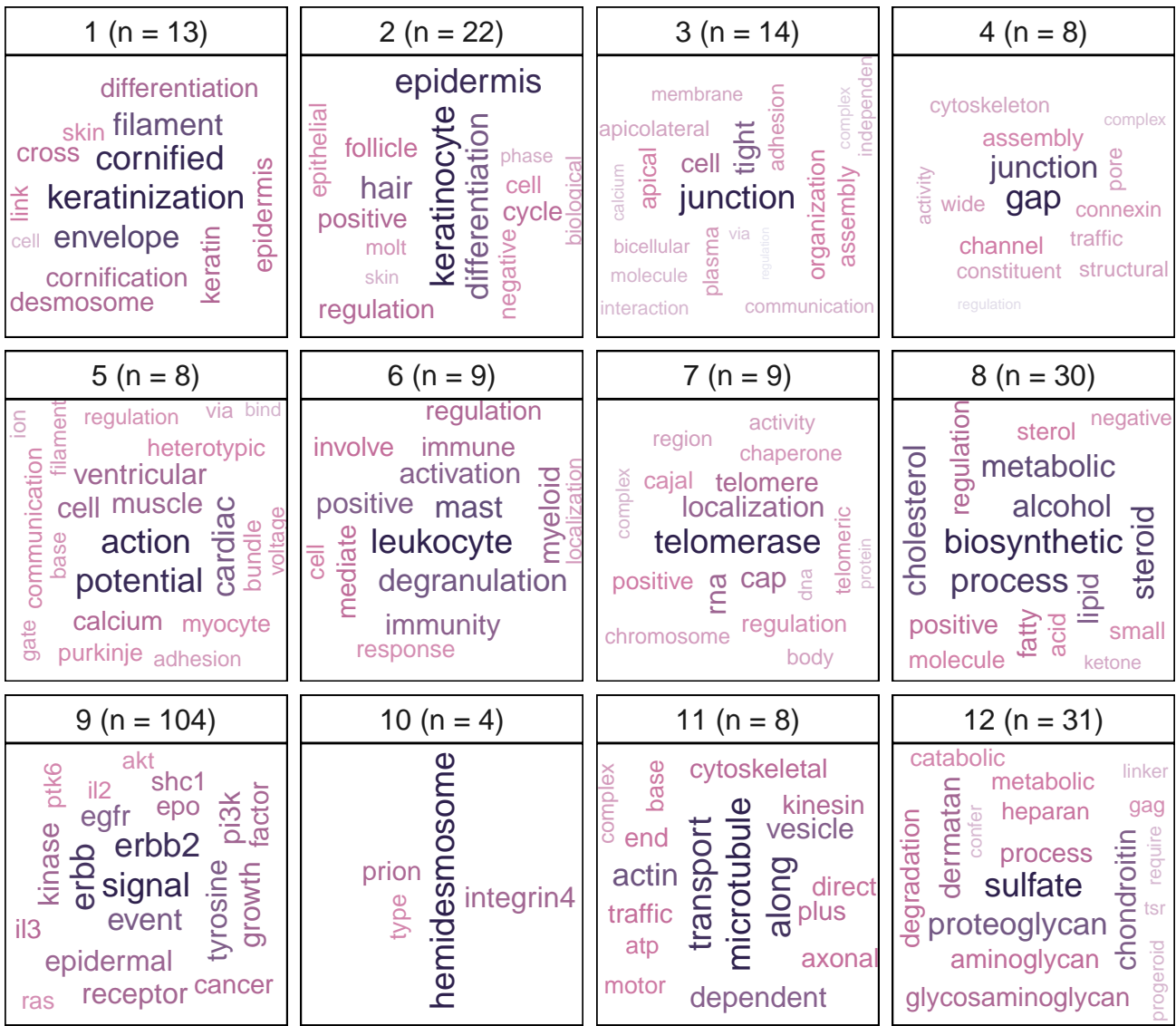

c)

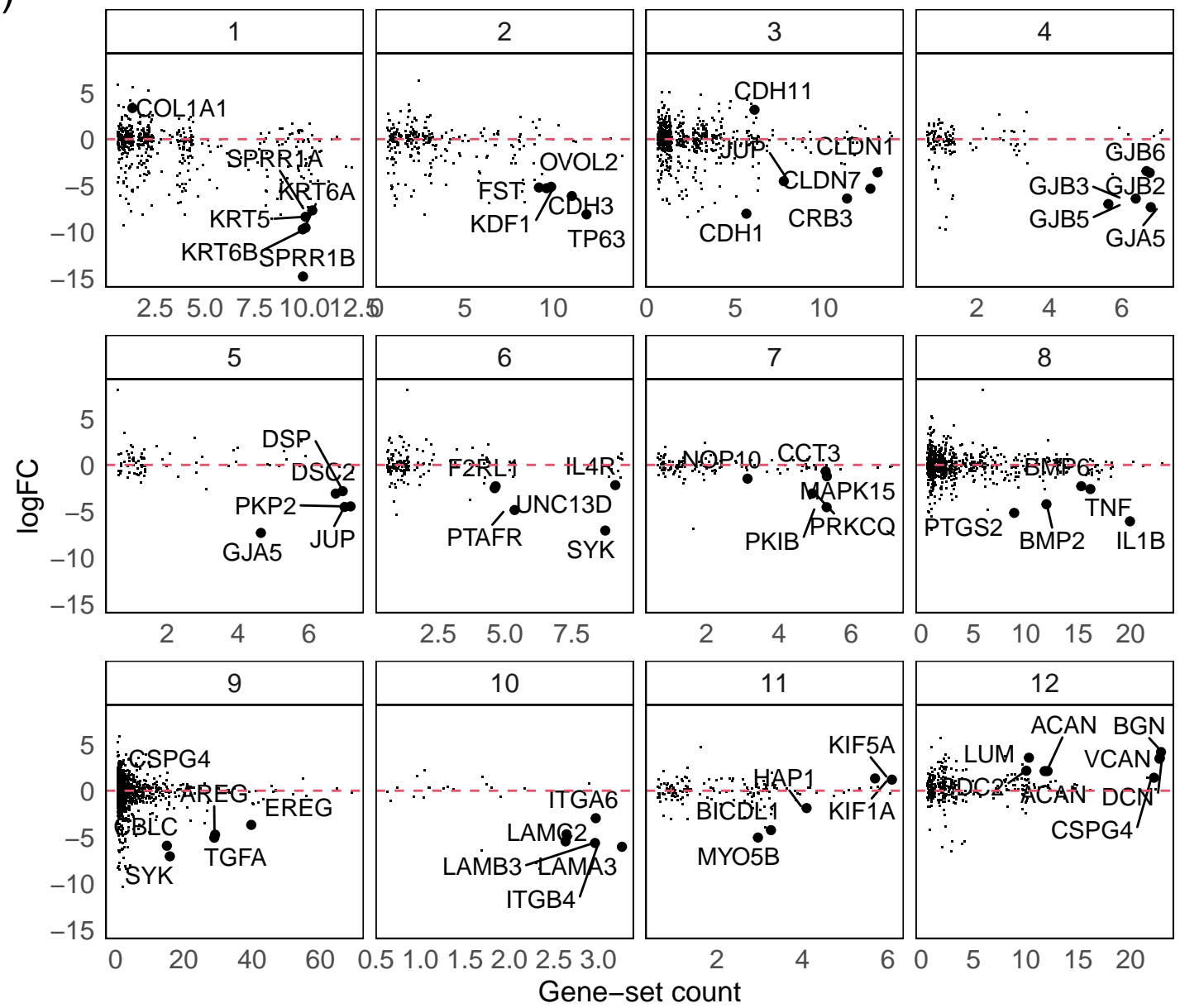

d)

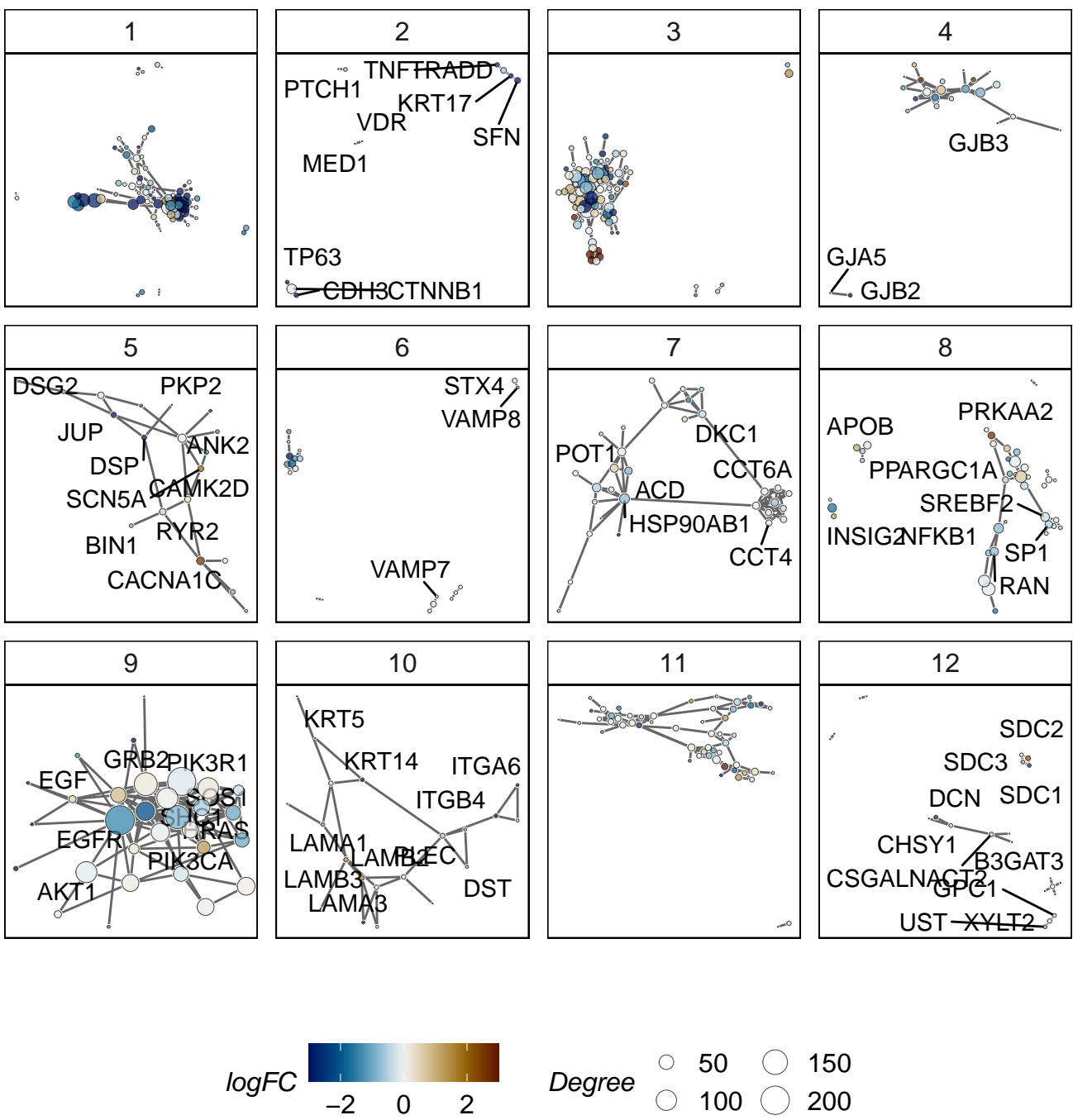

a)

mesHMLE vs HMLE

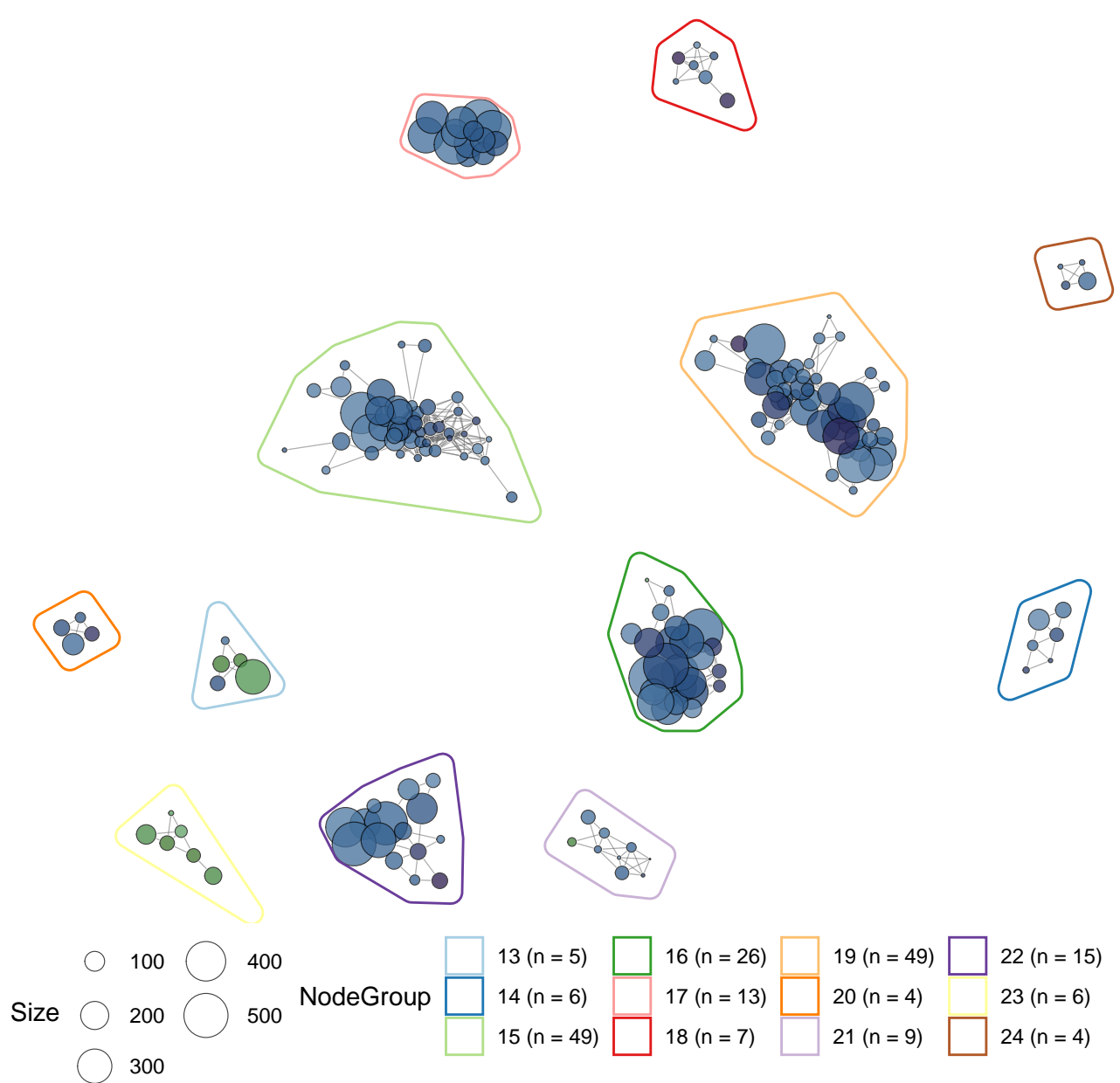

b)

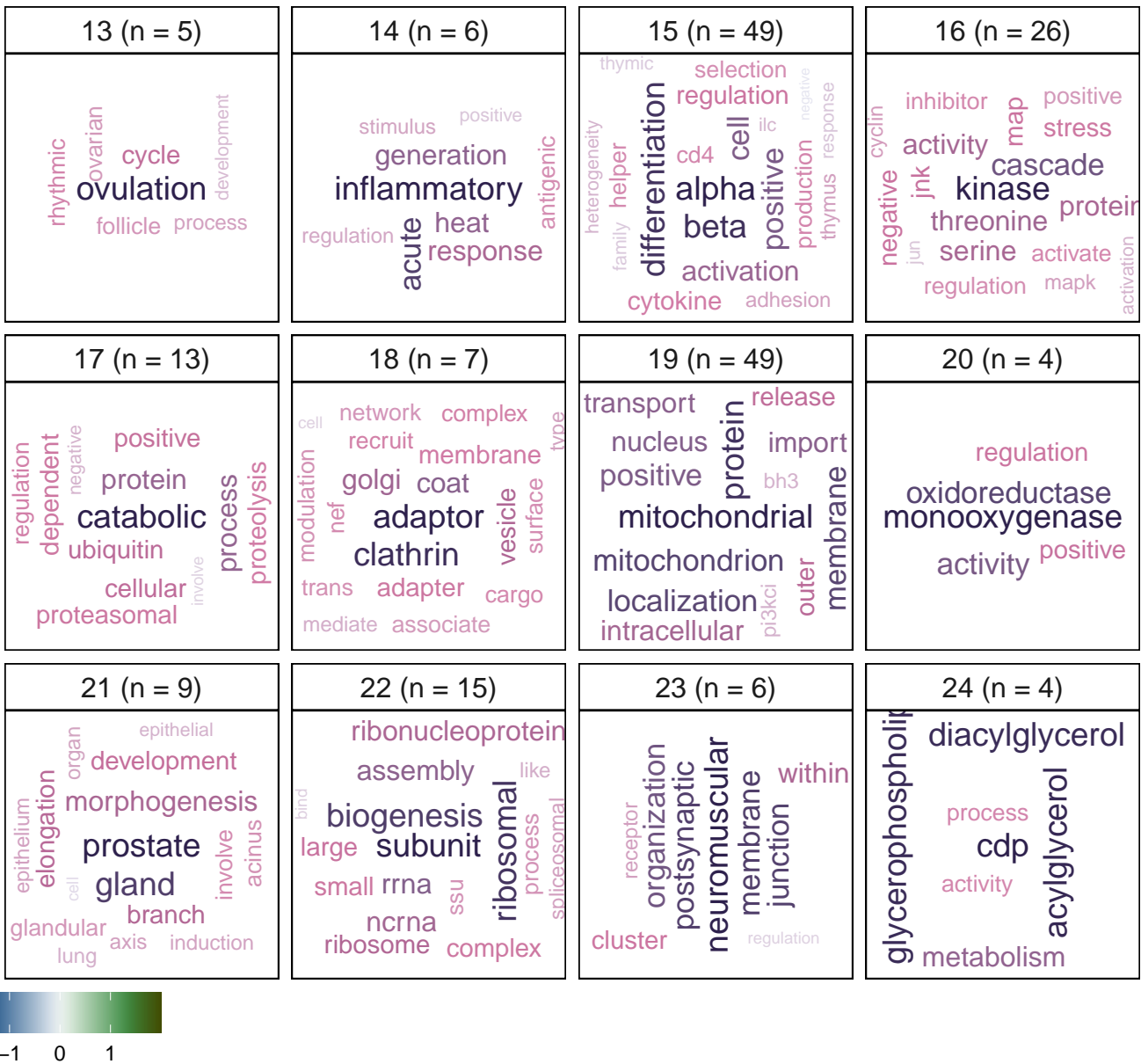

c)

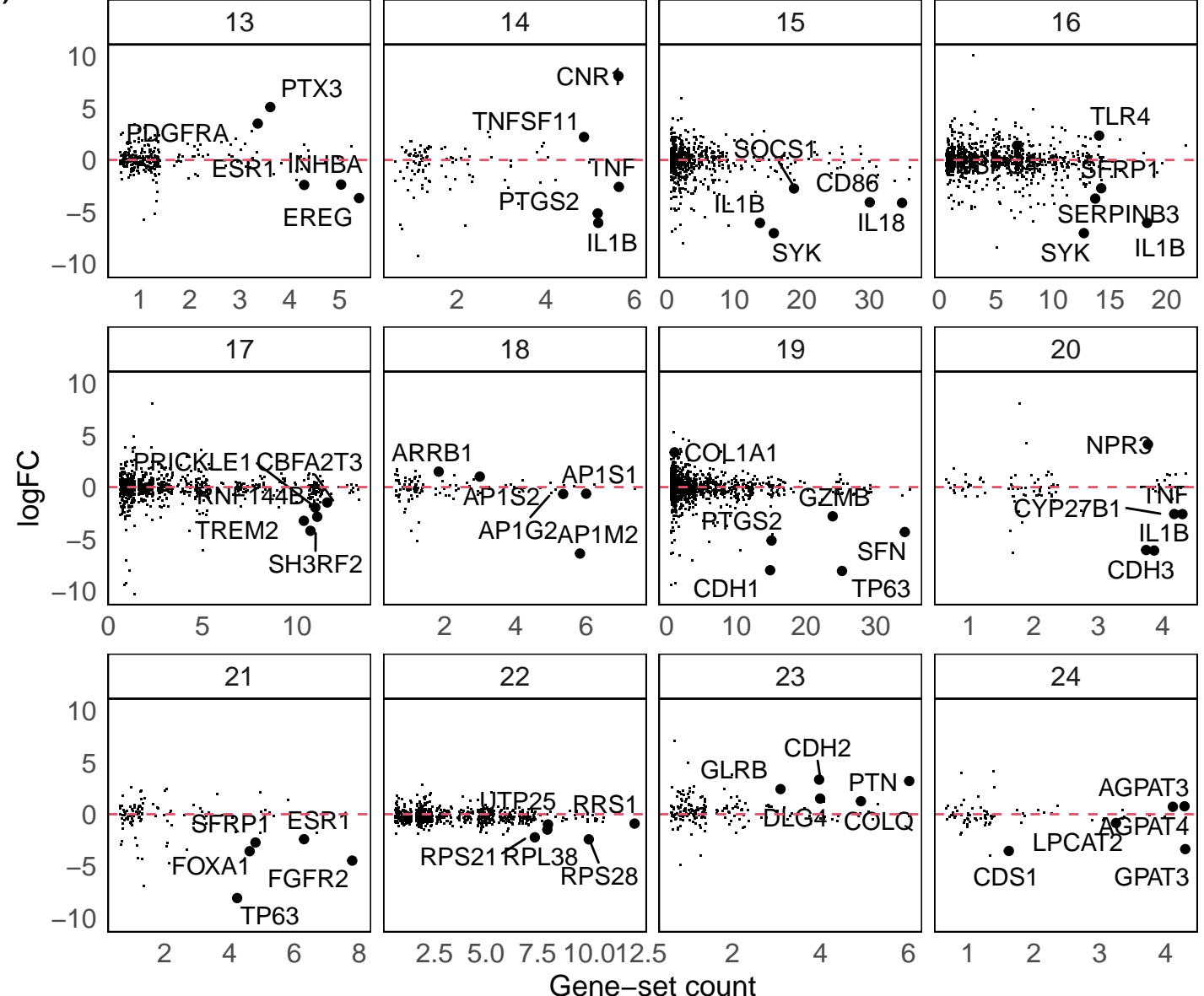

d)

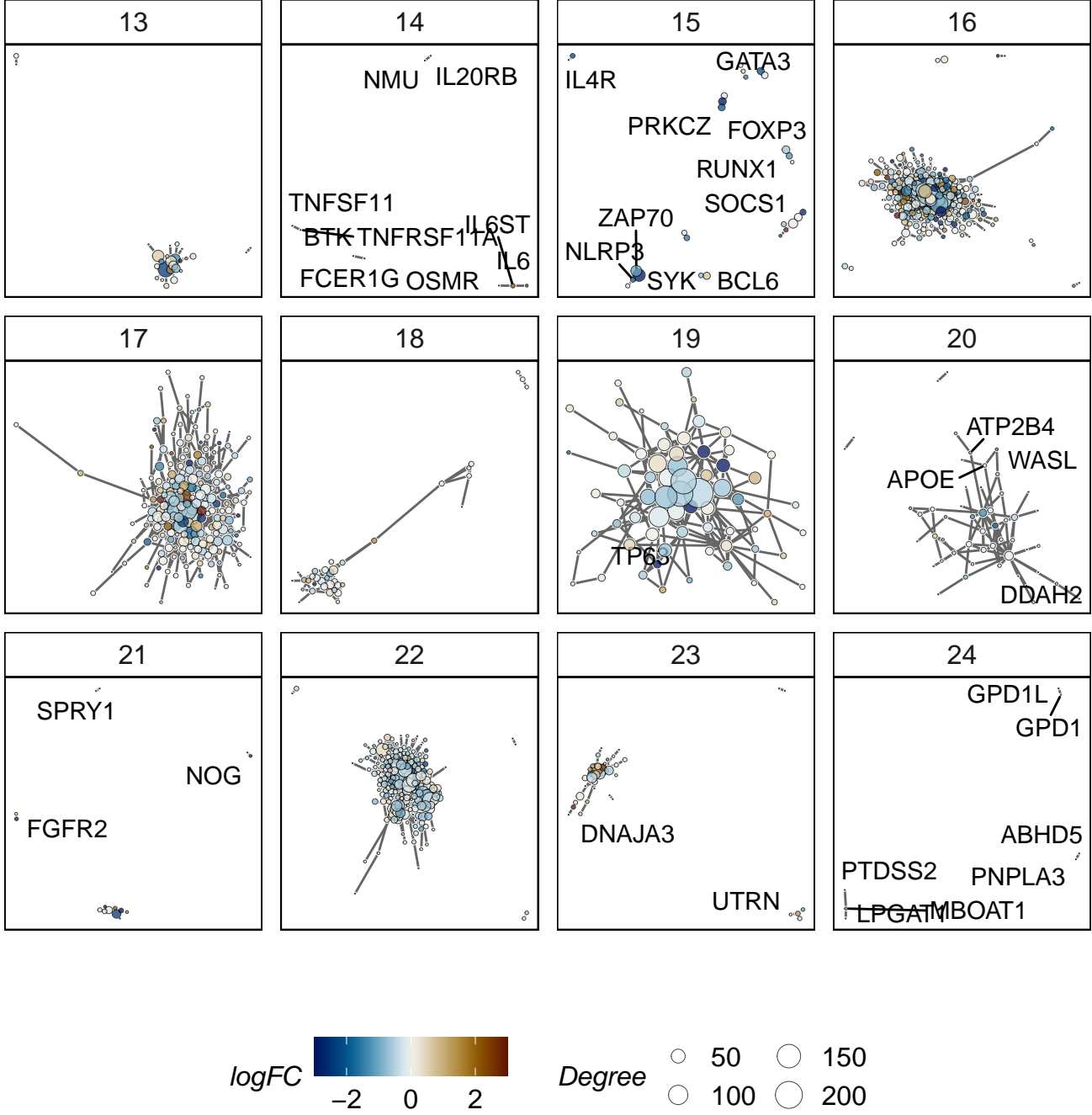

a)

mesHMLE vs HMLE

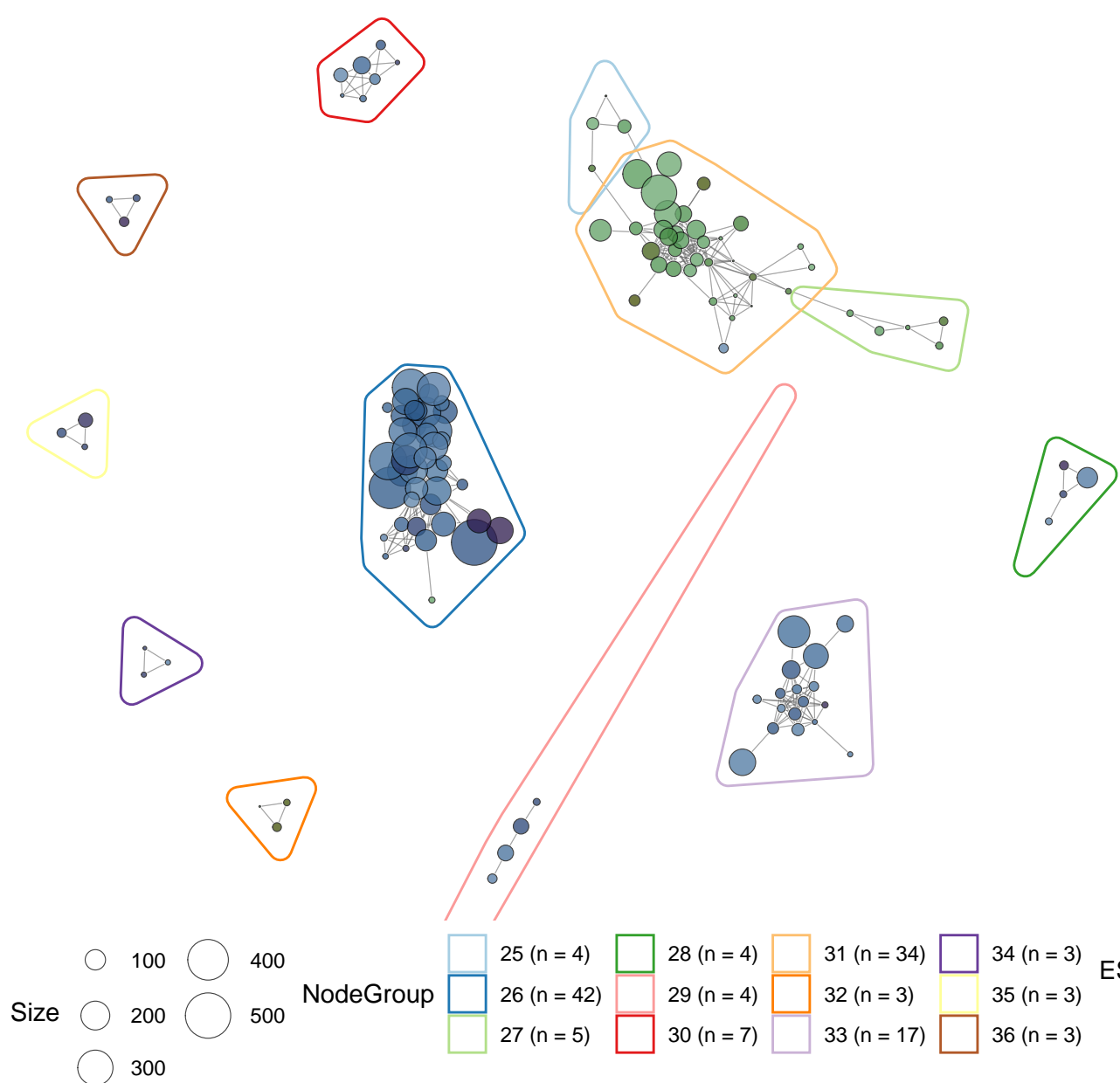

b)

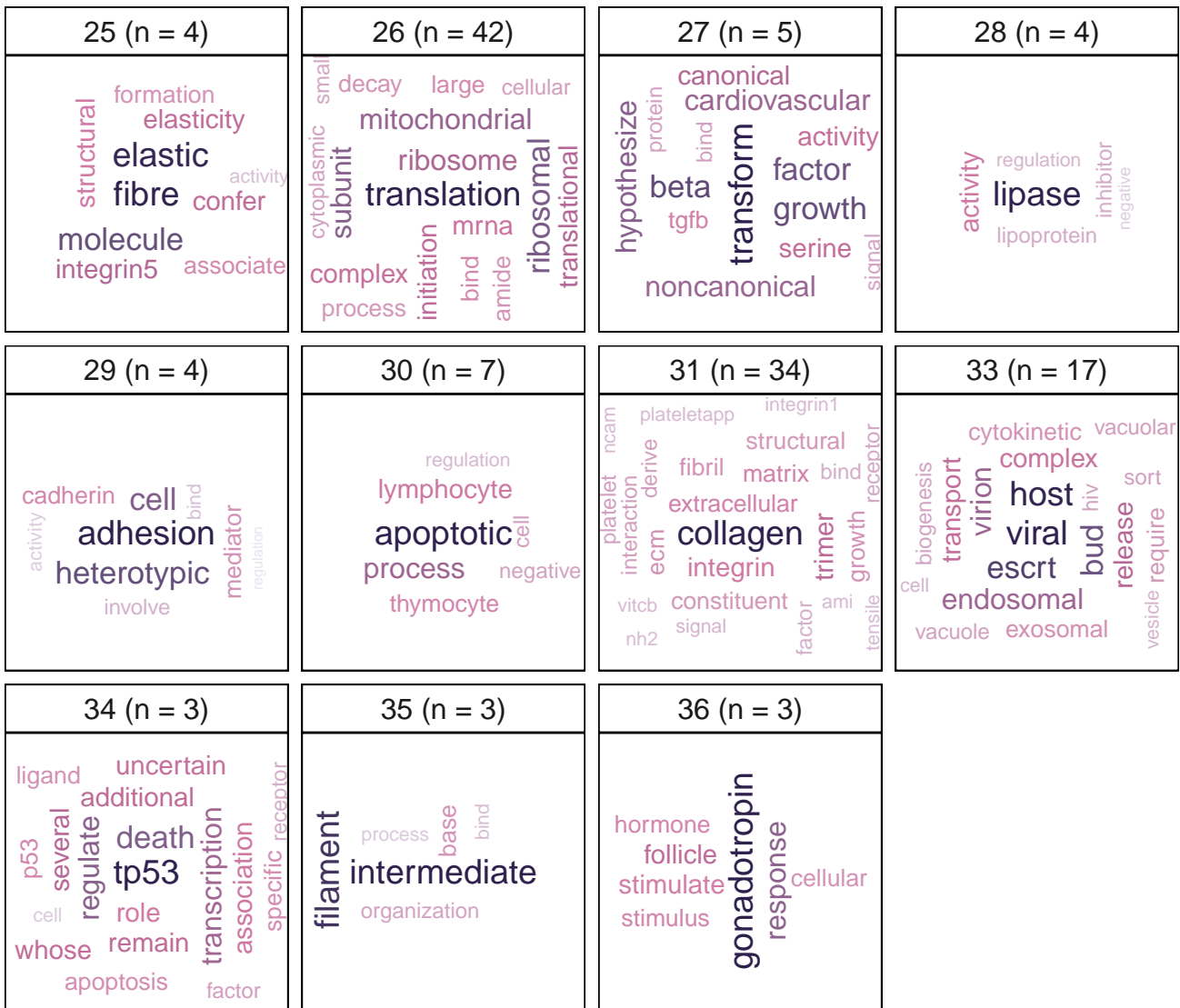

c)

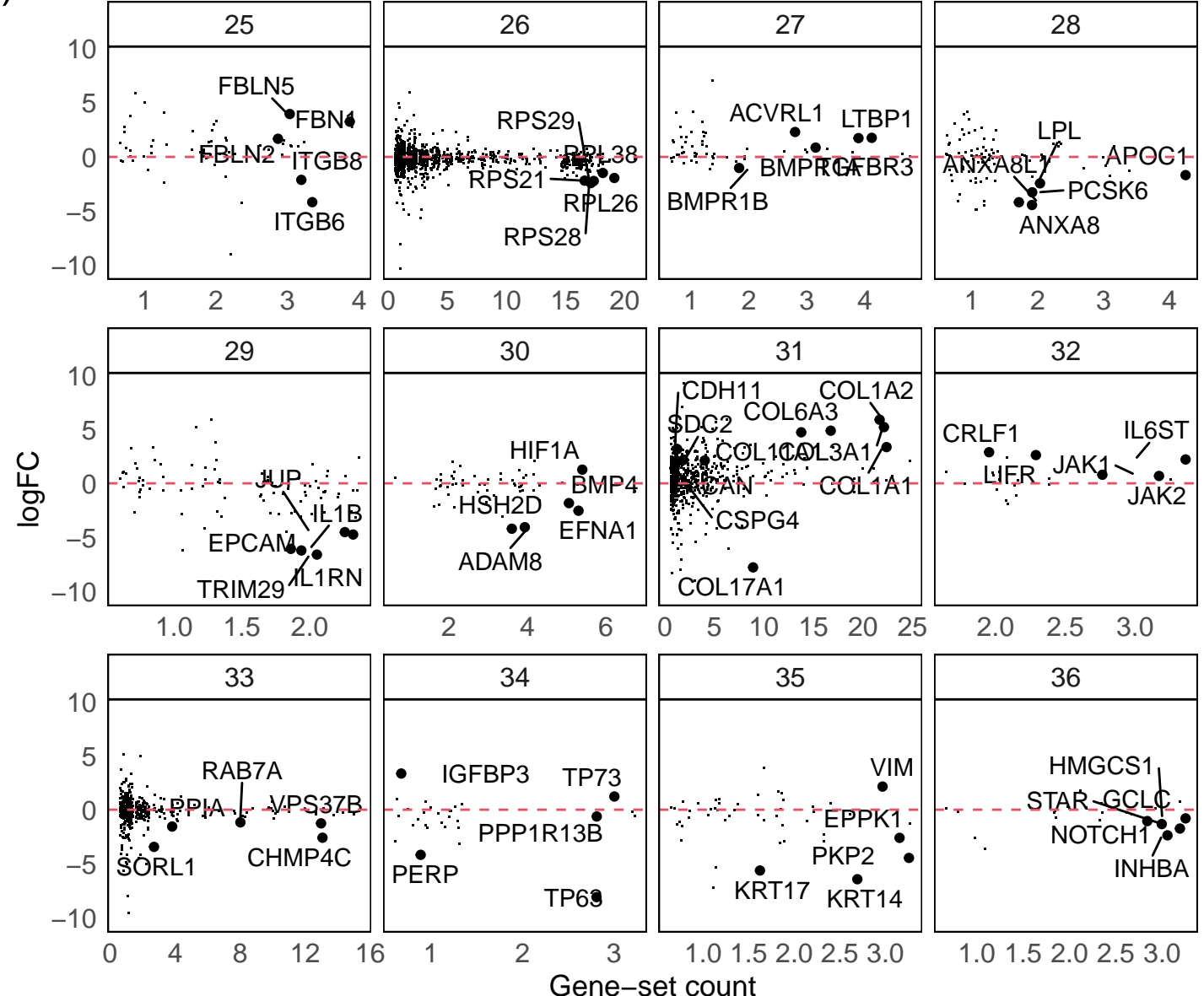

d)

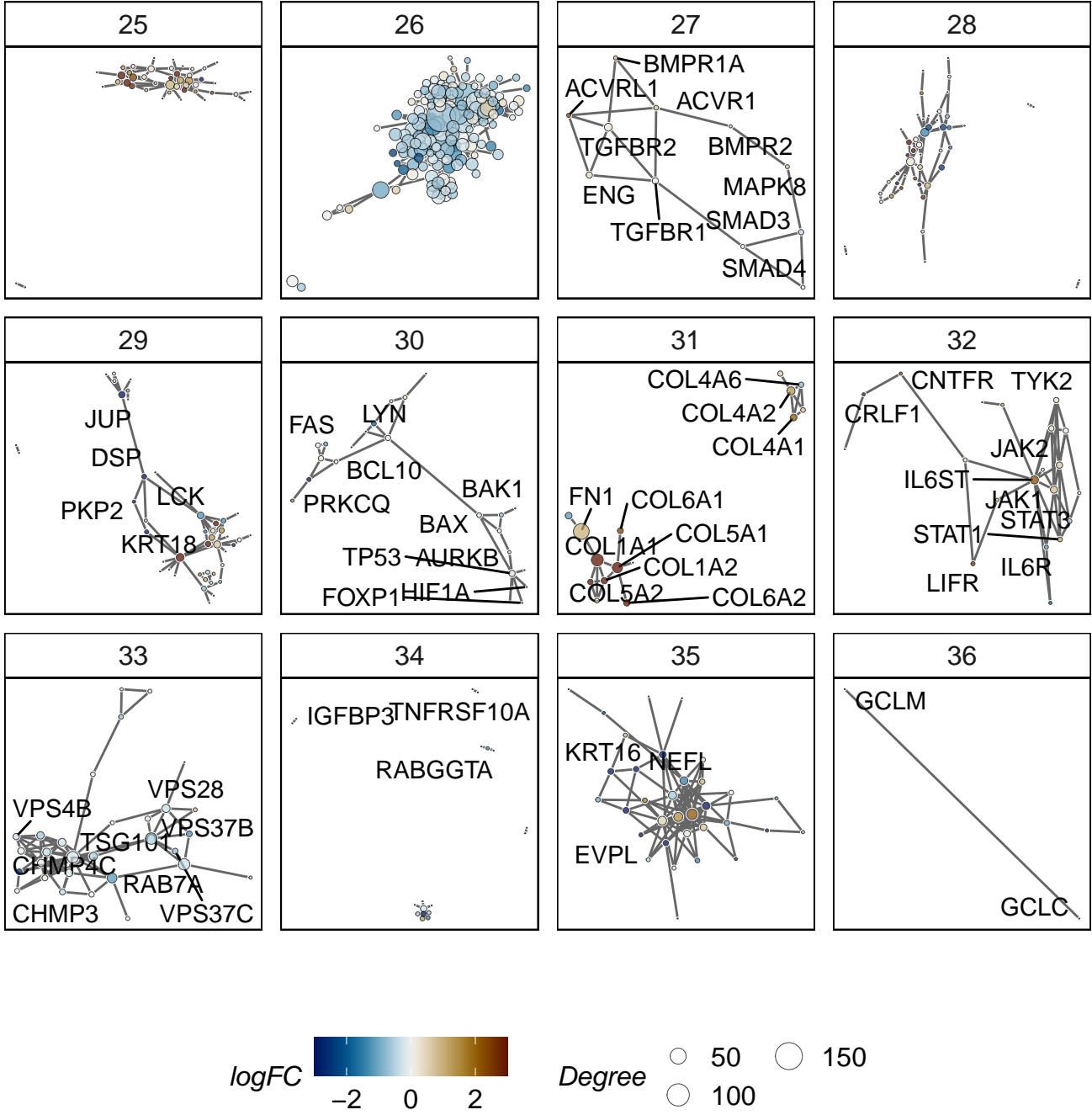

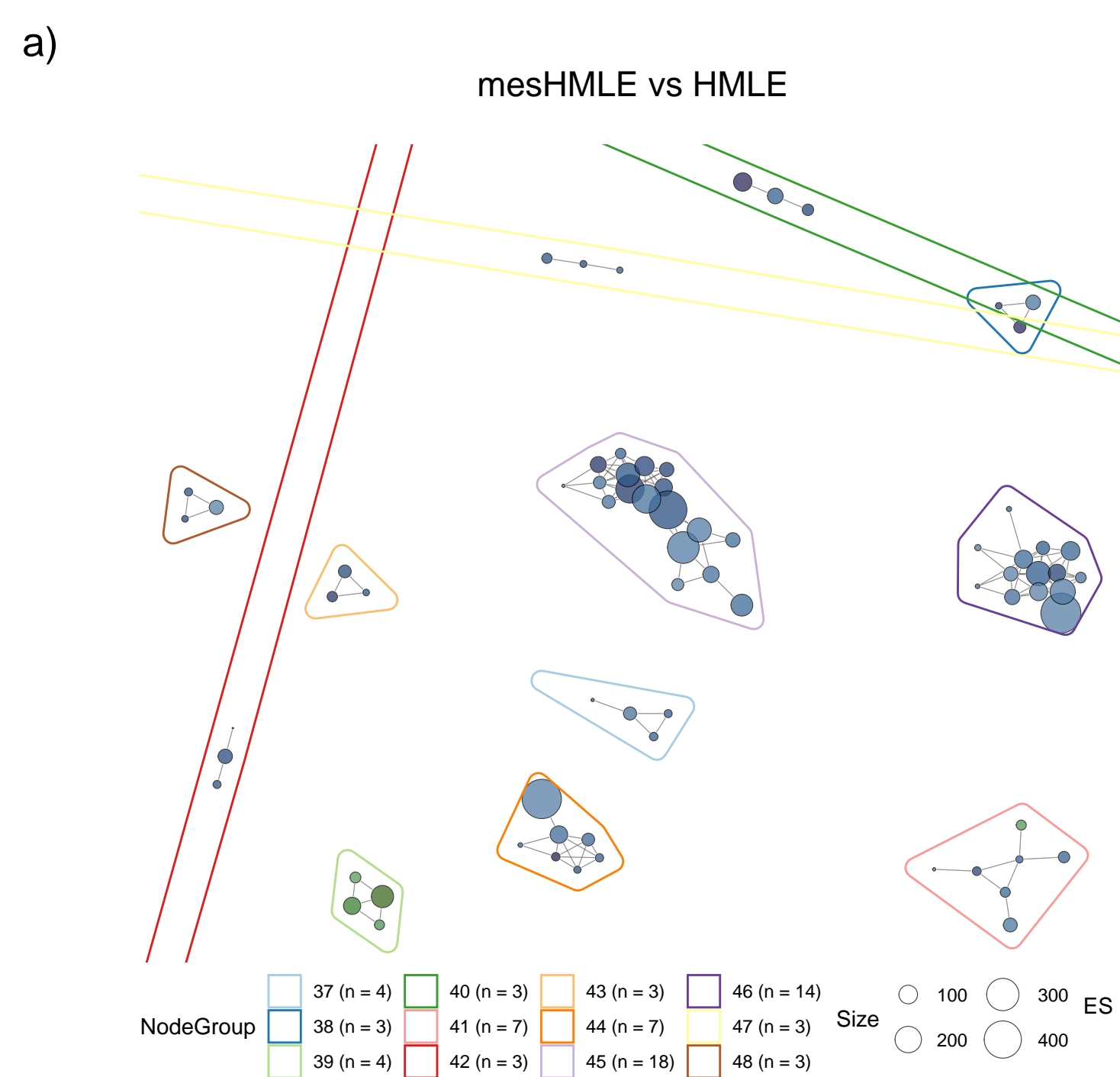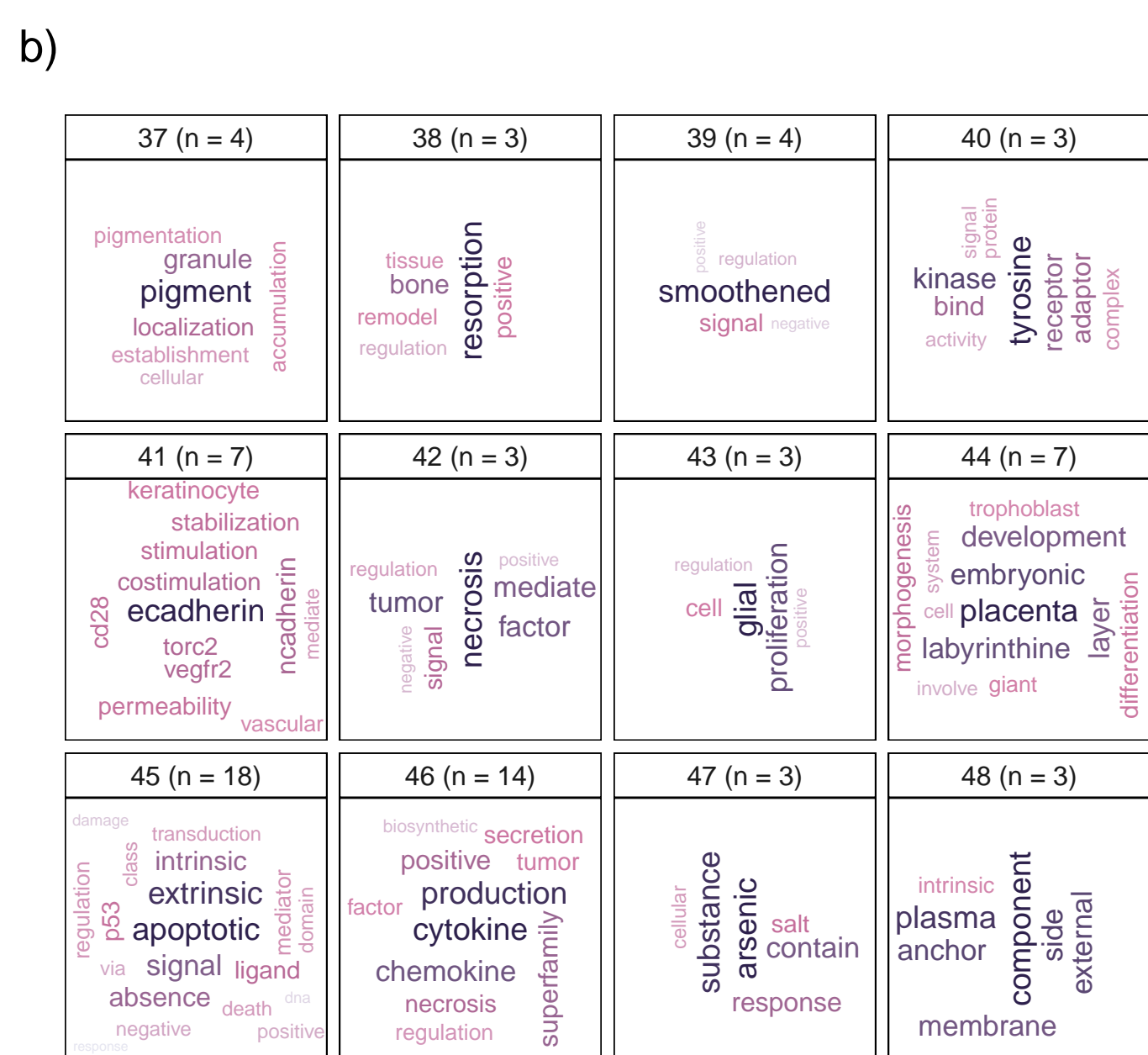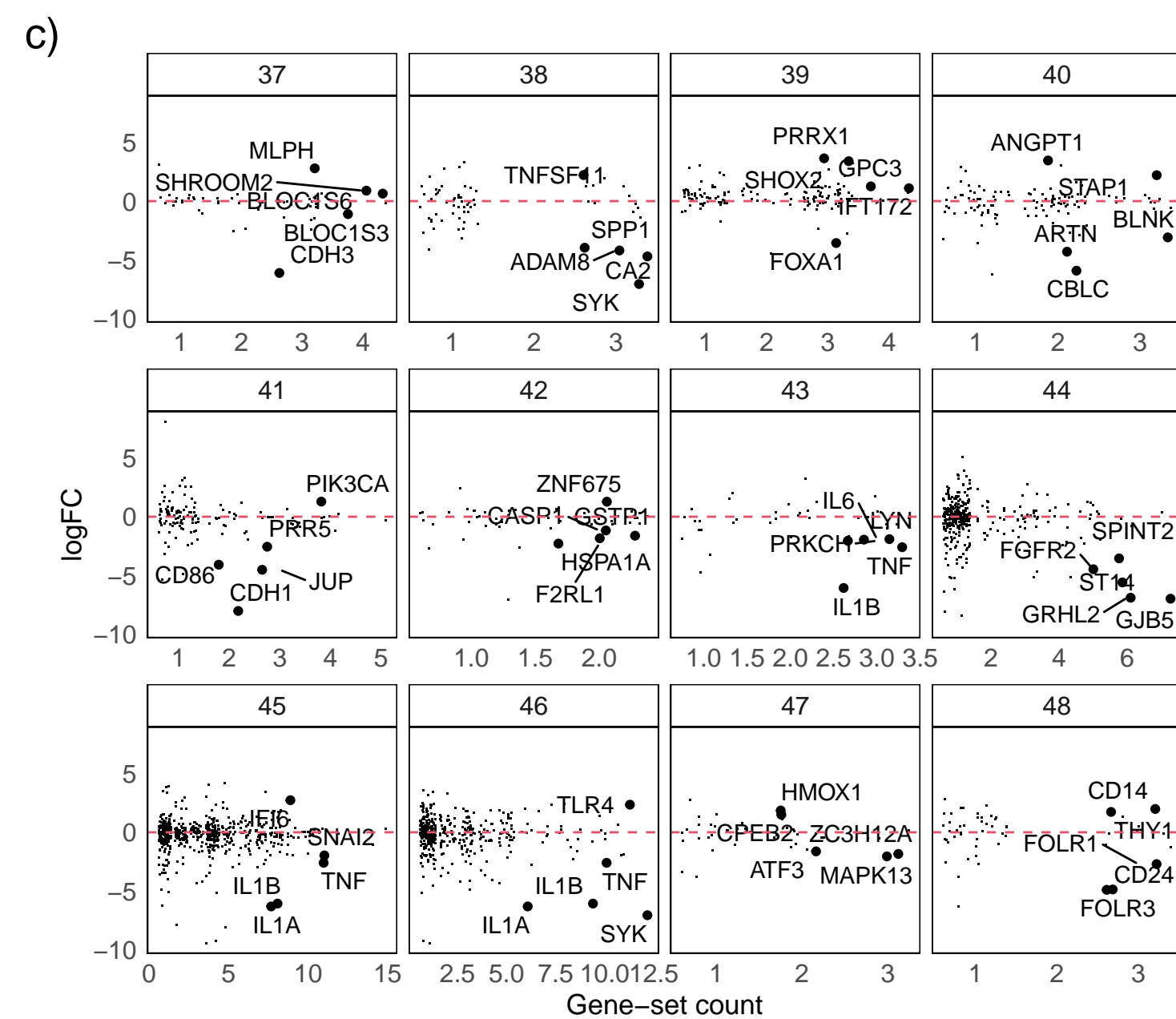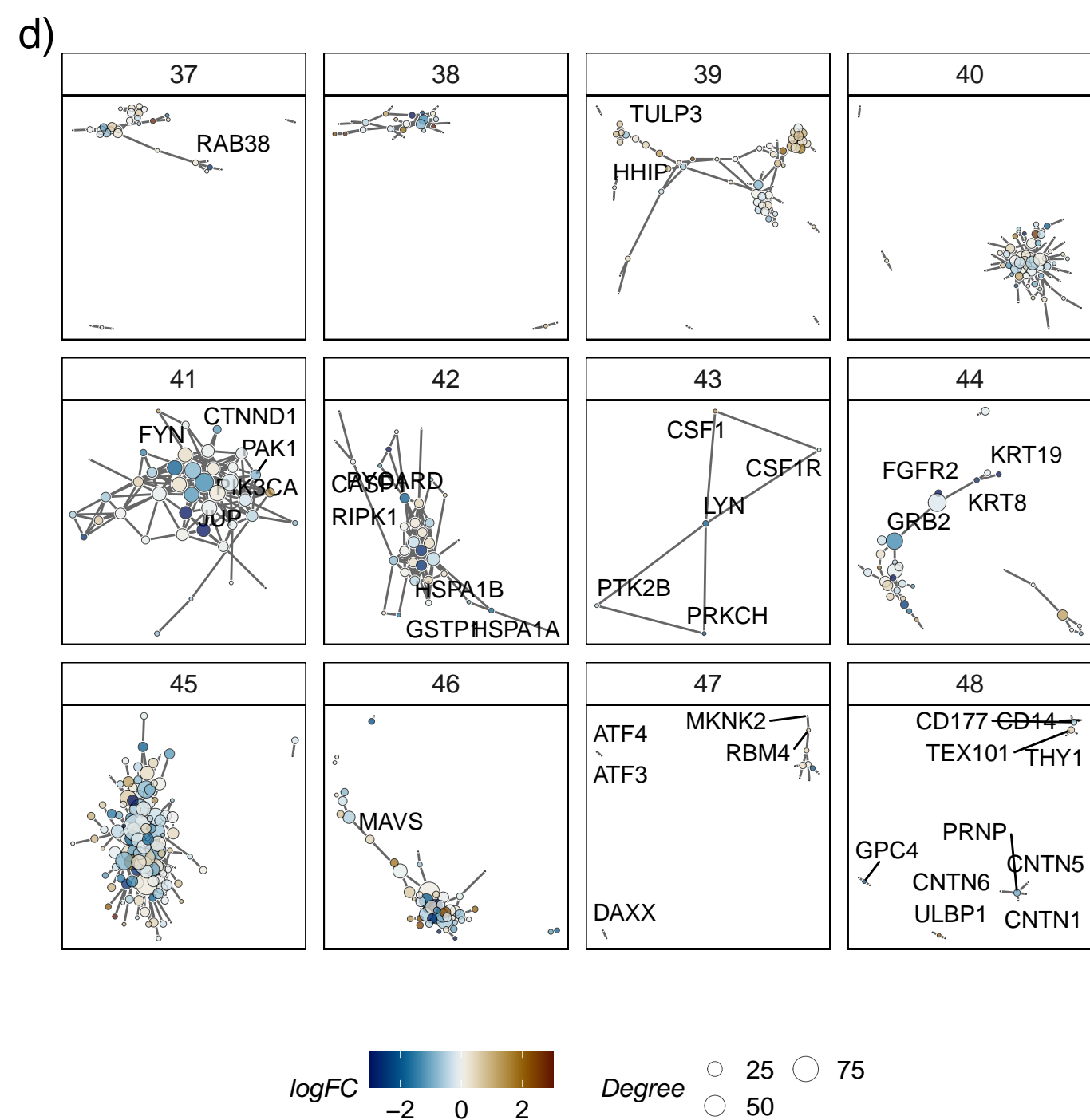

a)

mesHMLE vs HMLE

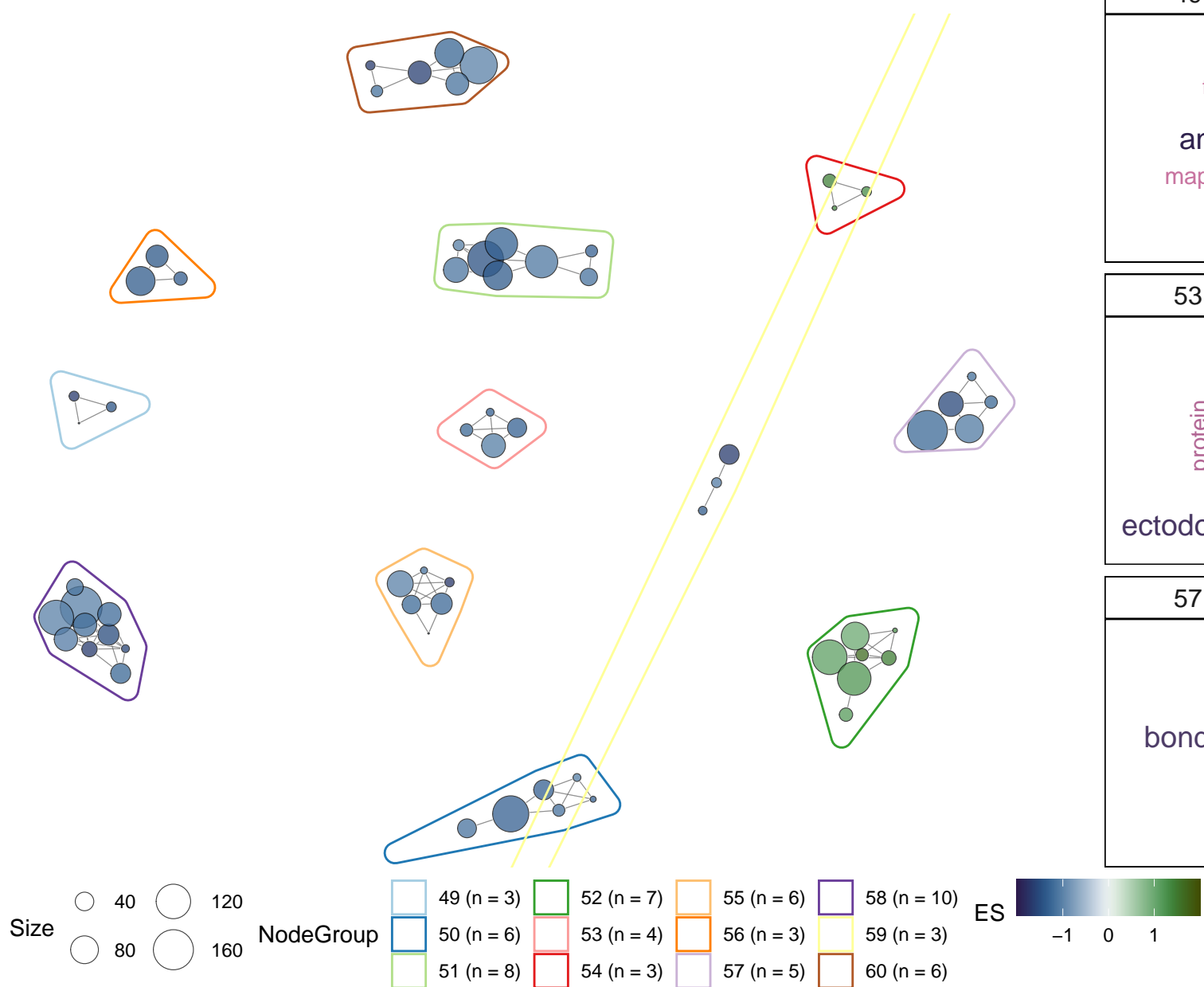

b)

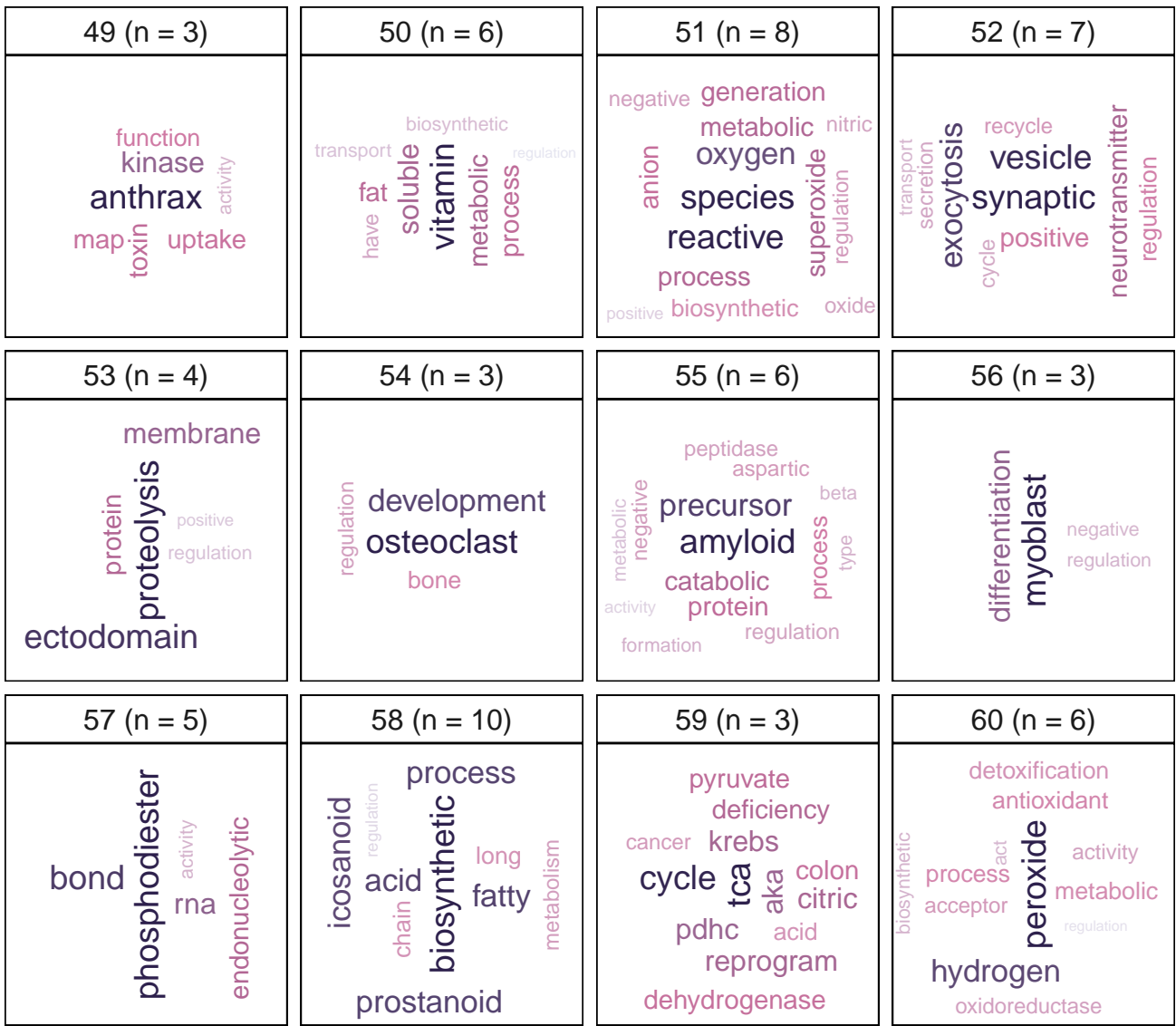

c)

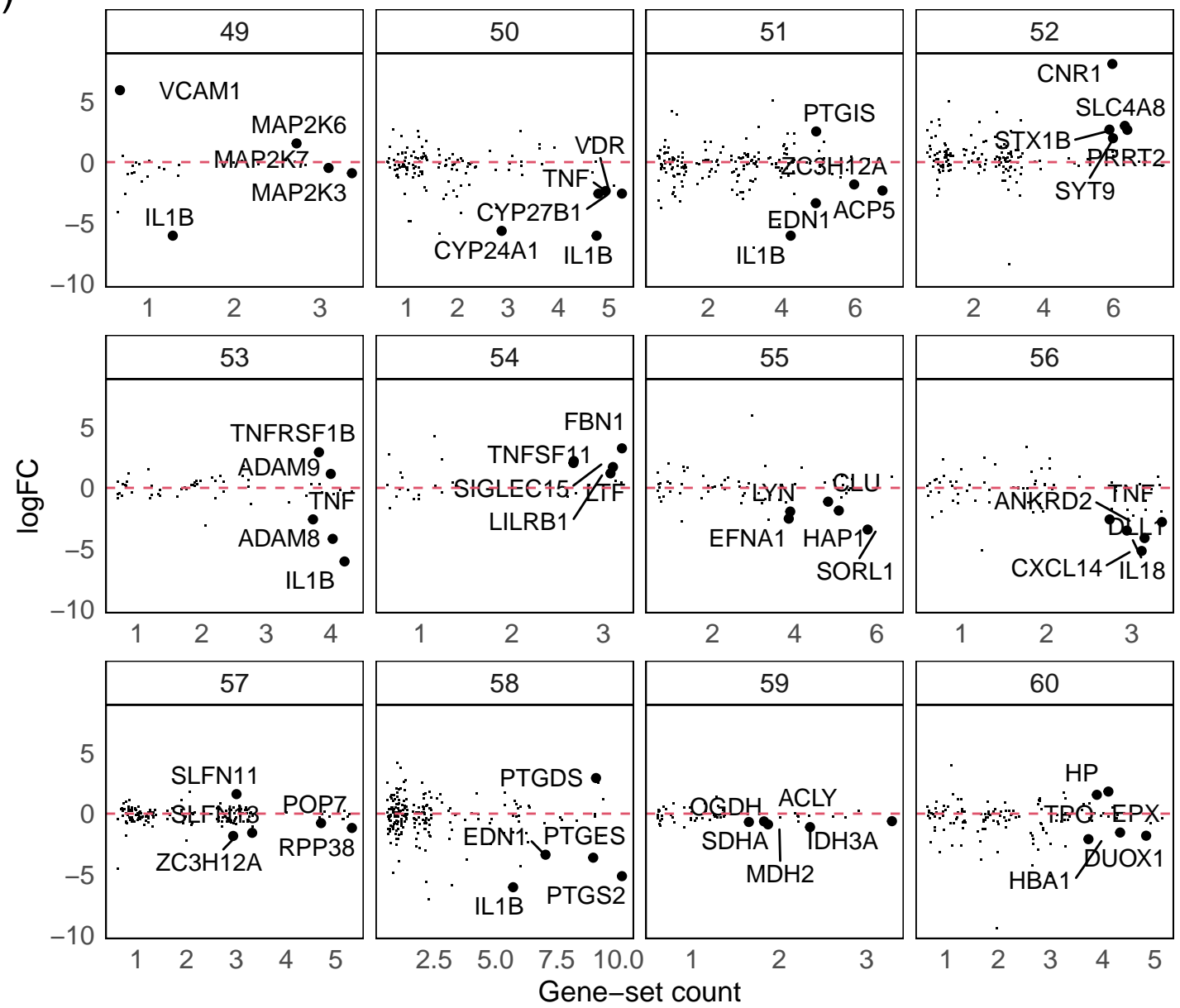

d)

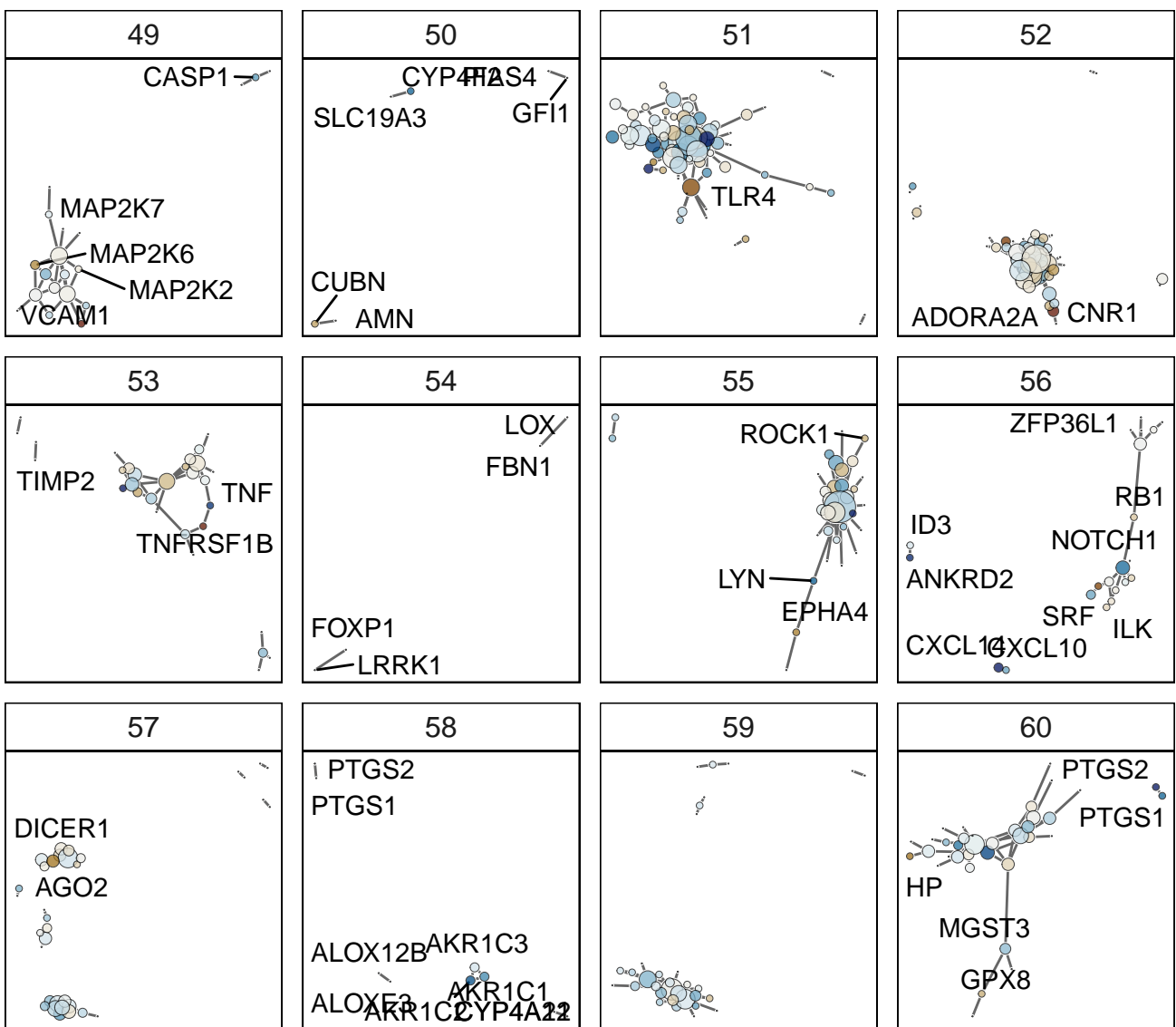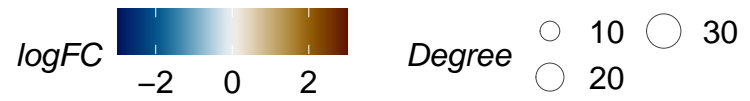

Supplement: Supplementary file 2 — Additional file 2: Top 60 vissE groups identified in the analysis of the bulk RNA-seq dataset. [file 12859_2024_5676_MOESM2_ESM.pdf]

a)

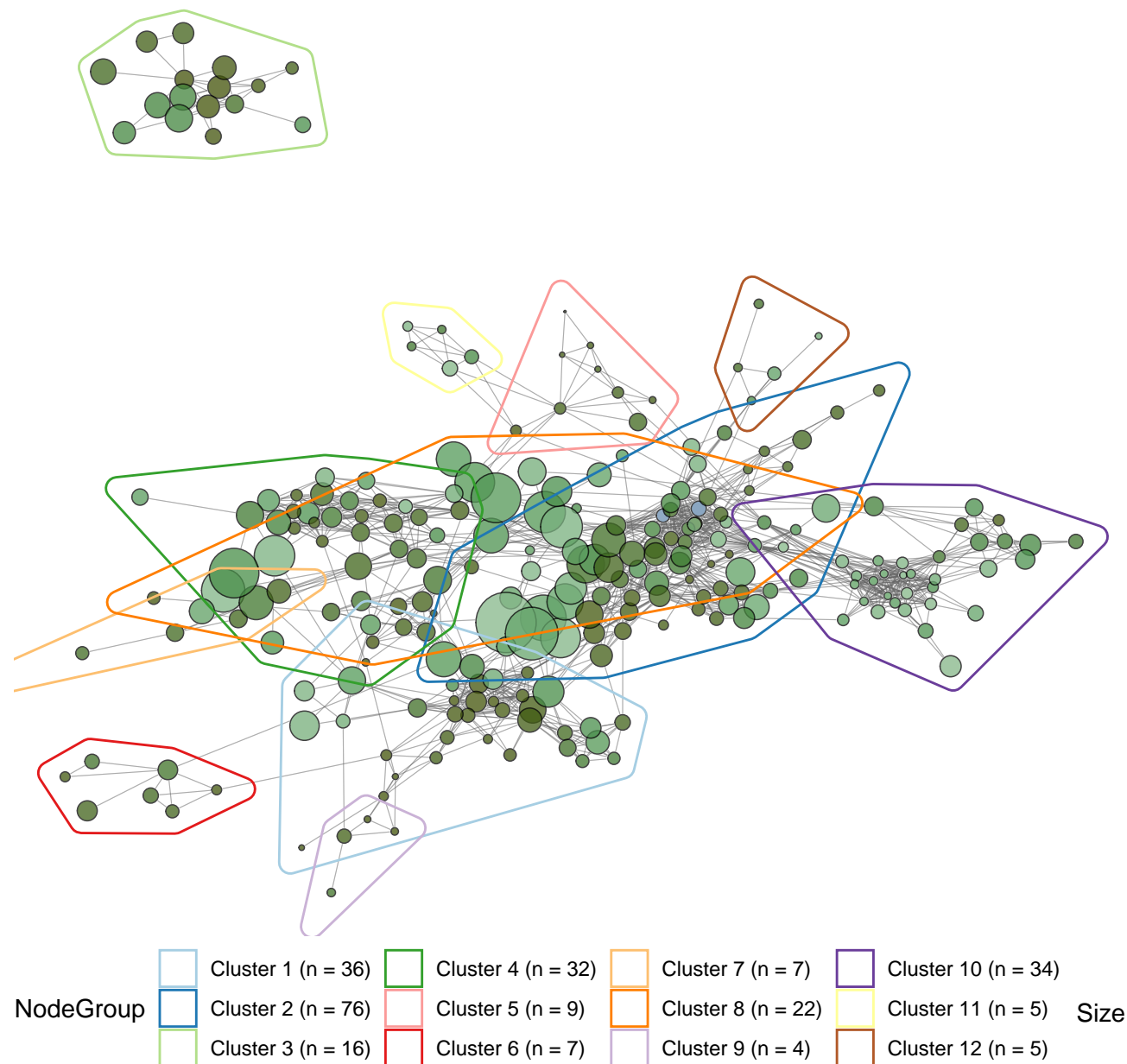

b)

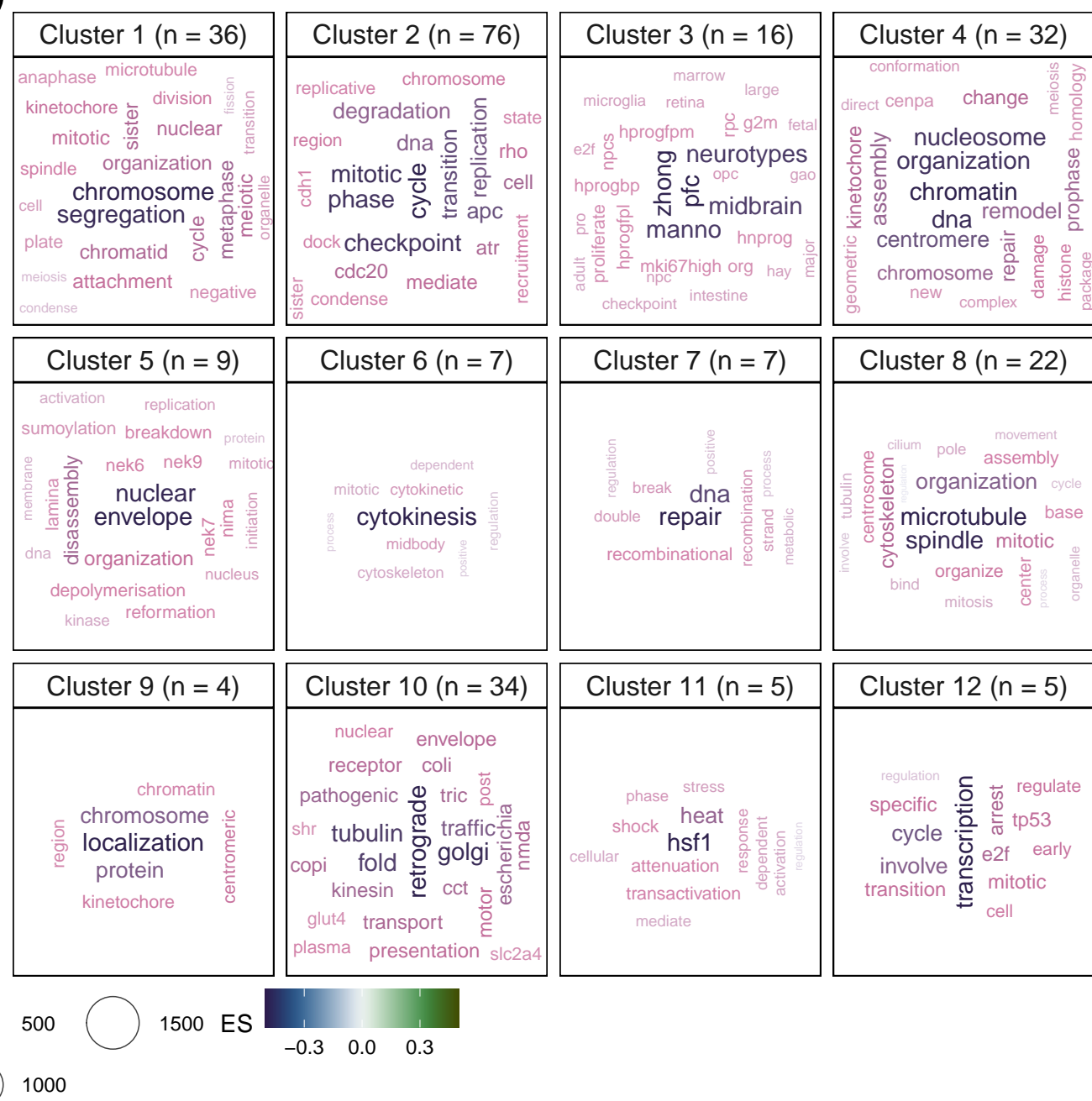

c)

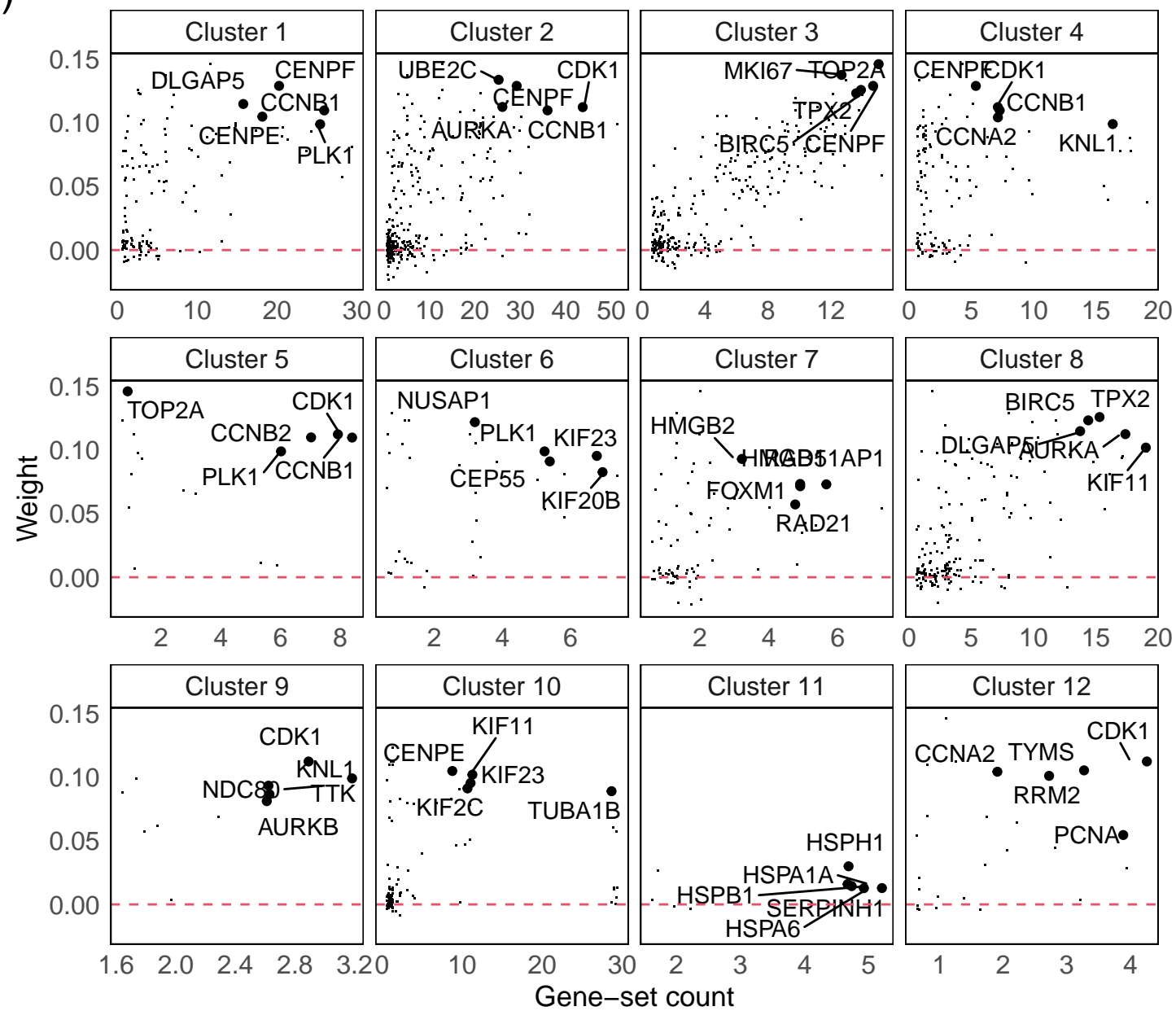

d)

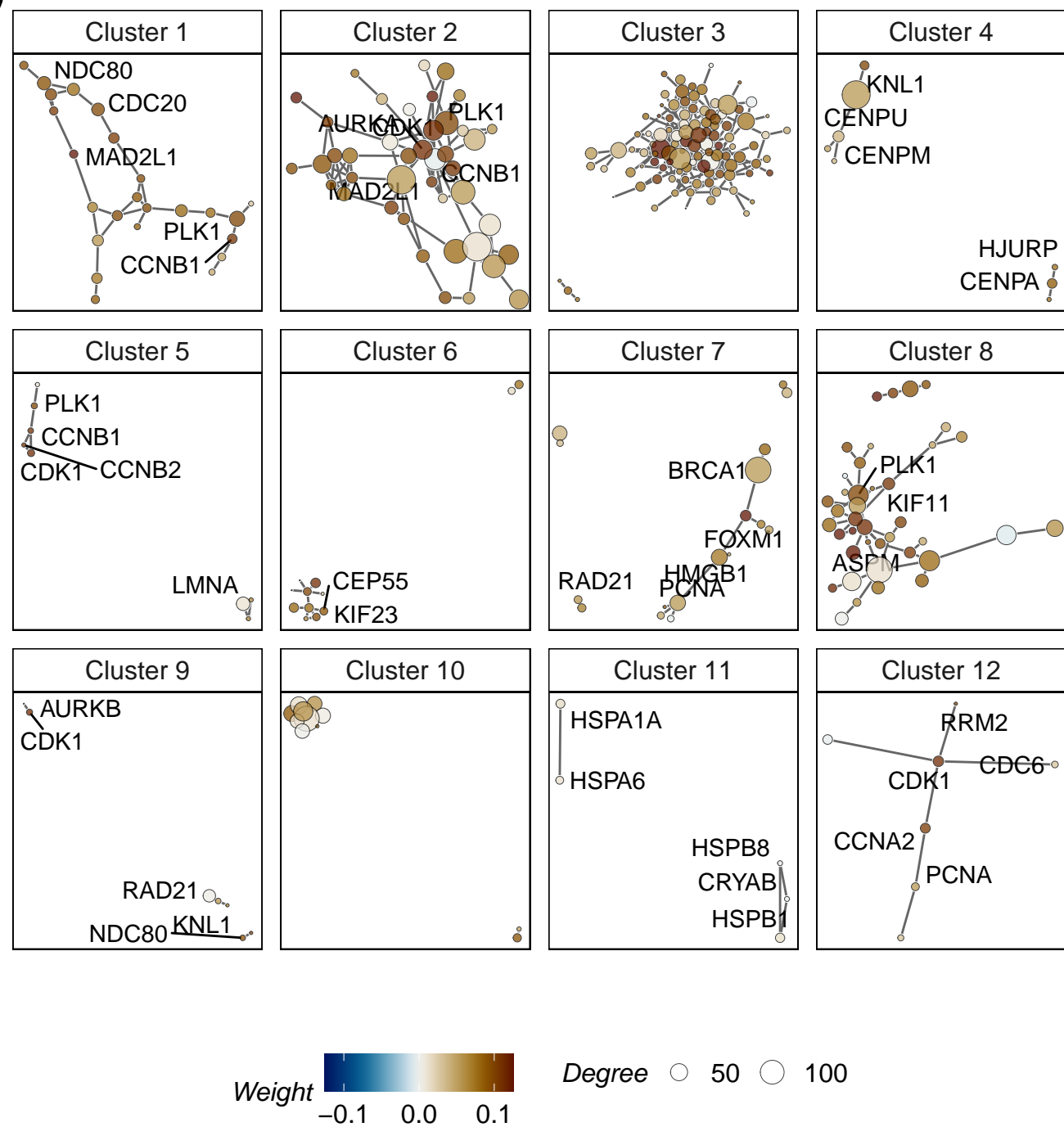

a)

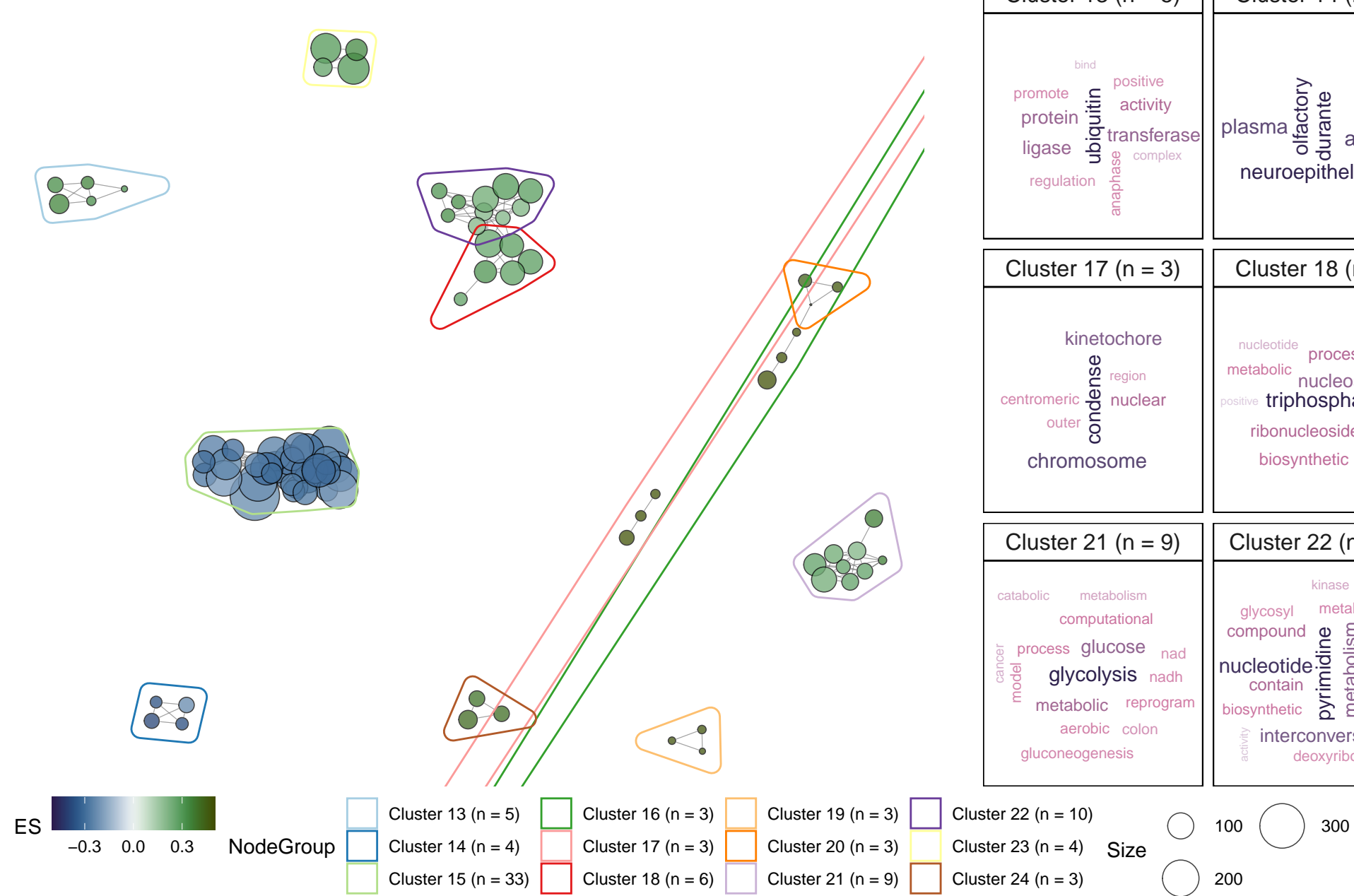

b)

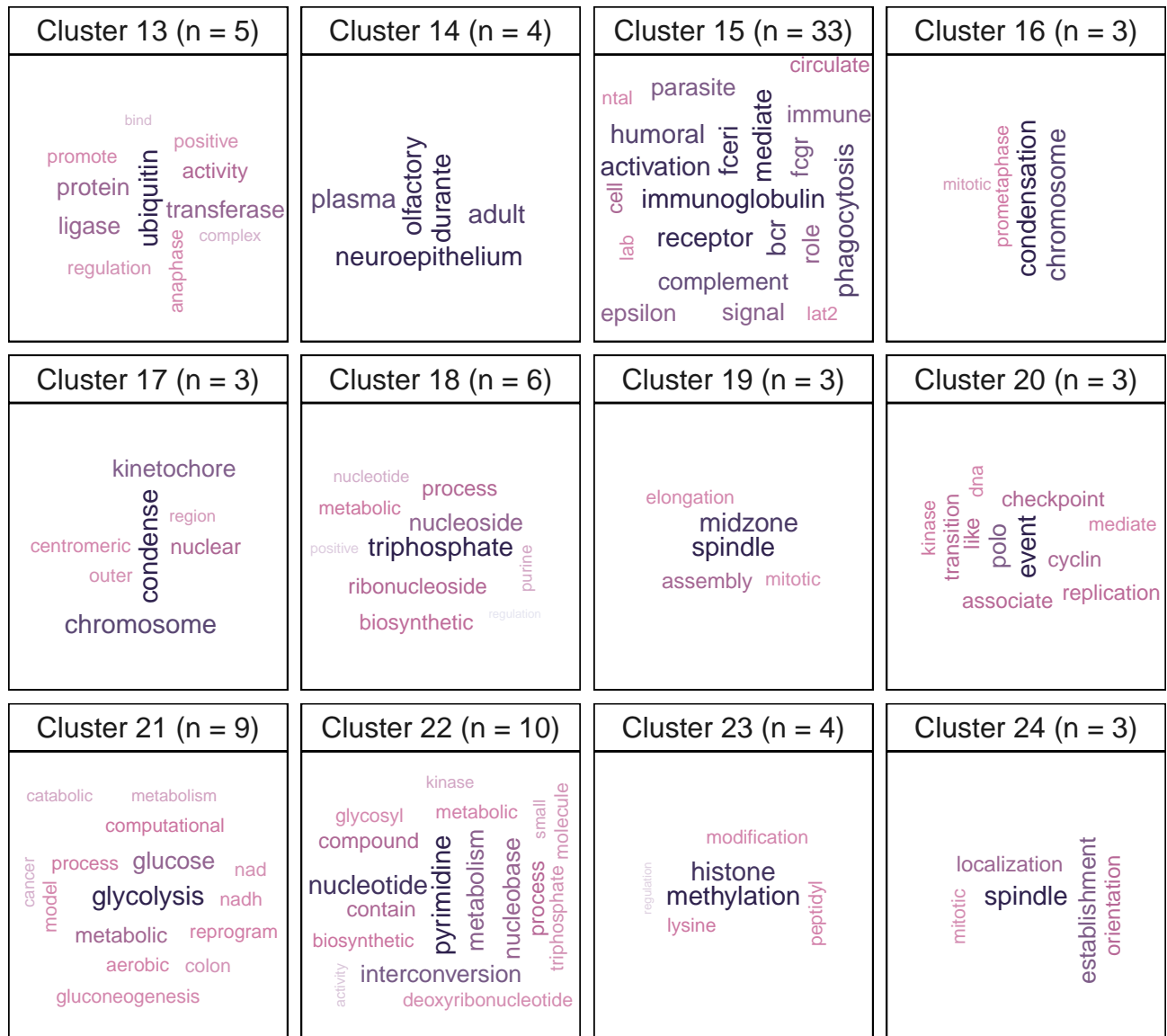

c)

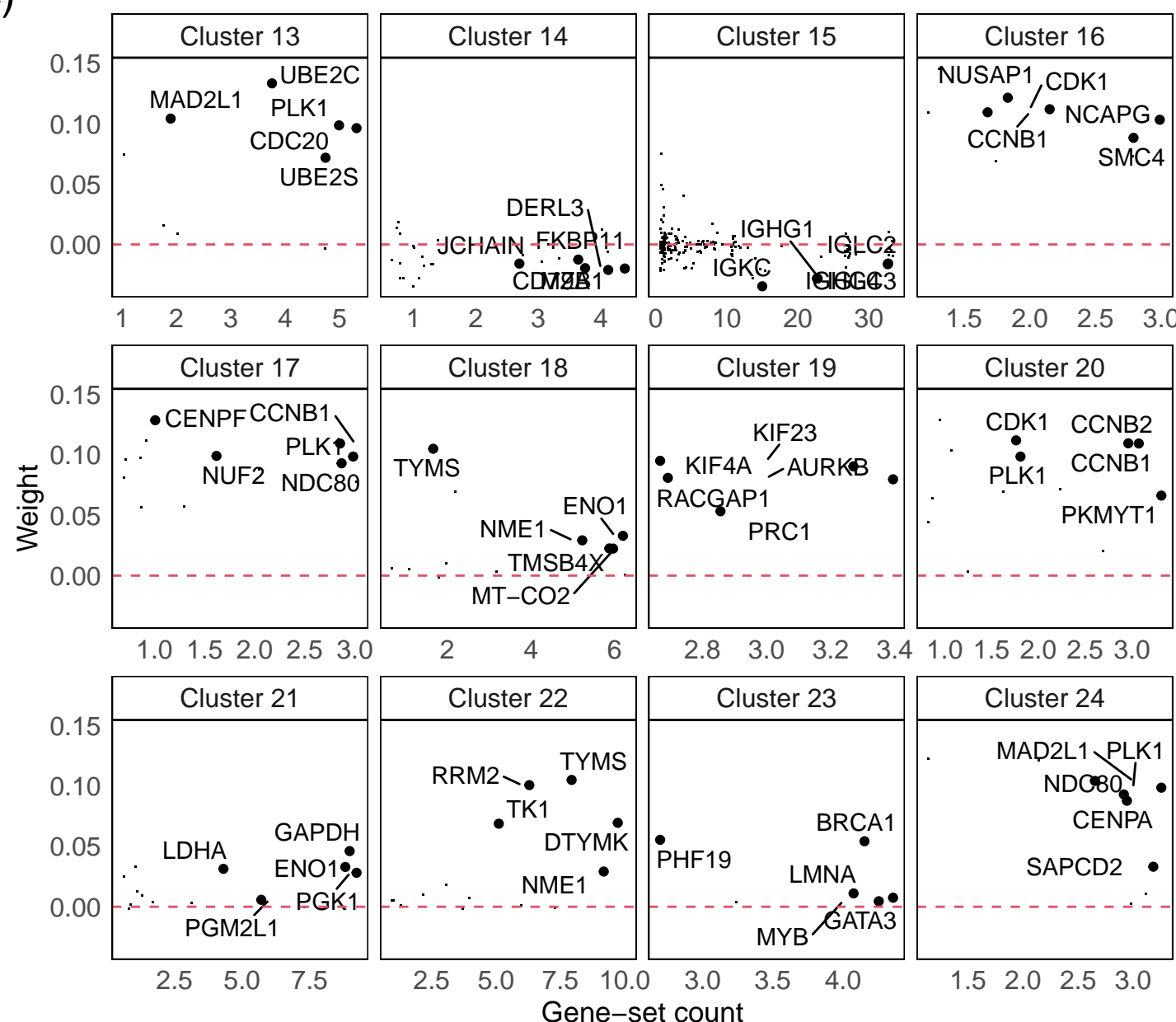

d)

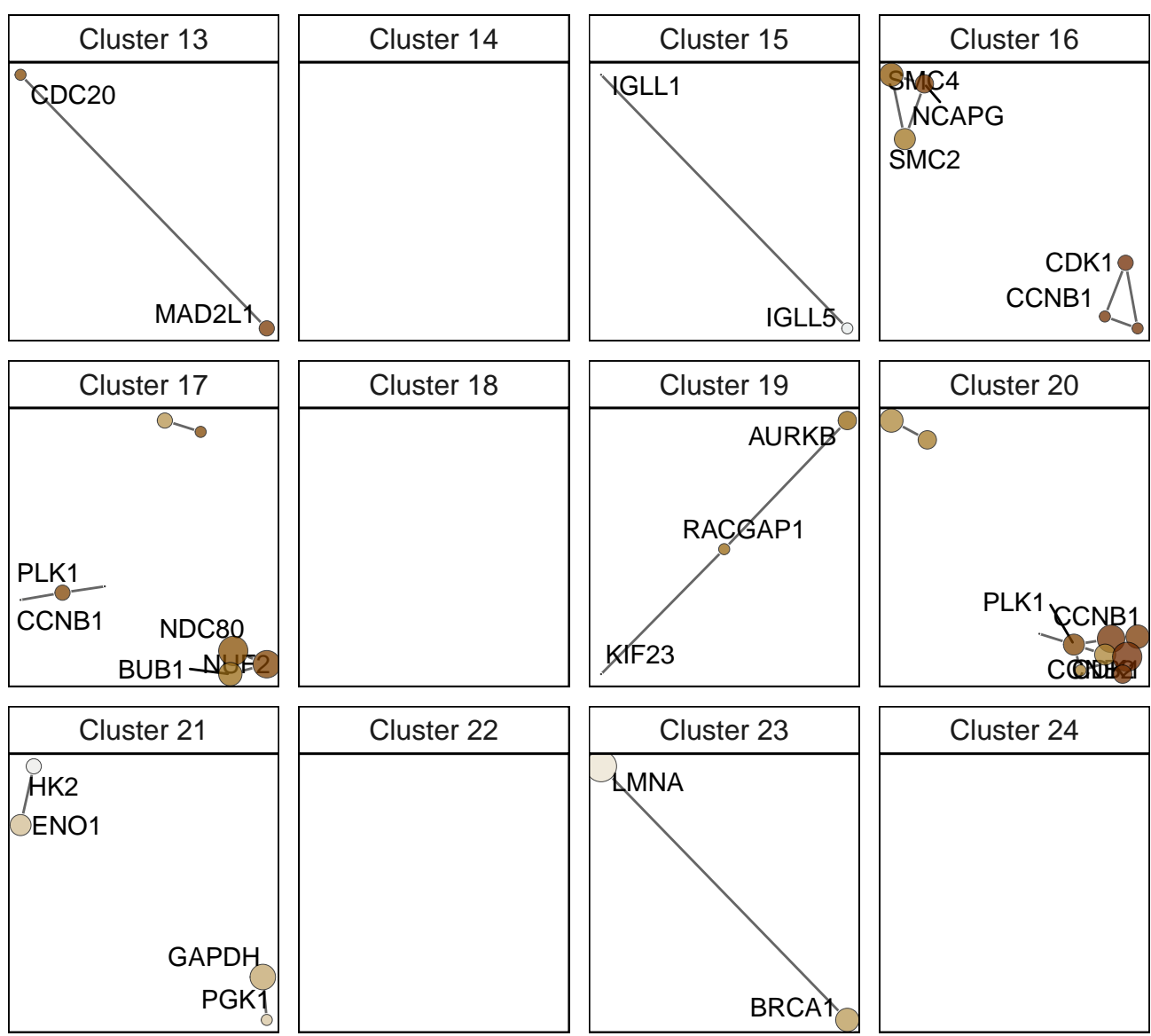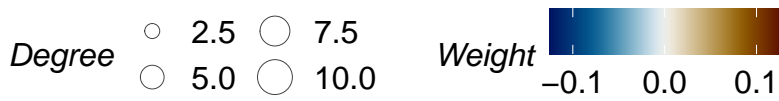

a)

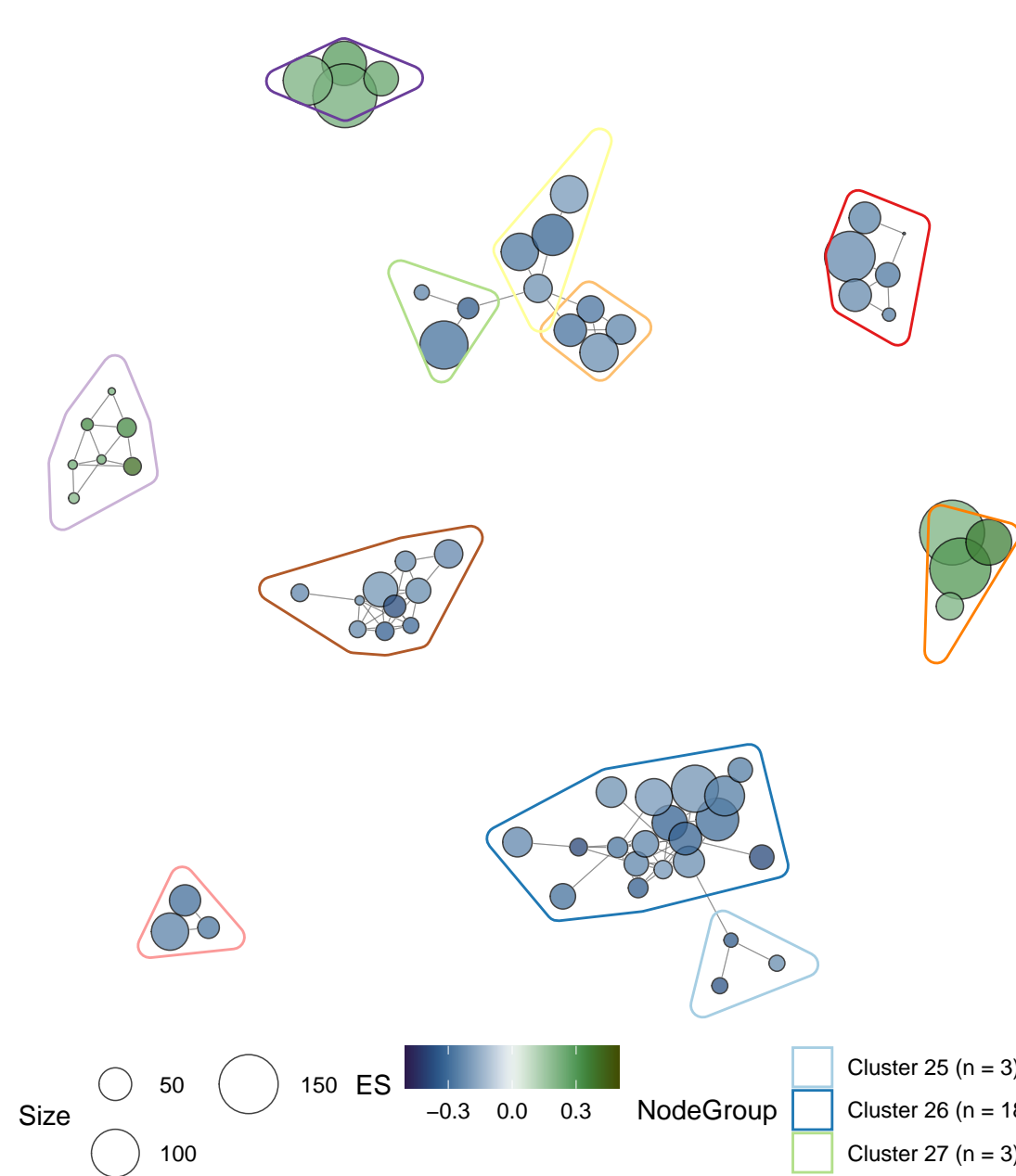

b)

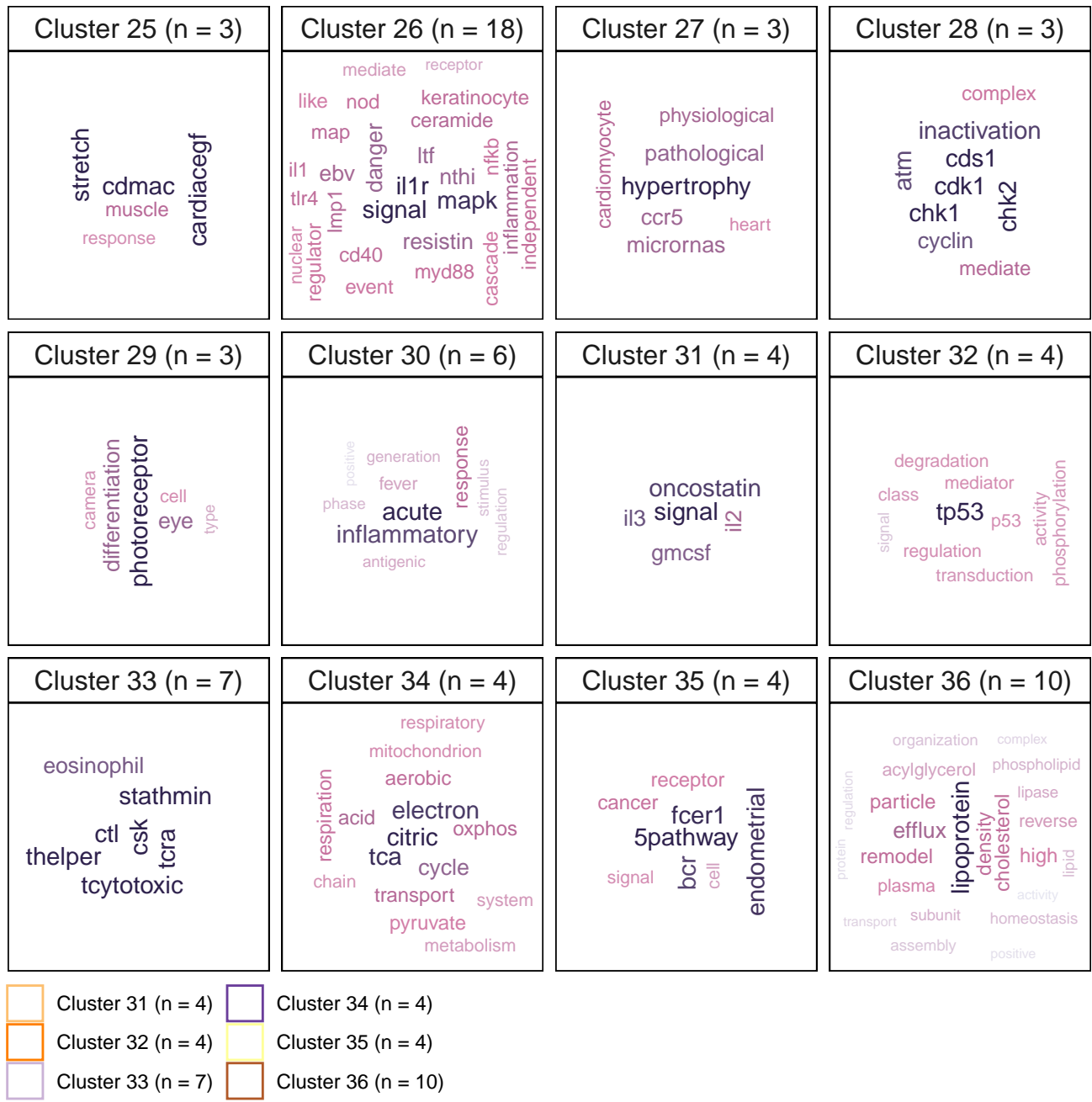

c)

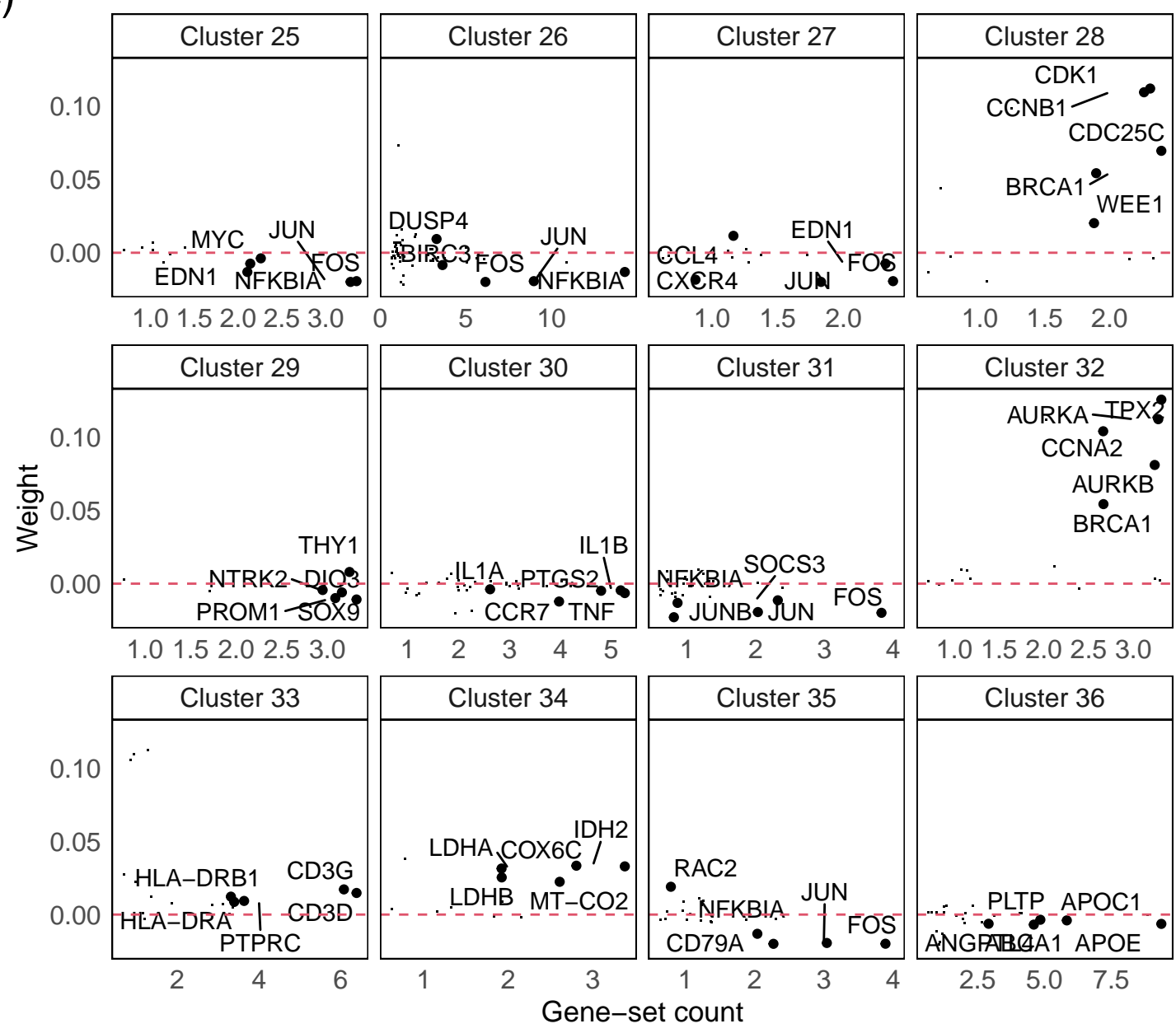

d)

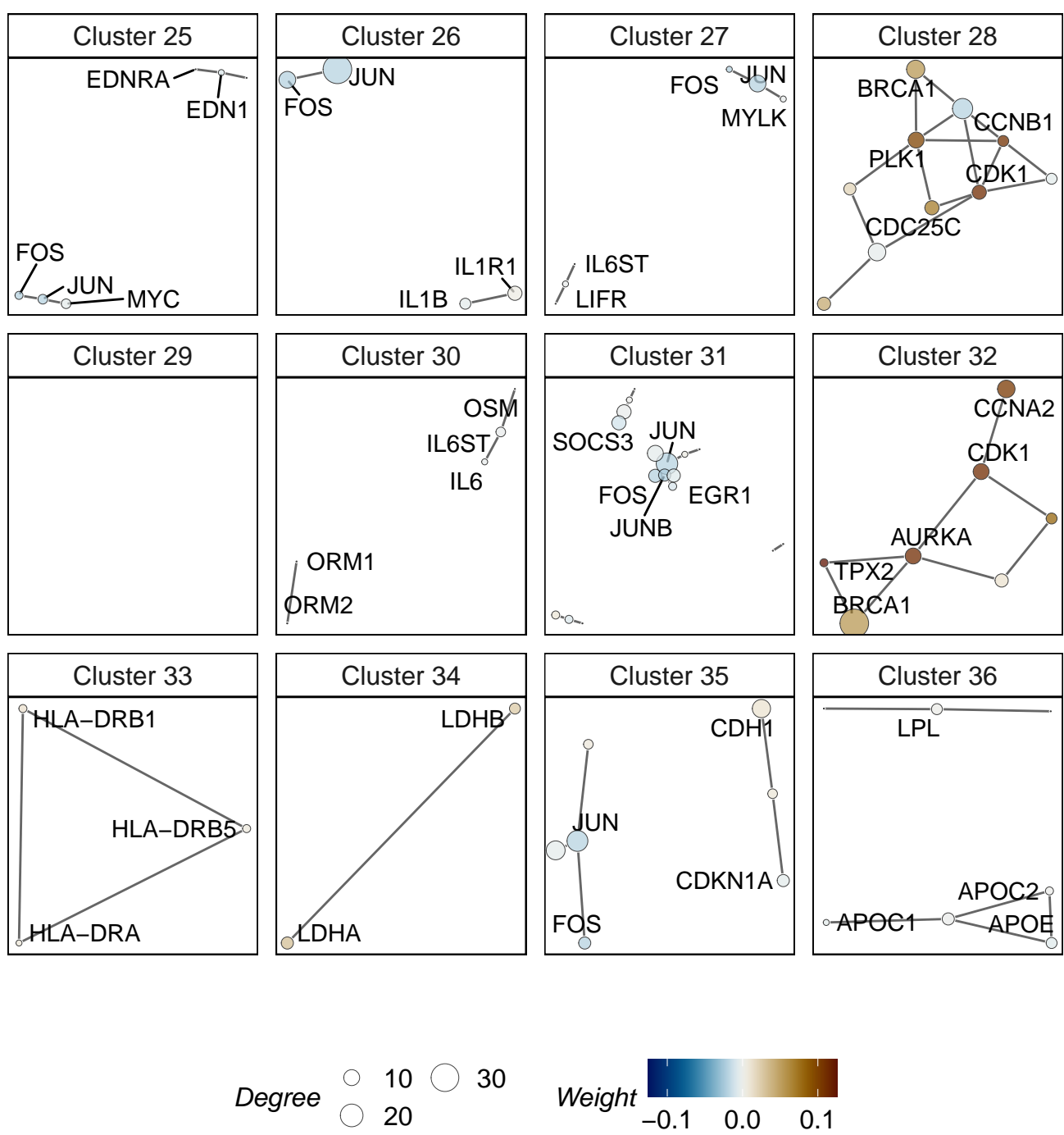

a)

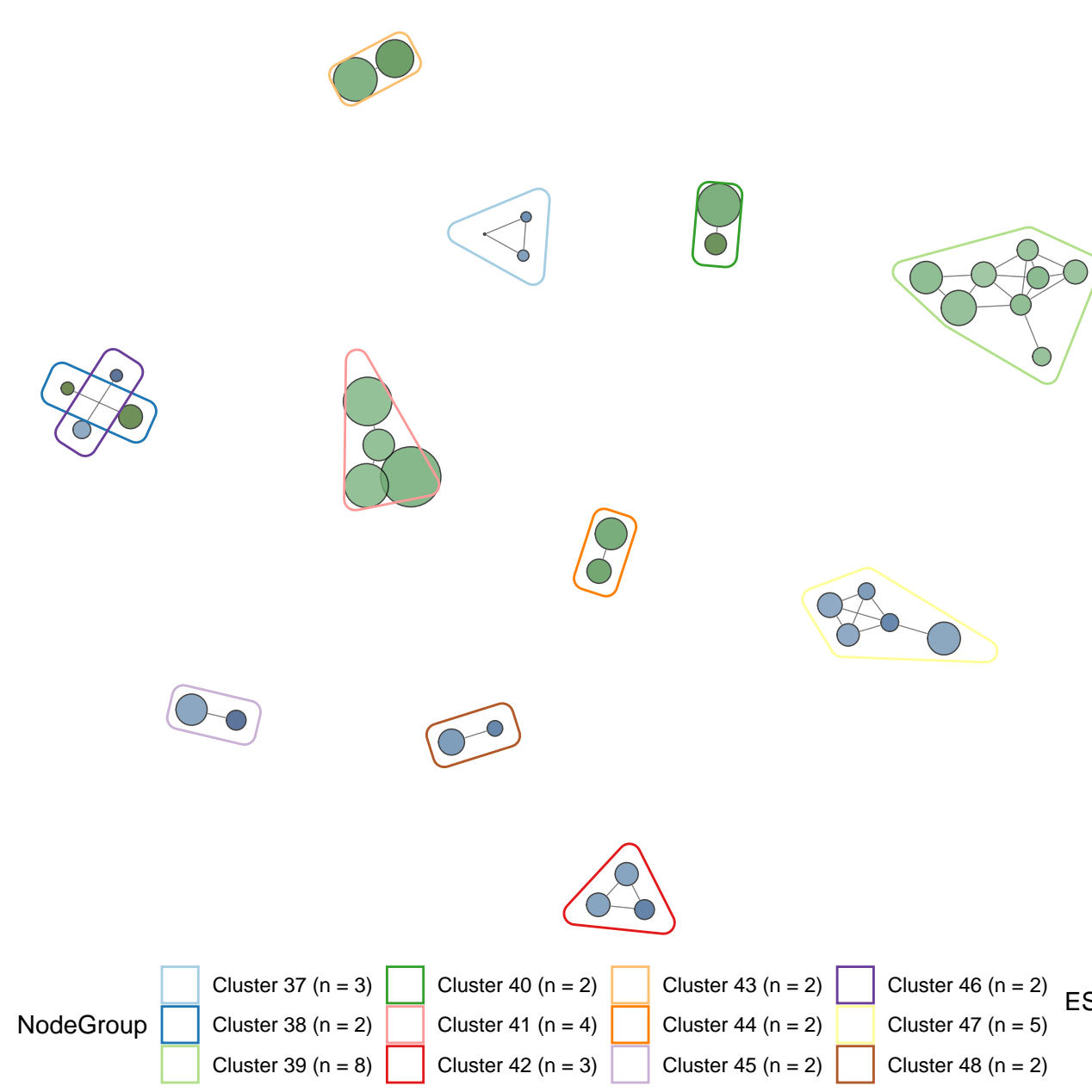

b)

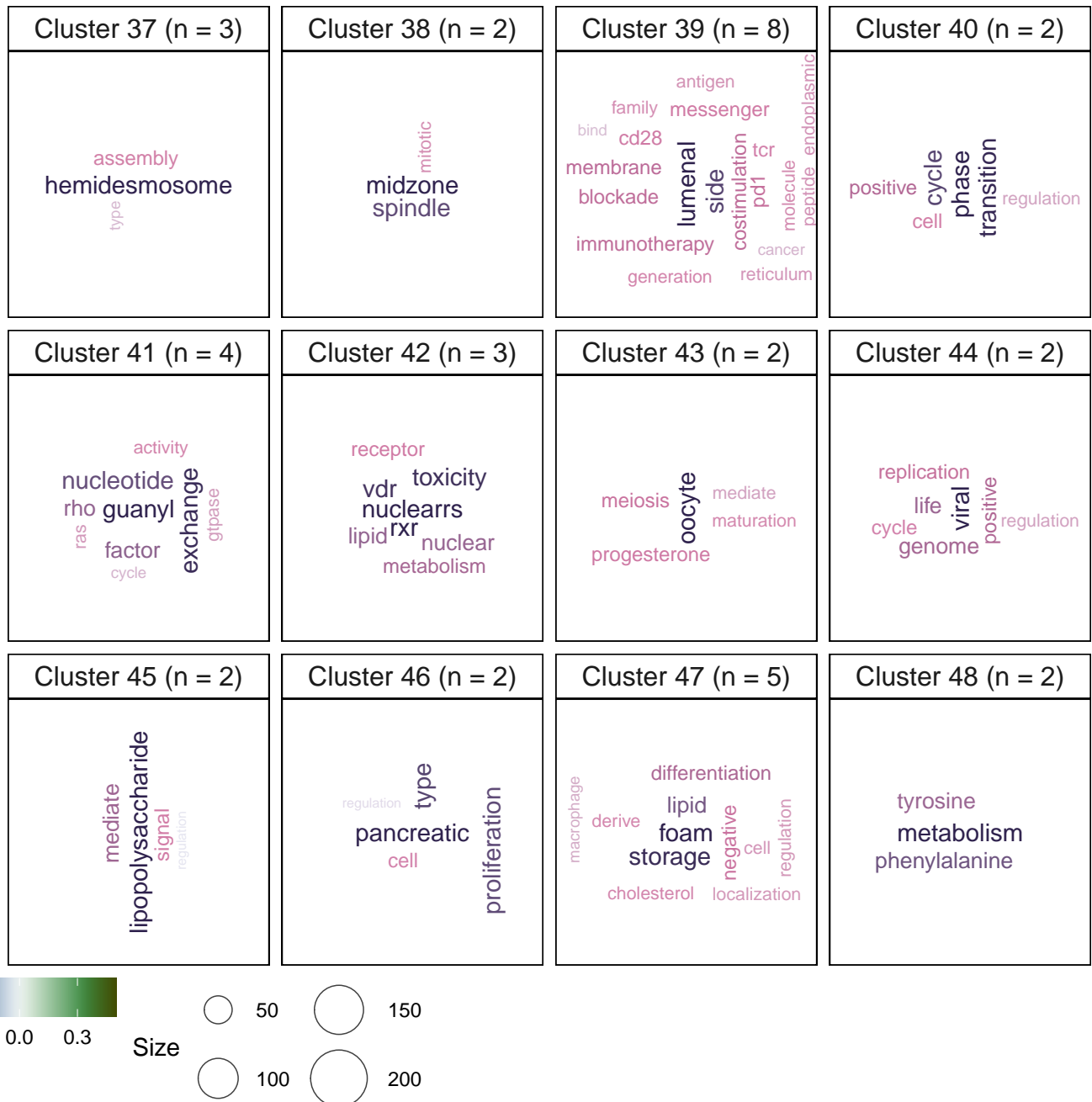

c)

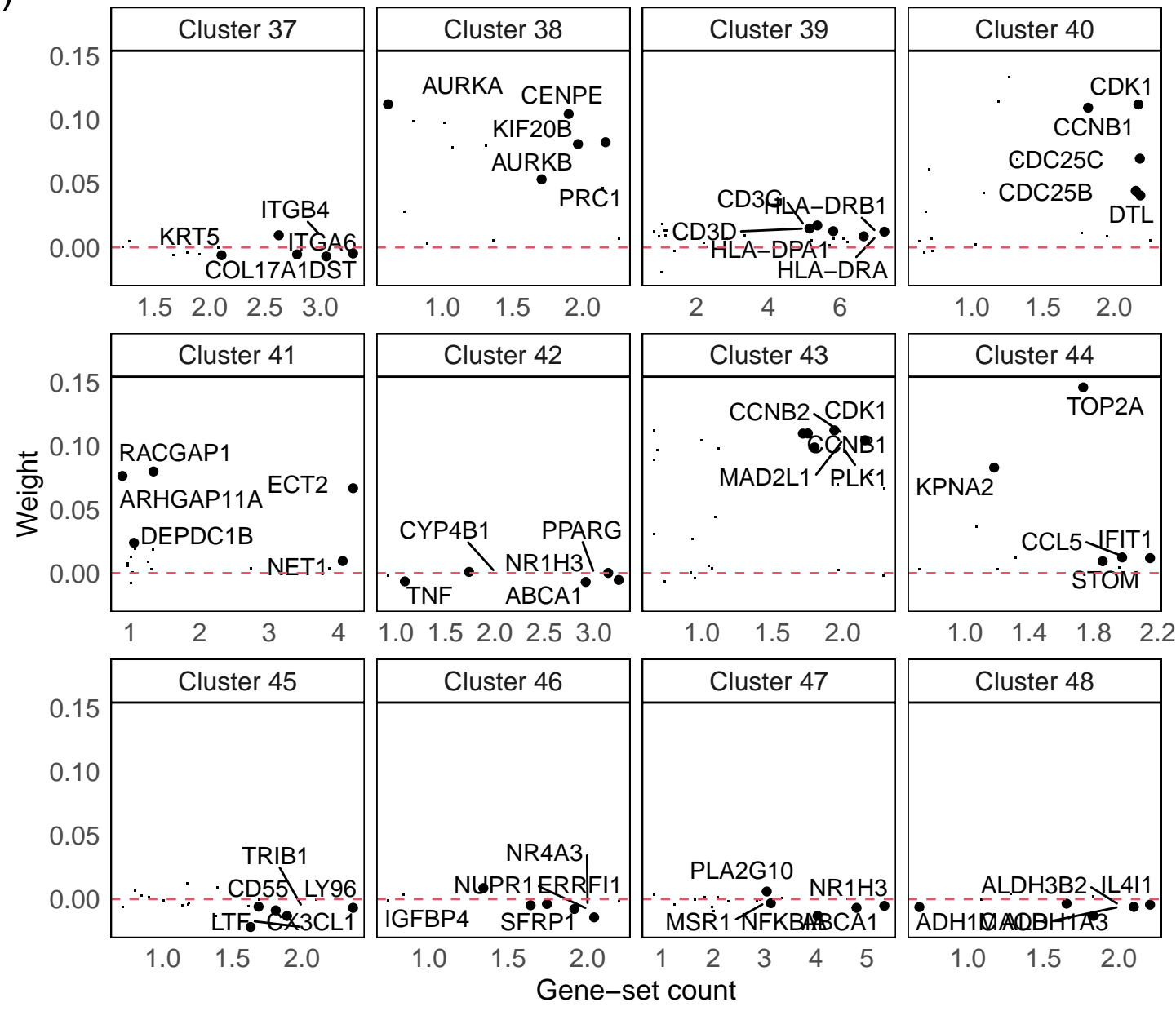

d)

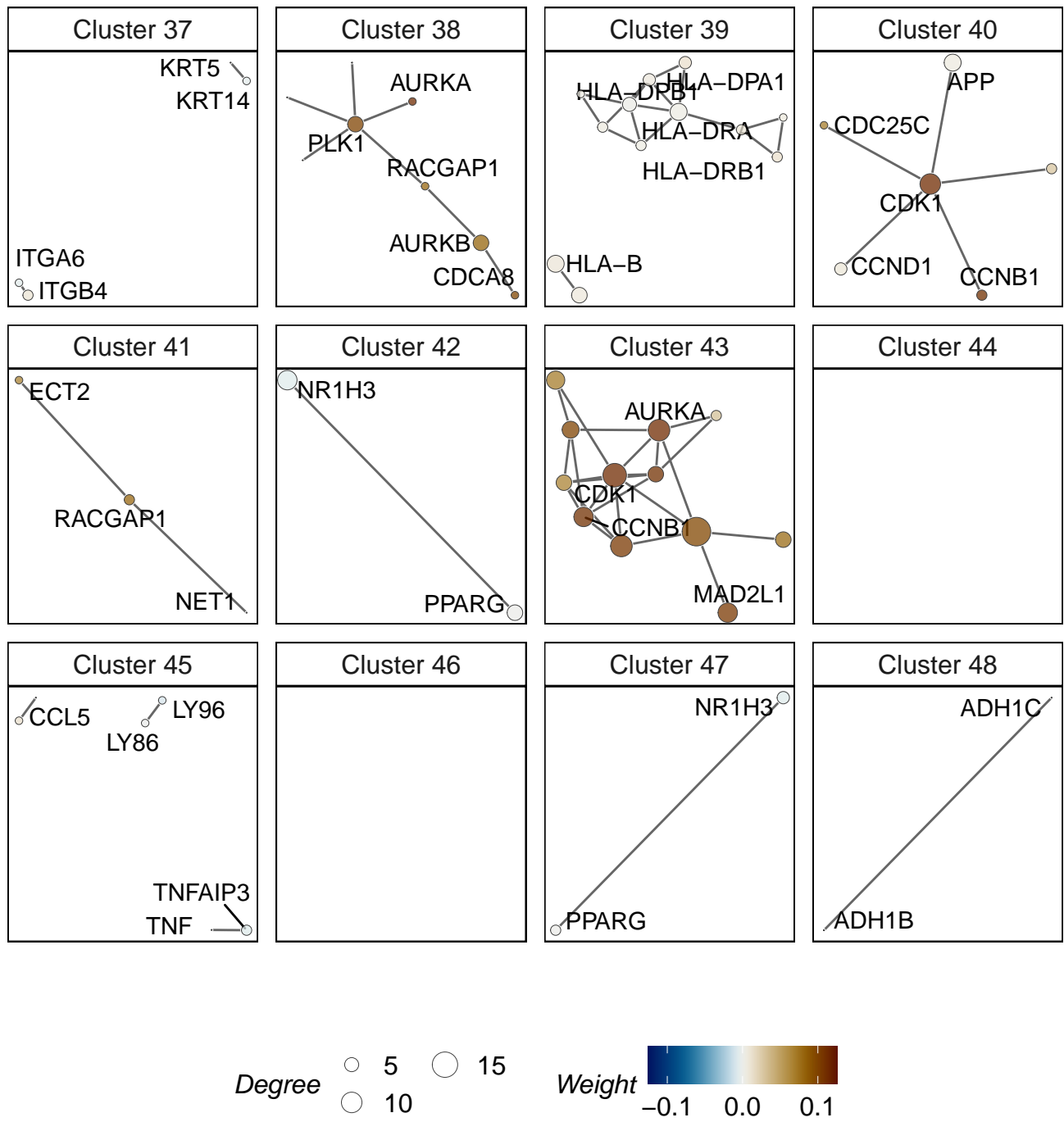

a)

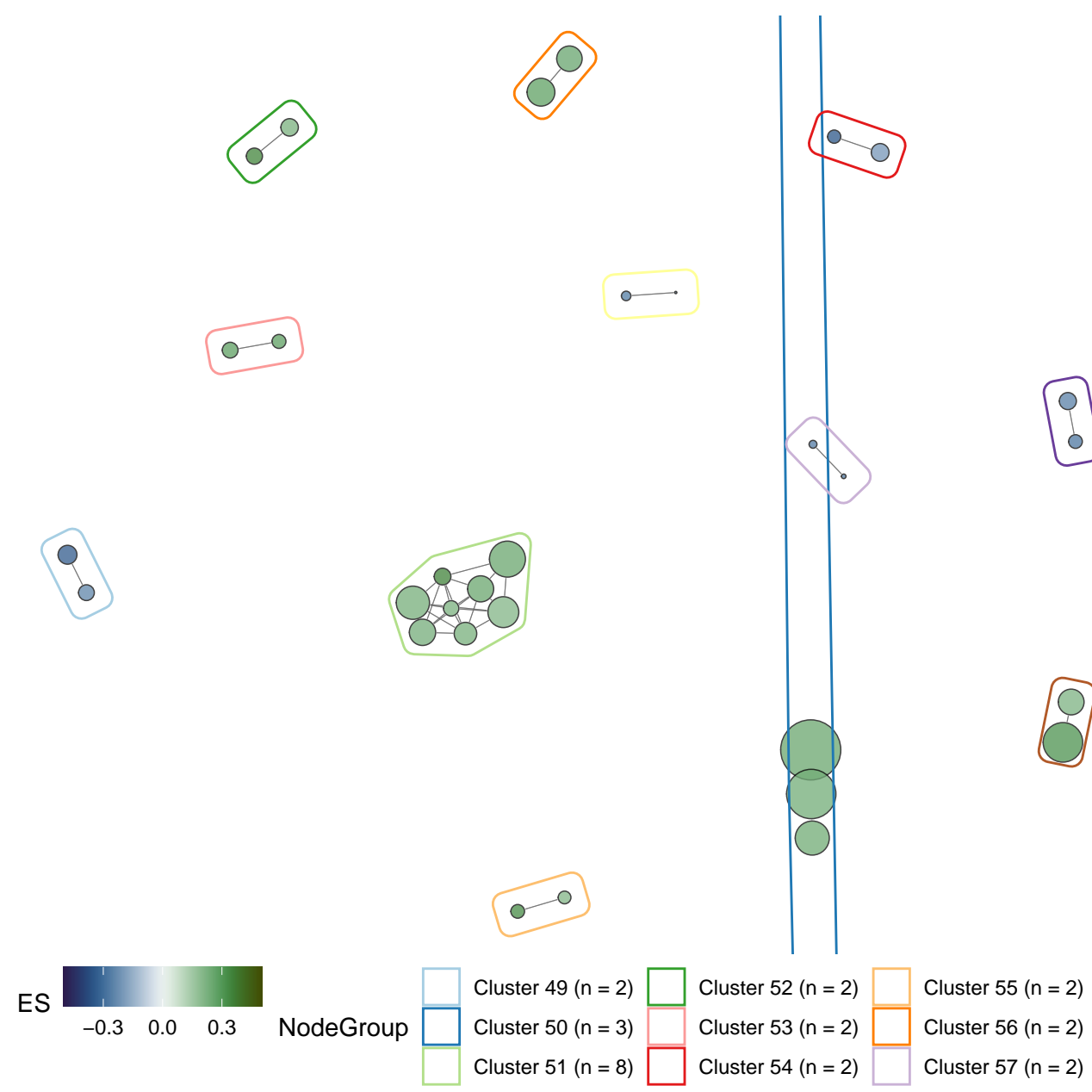

b)

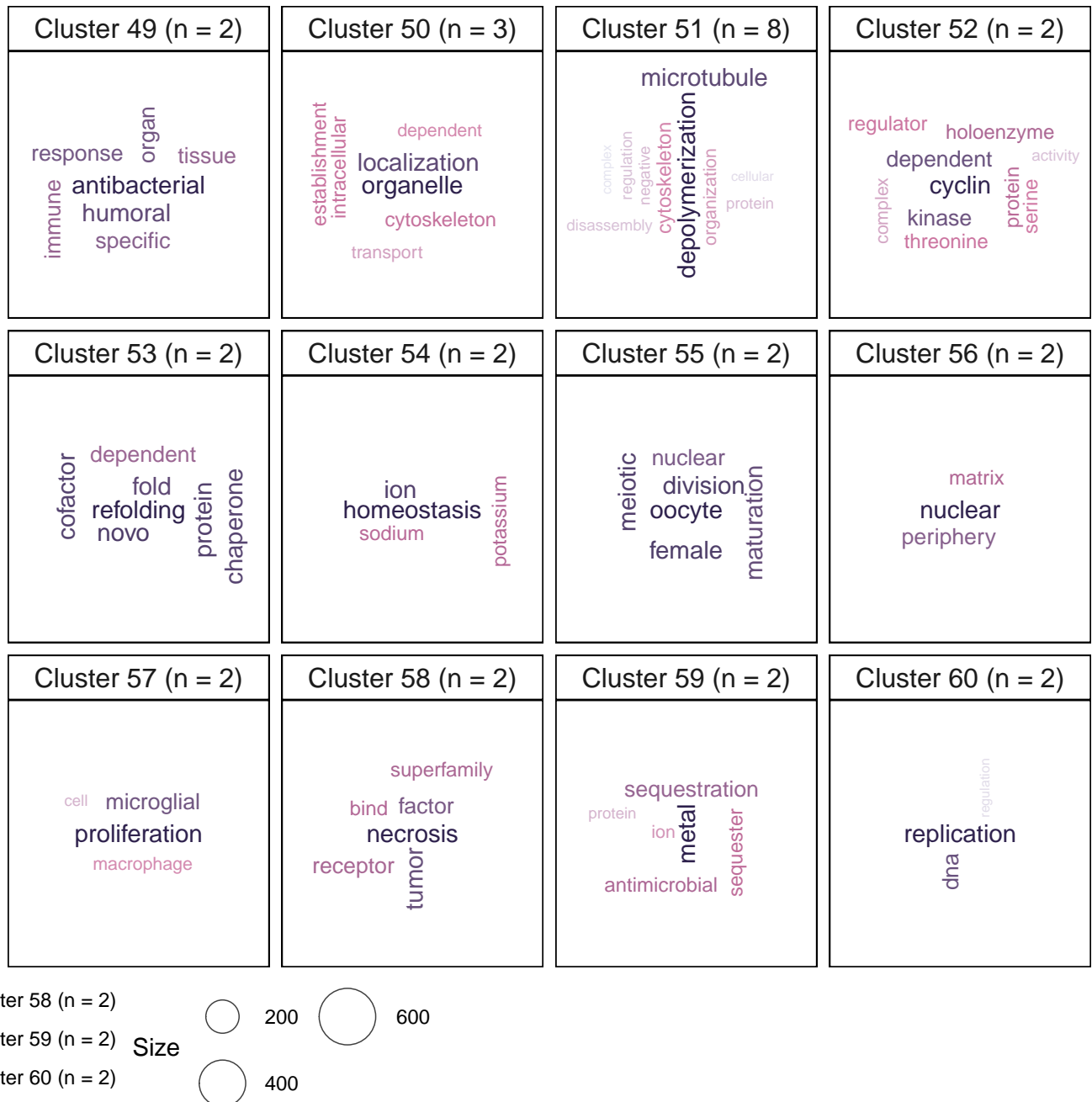

c)

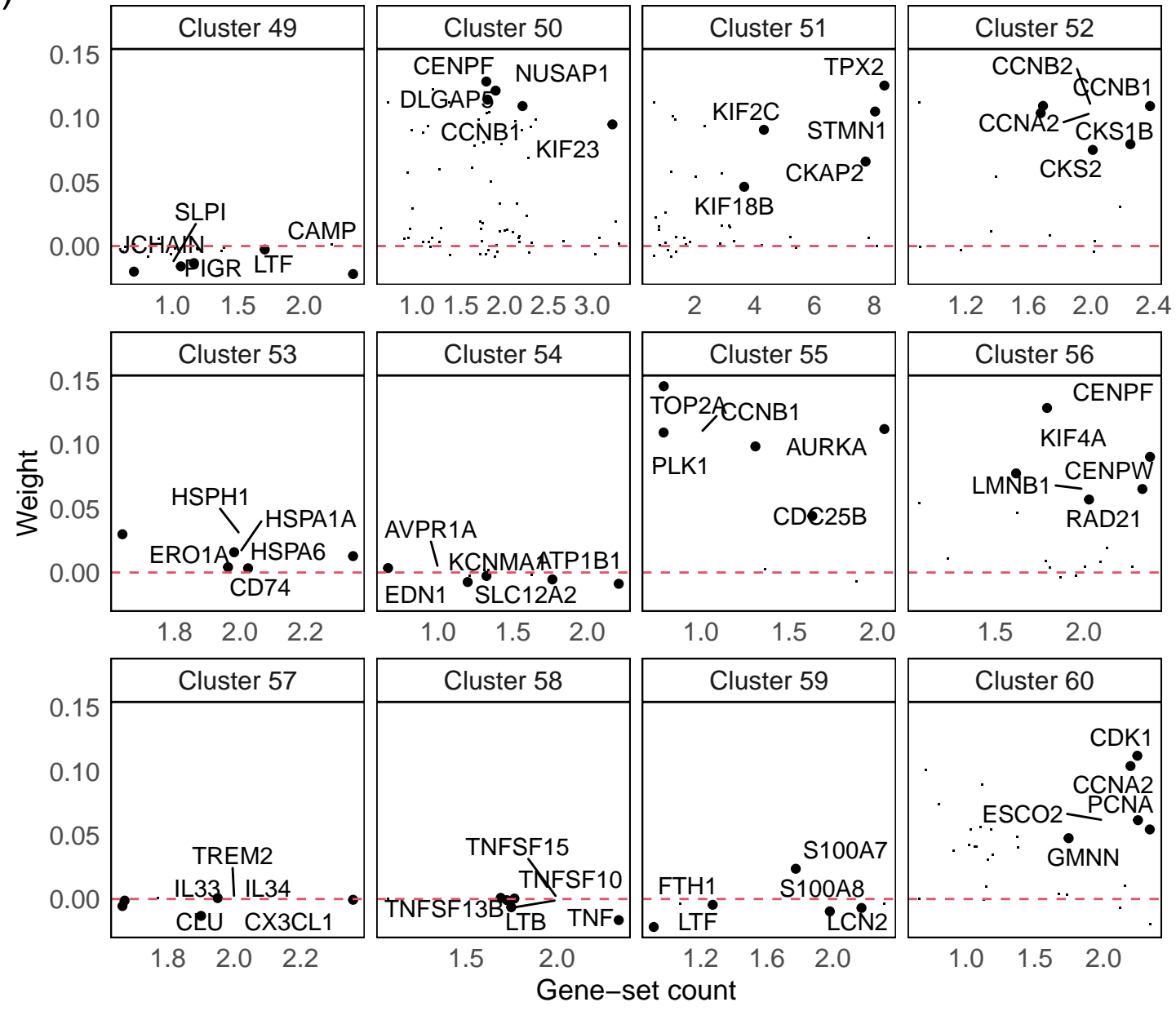

d)

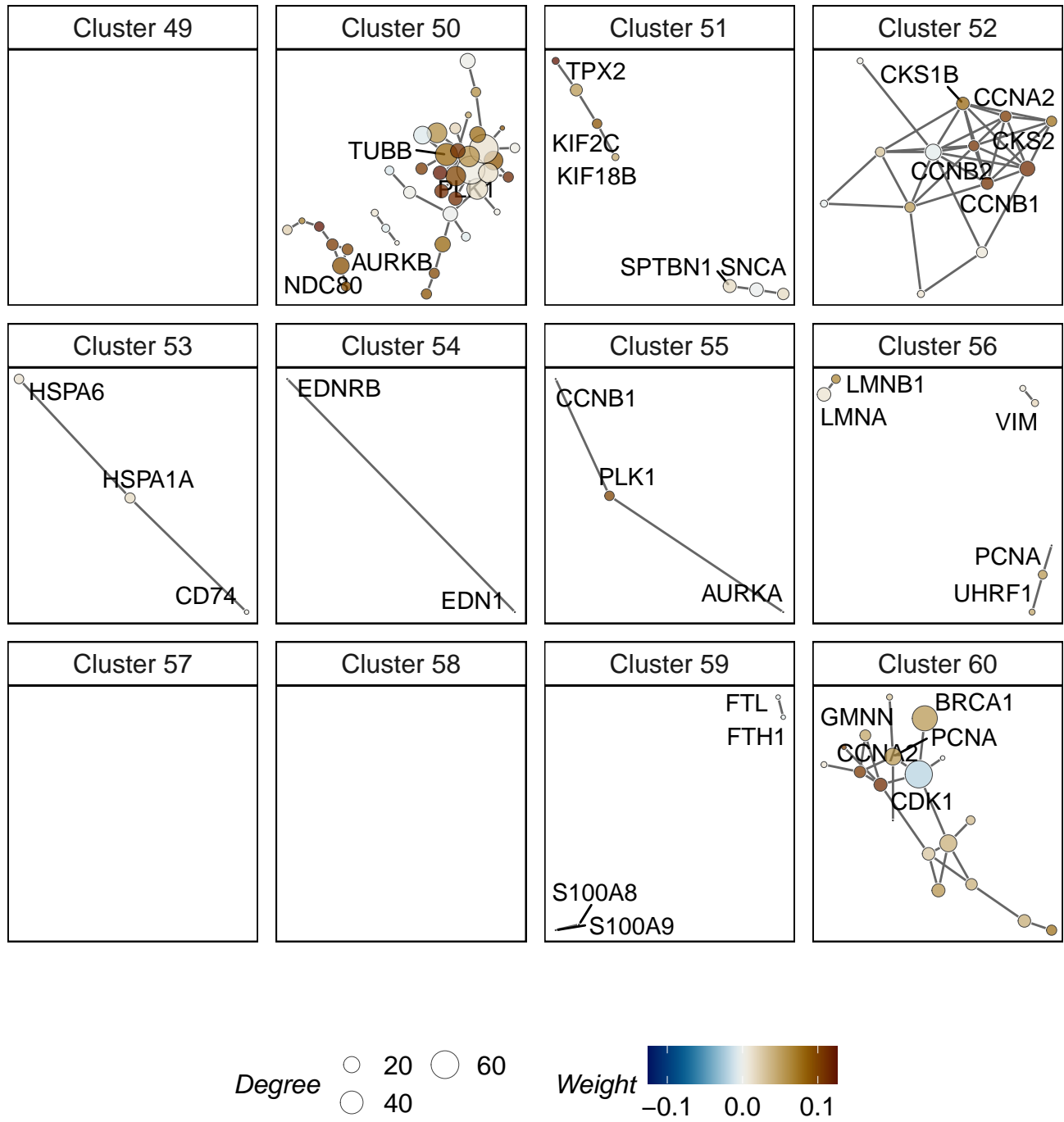

Supplement: Supplementary file 3 — Additional file 3: Top 60 vissE groups identified in the analysis of the single-cell RNA-seq dataset. [file 12859_2024_5676_MOESM3_ESM.pdf]

a)

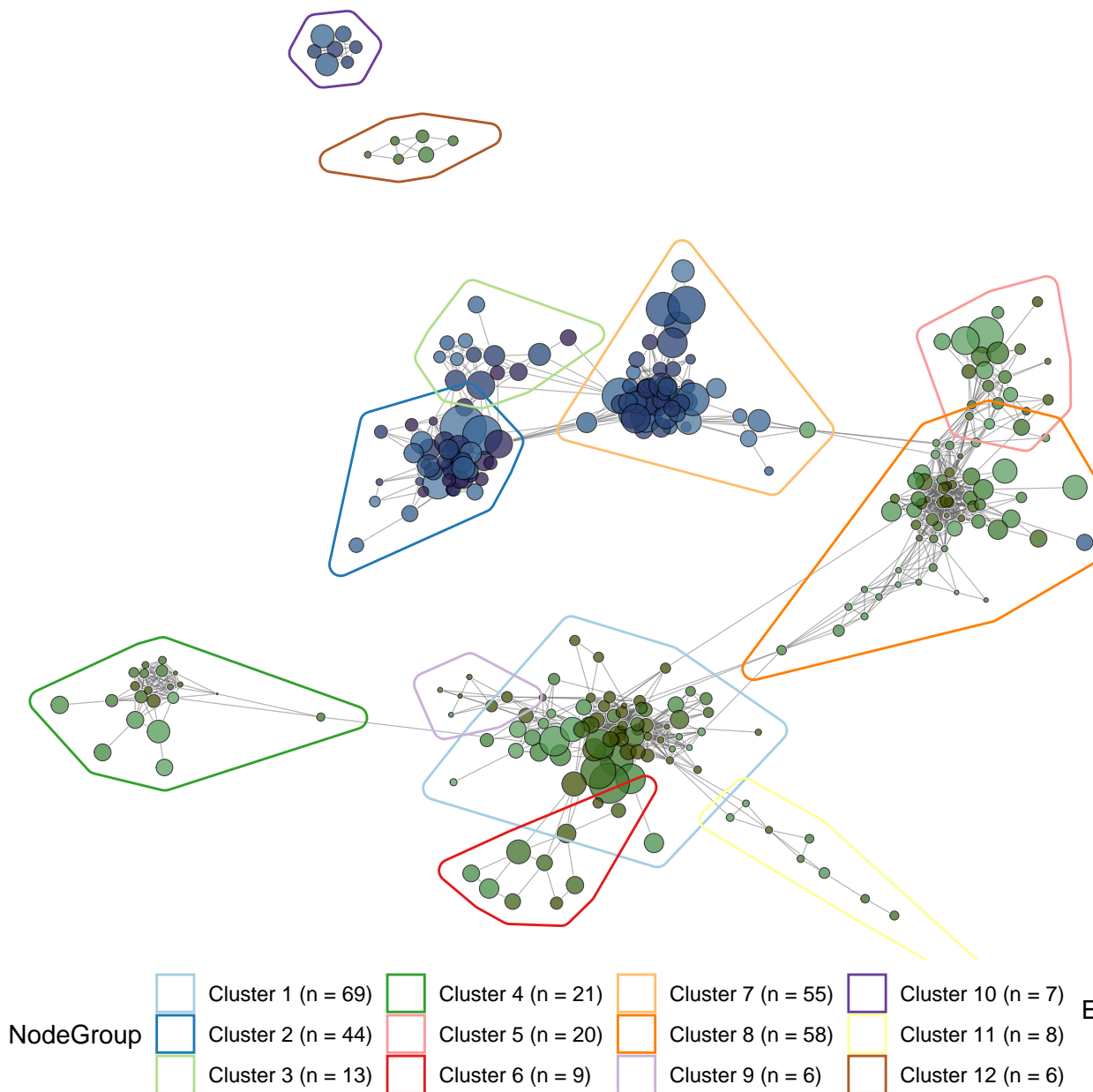

b)

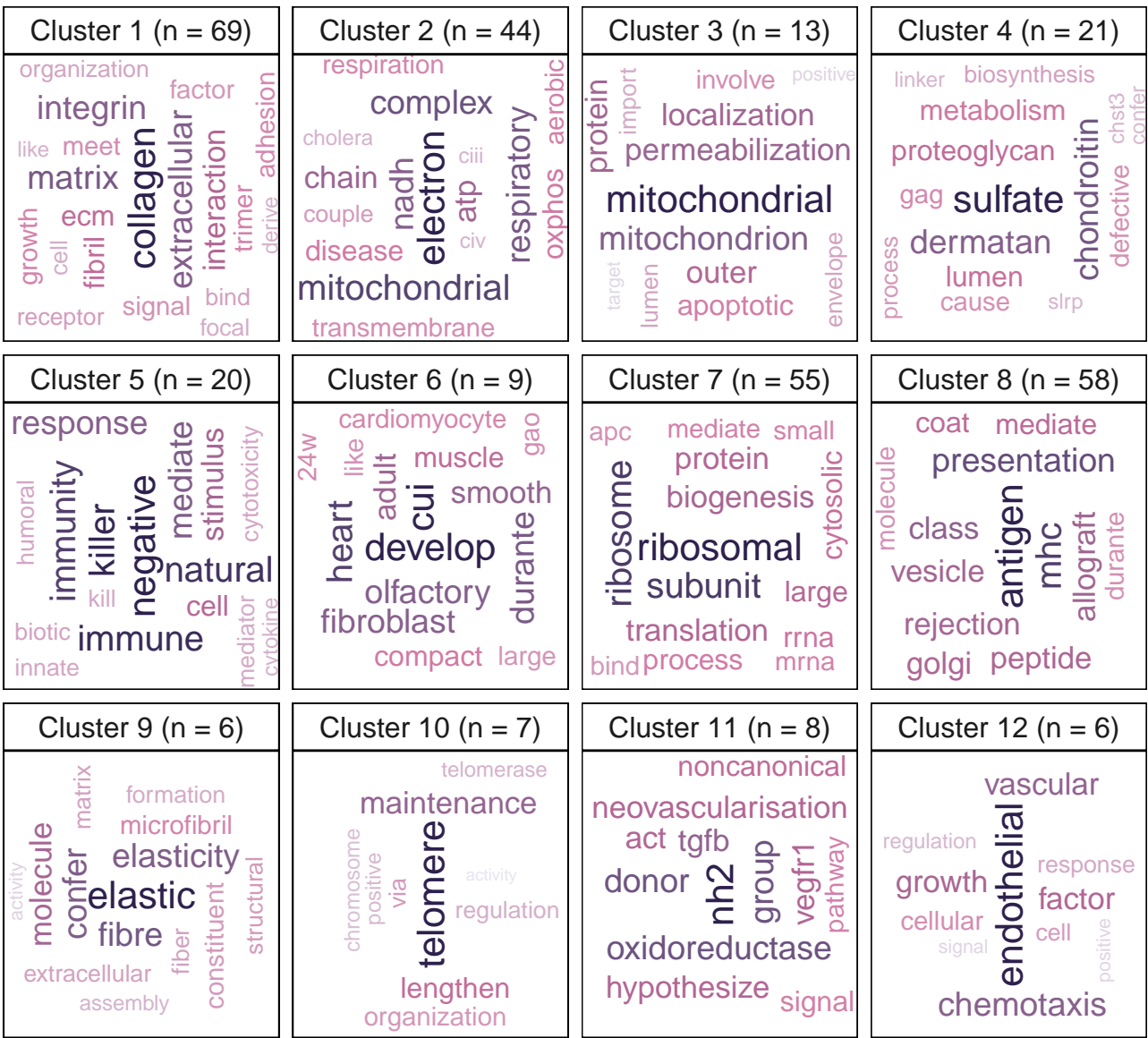

c)

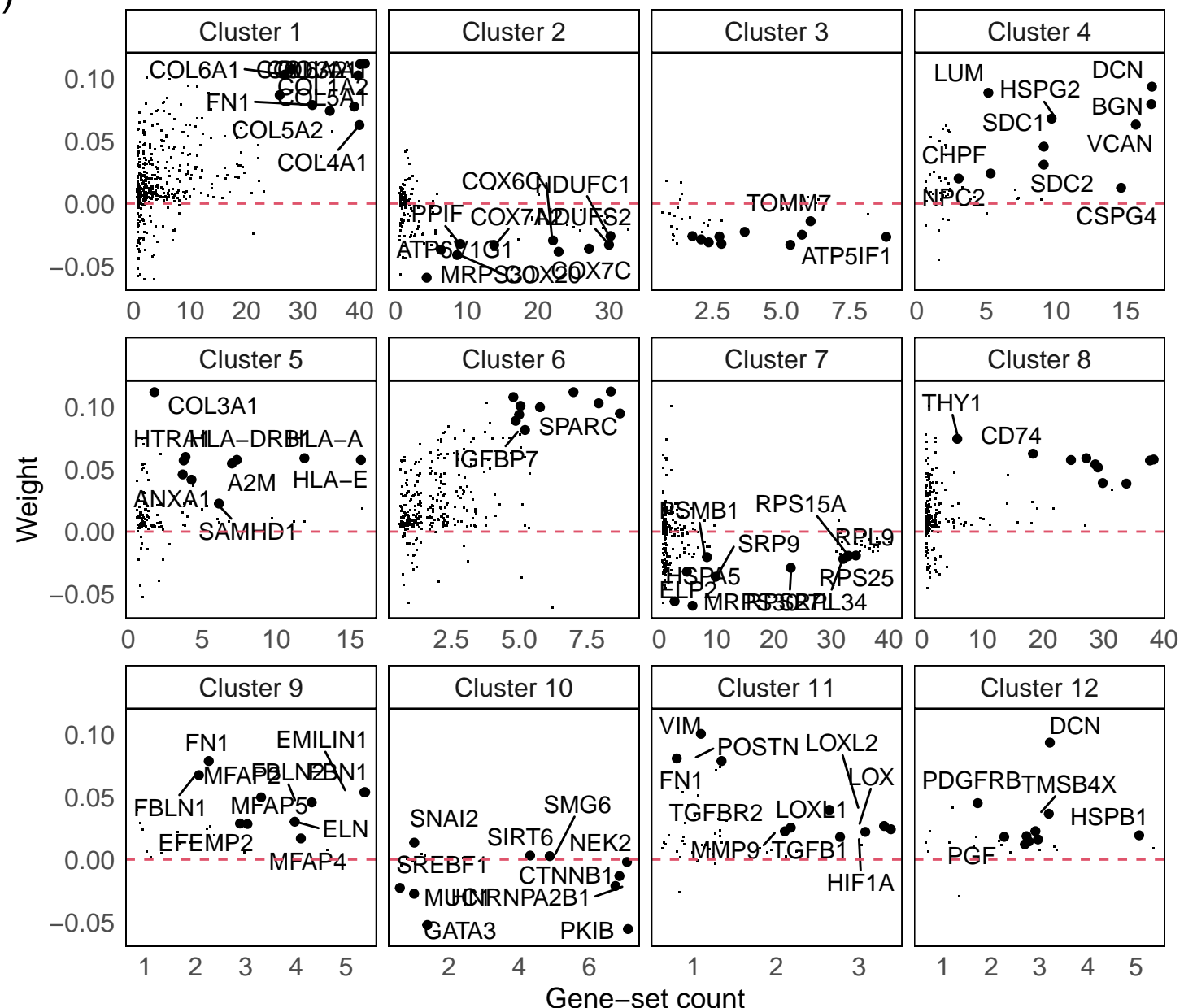

d)

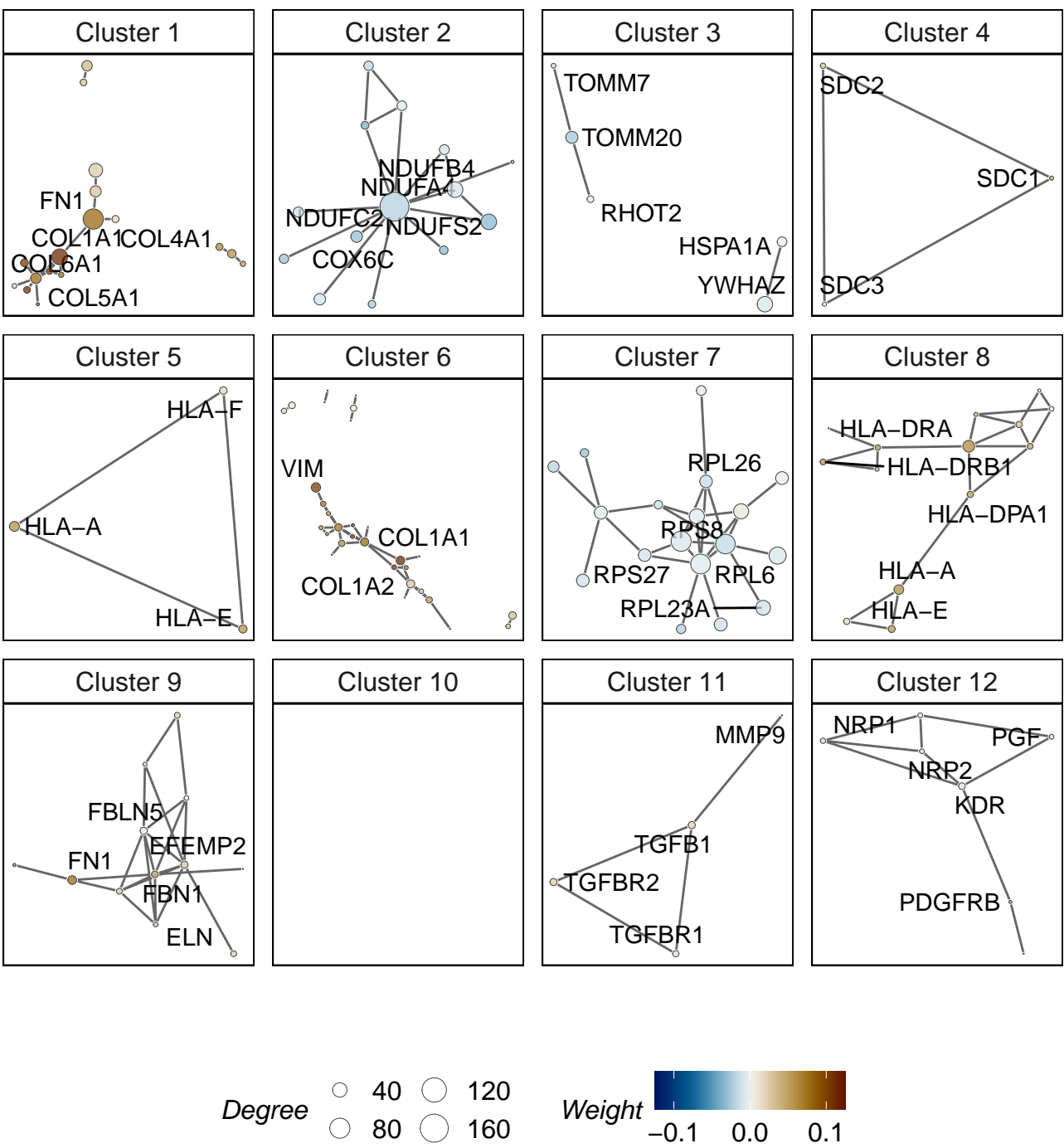

a)

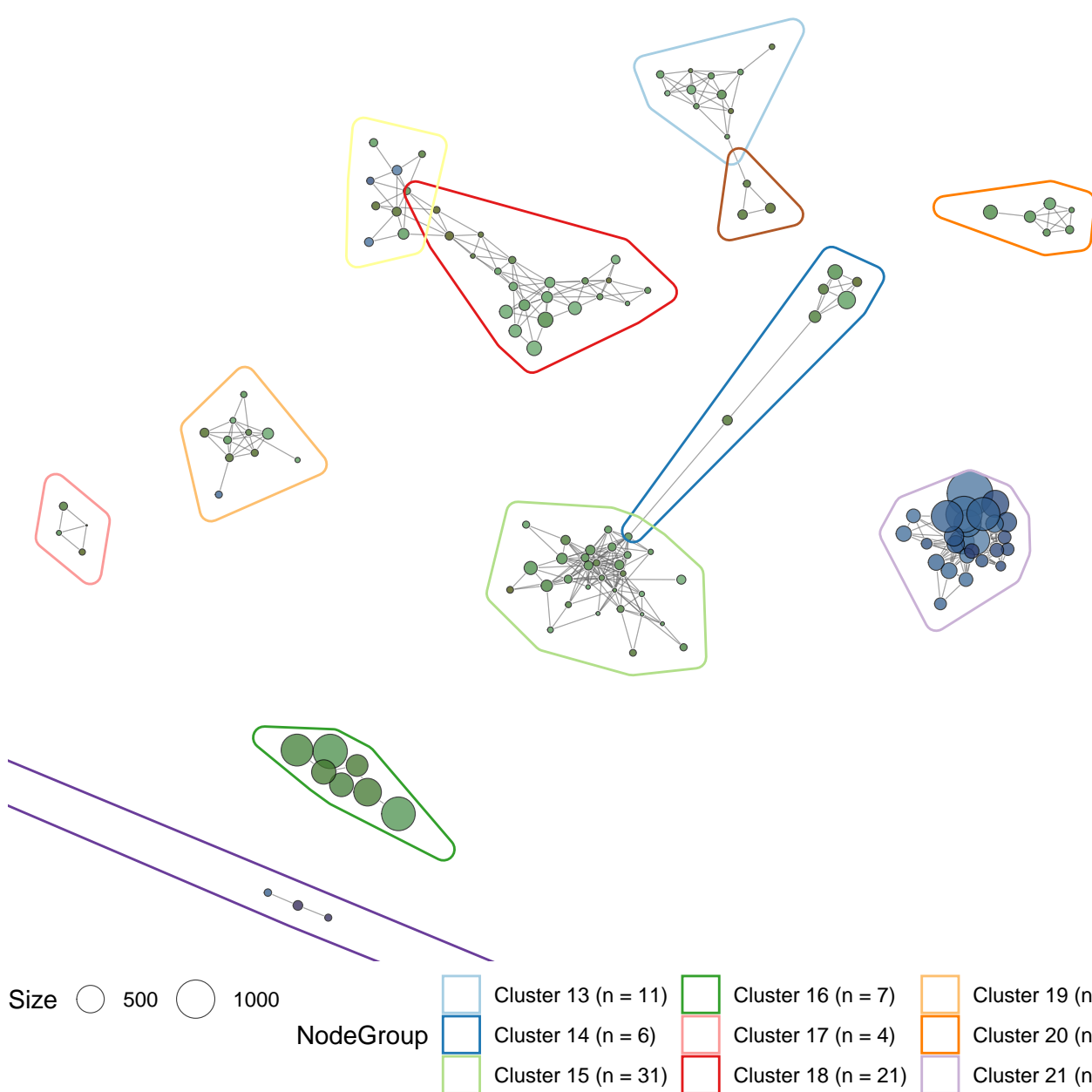

b)

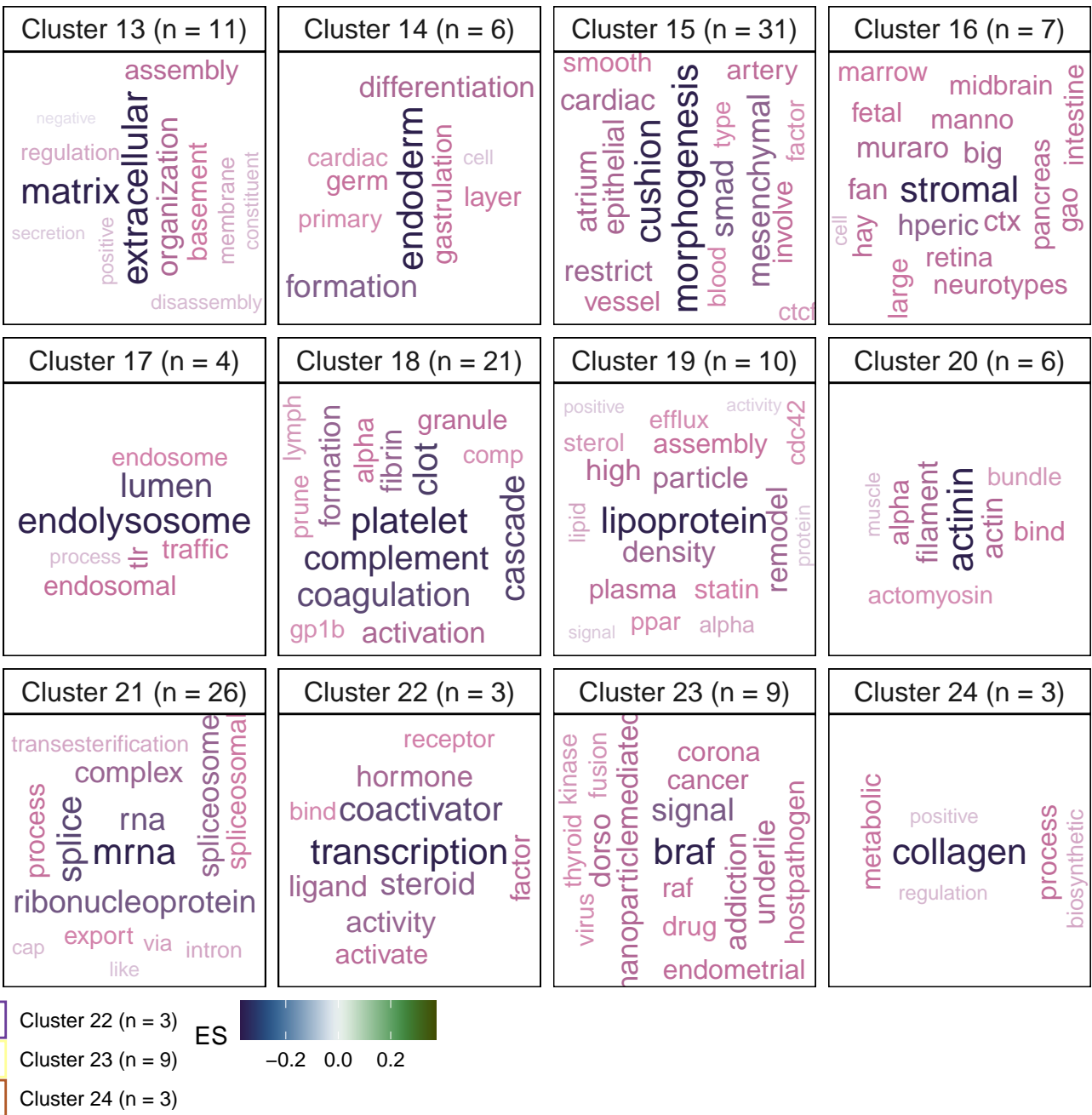

c)

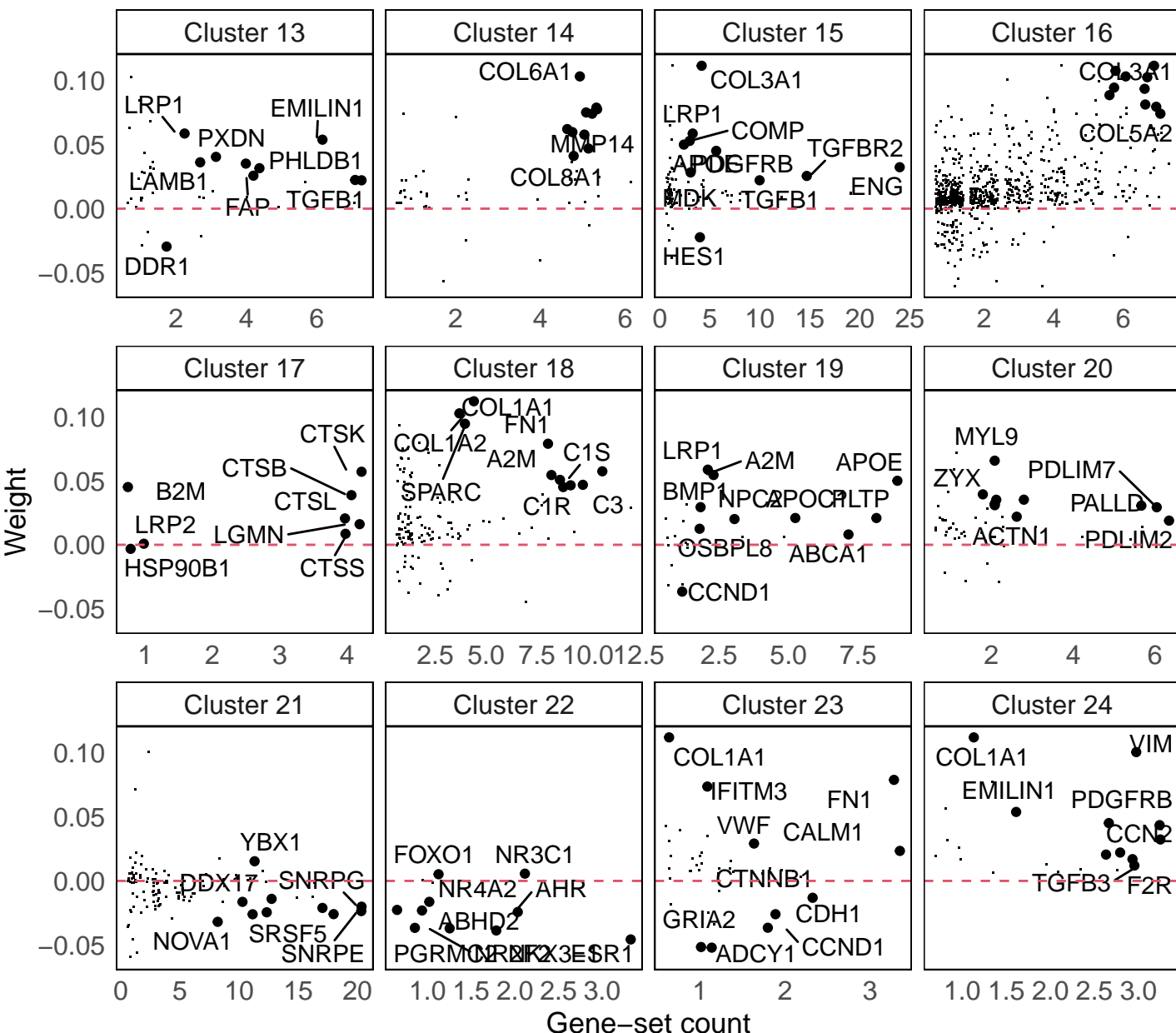

d)

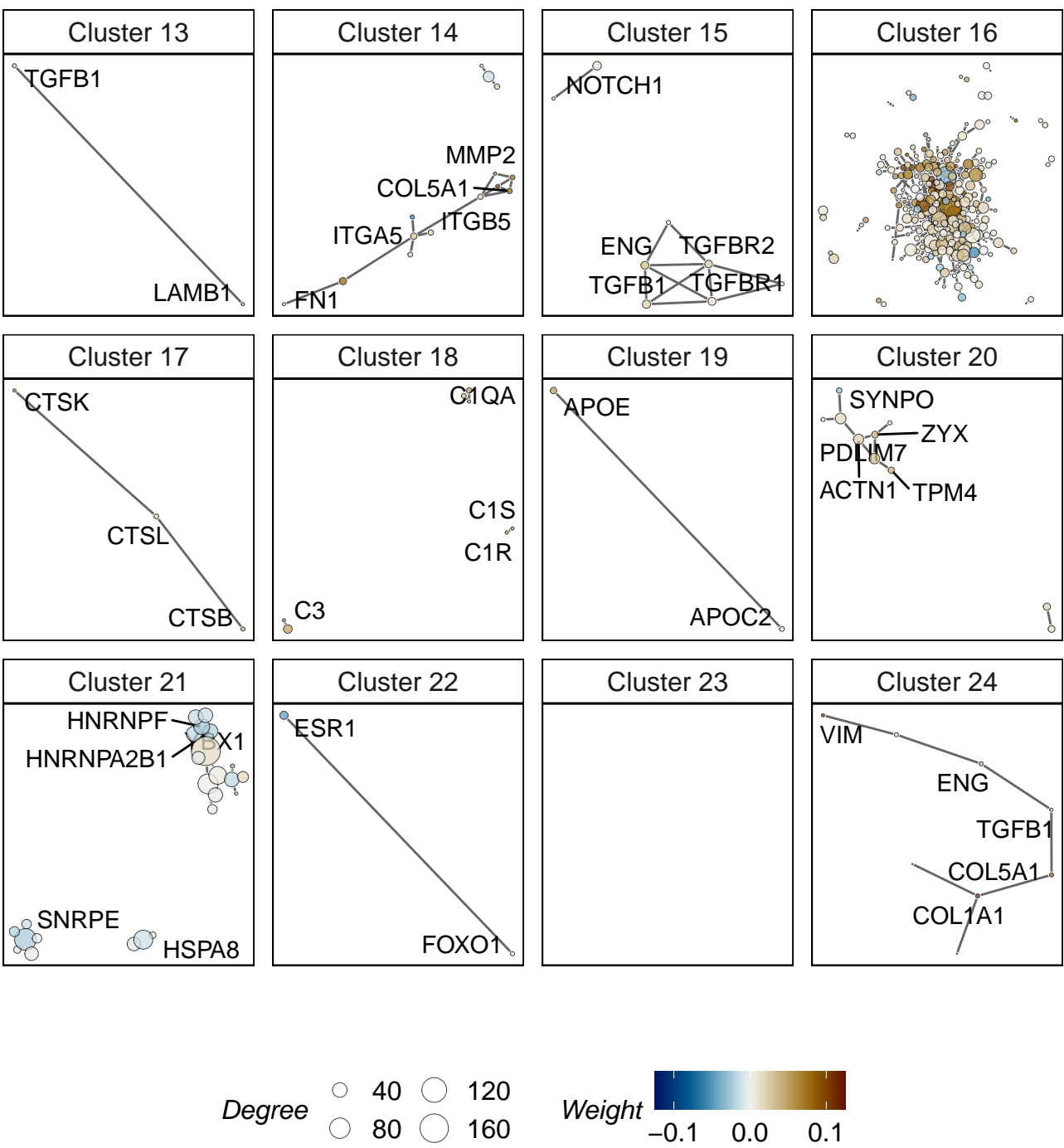

a)

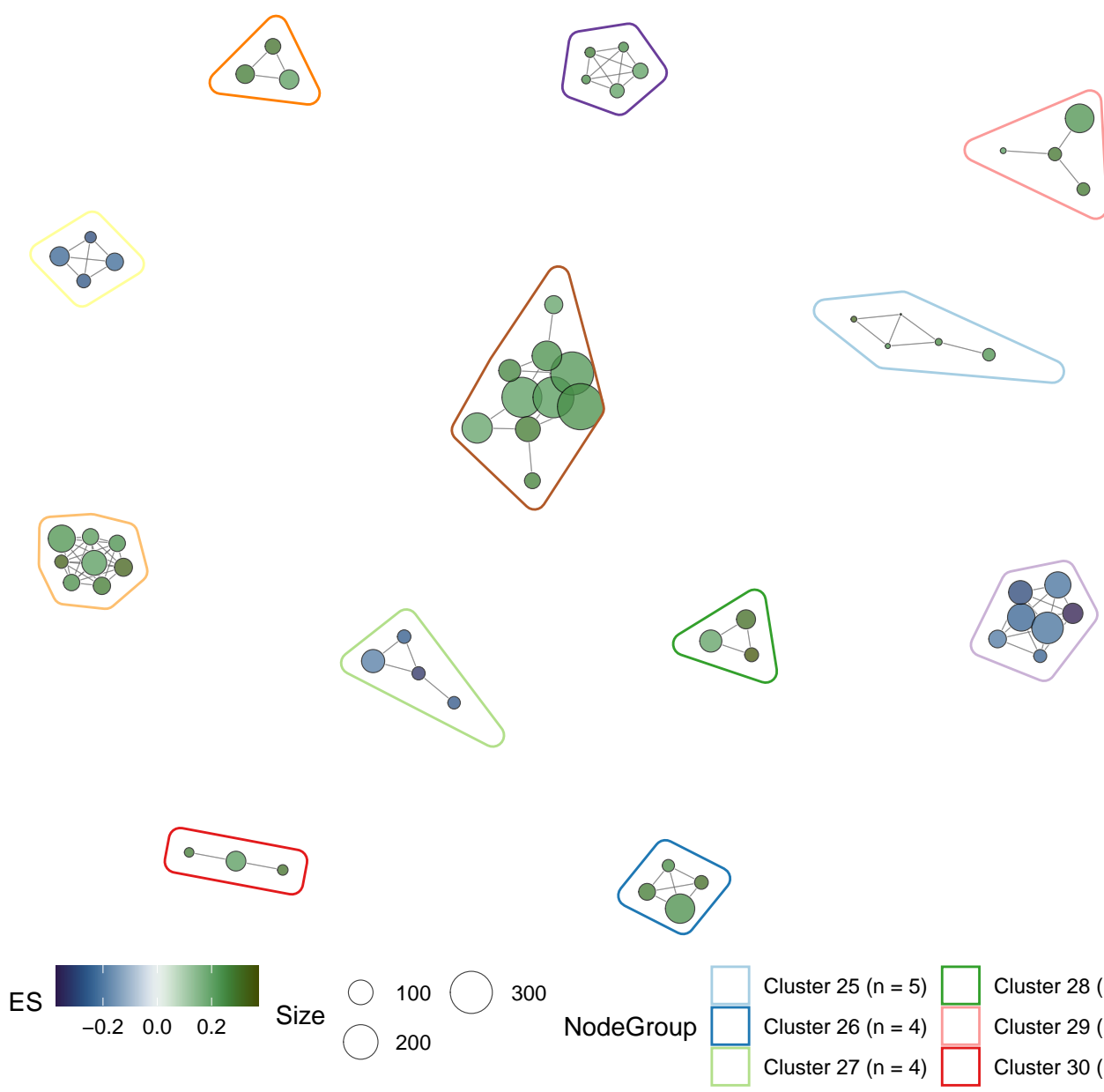

b)

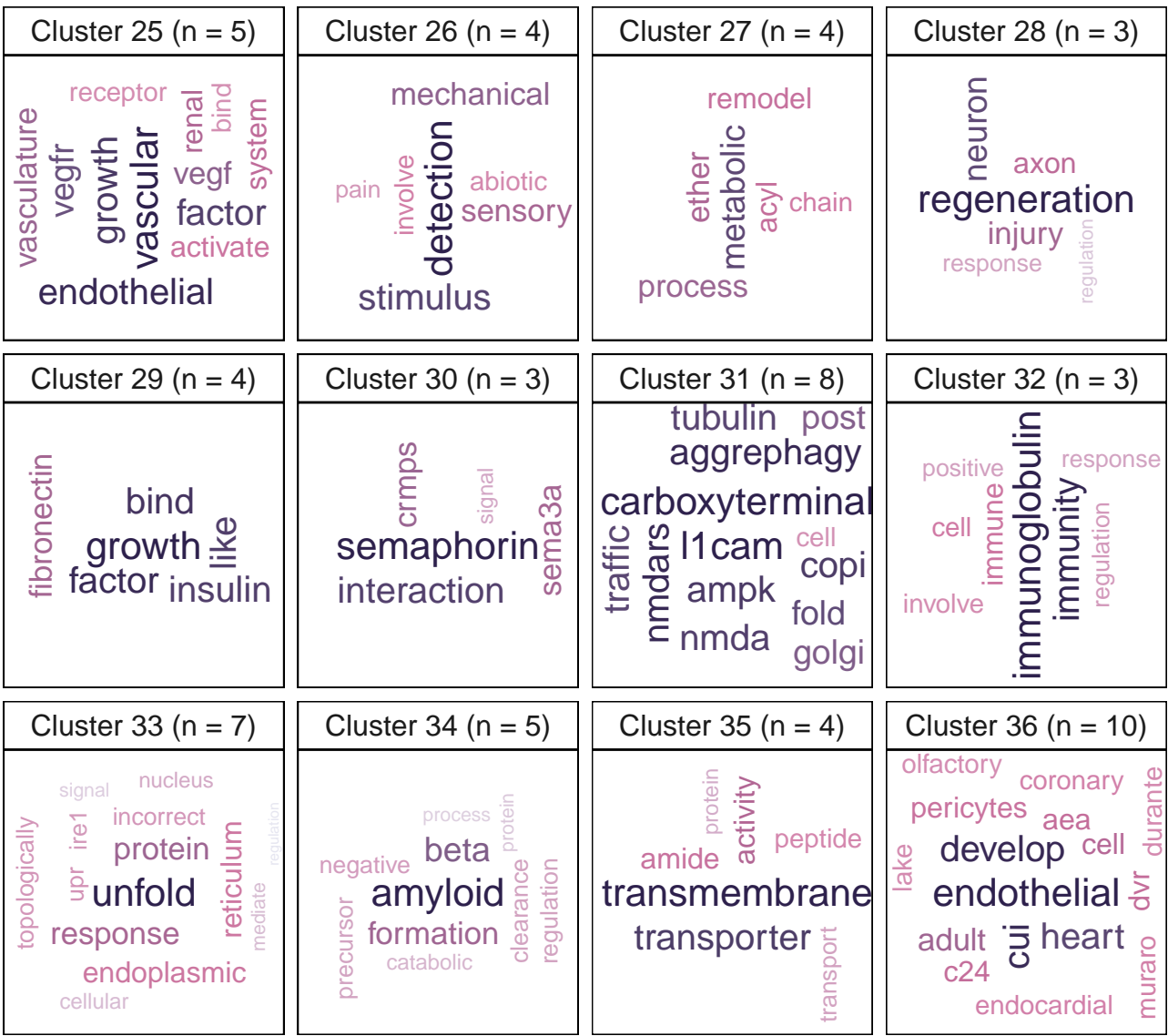

c)

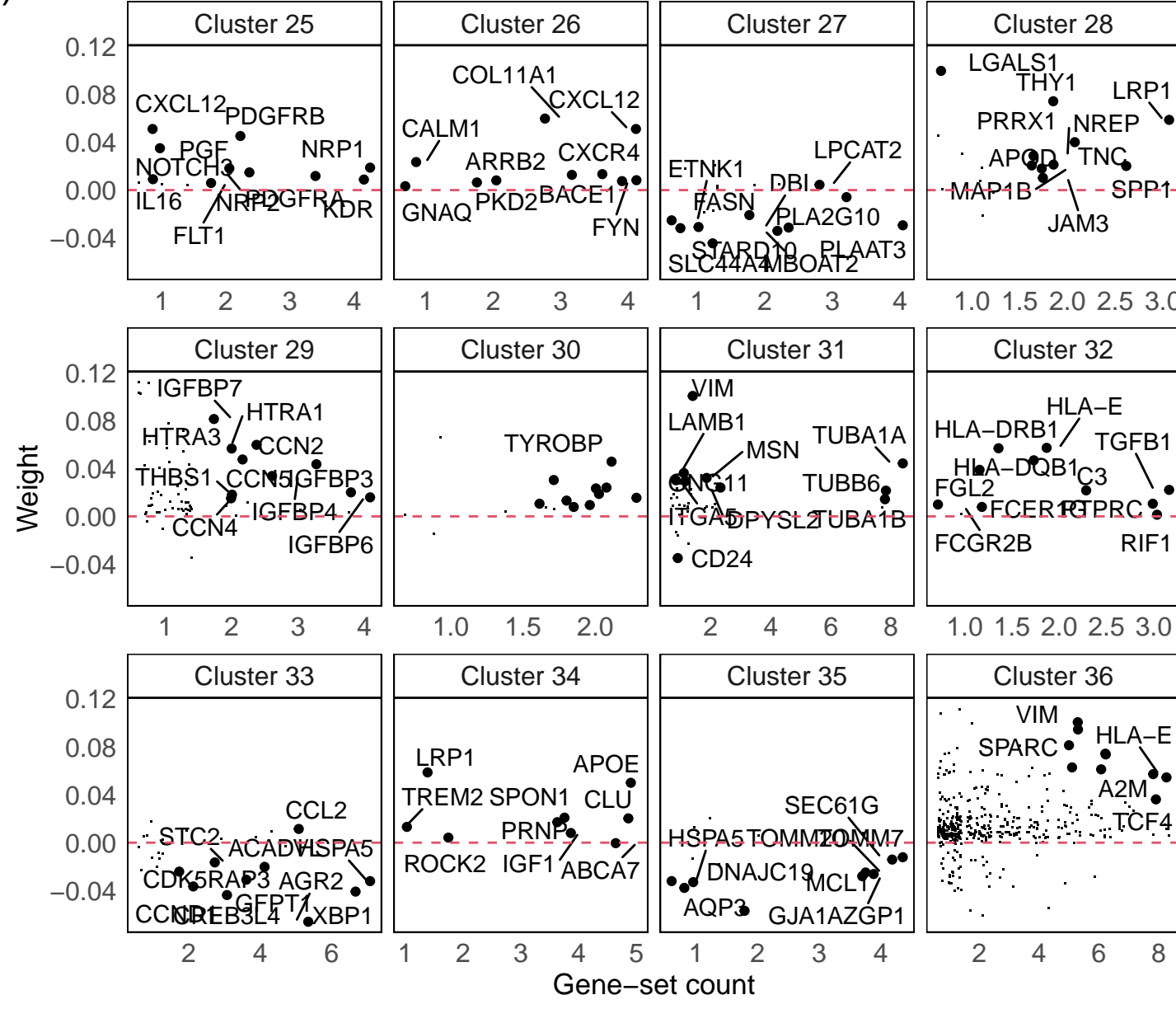

d)

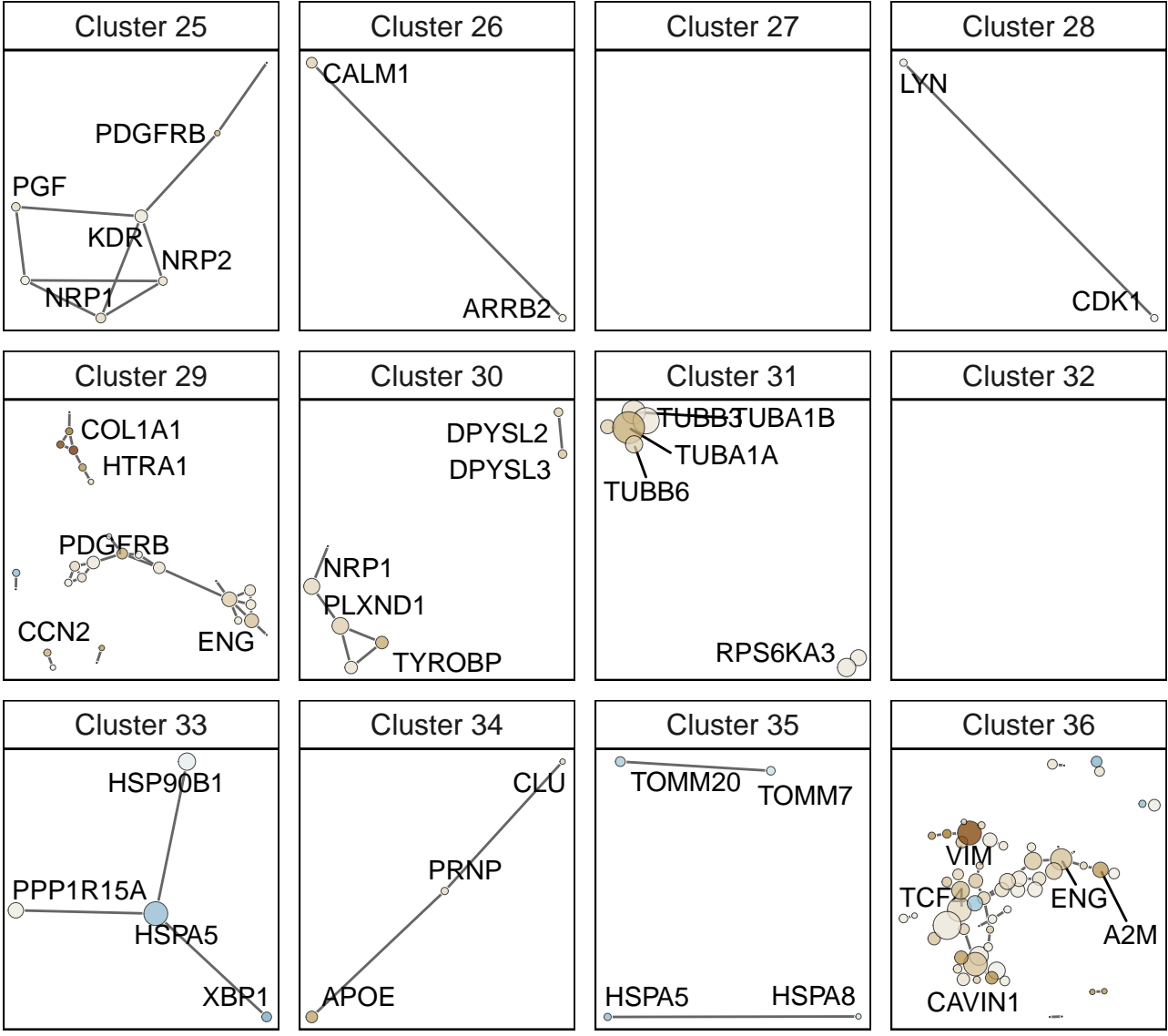

a)

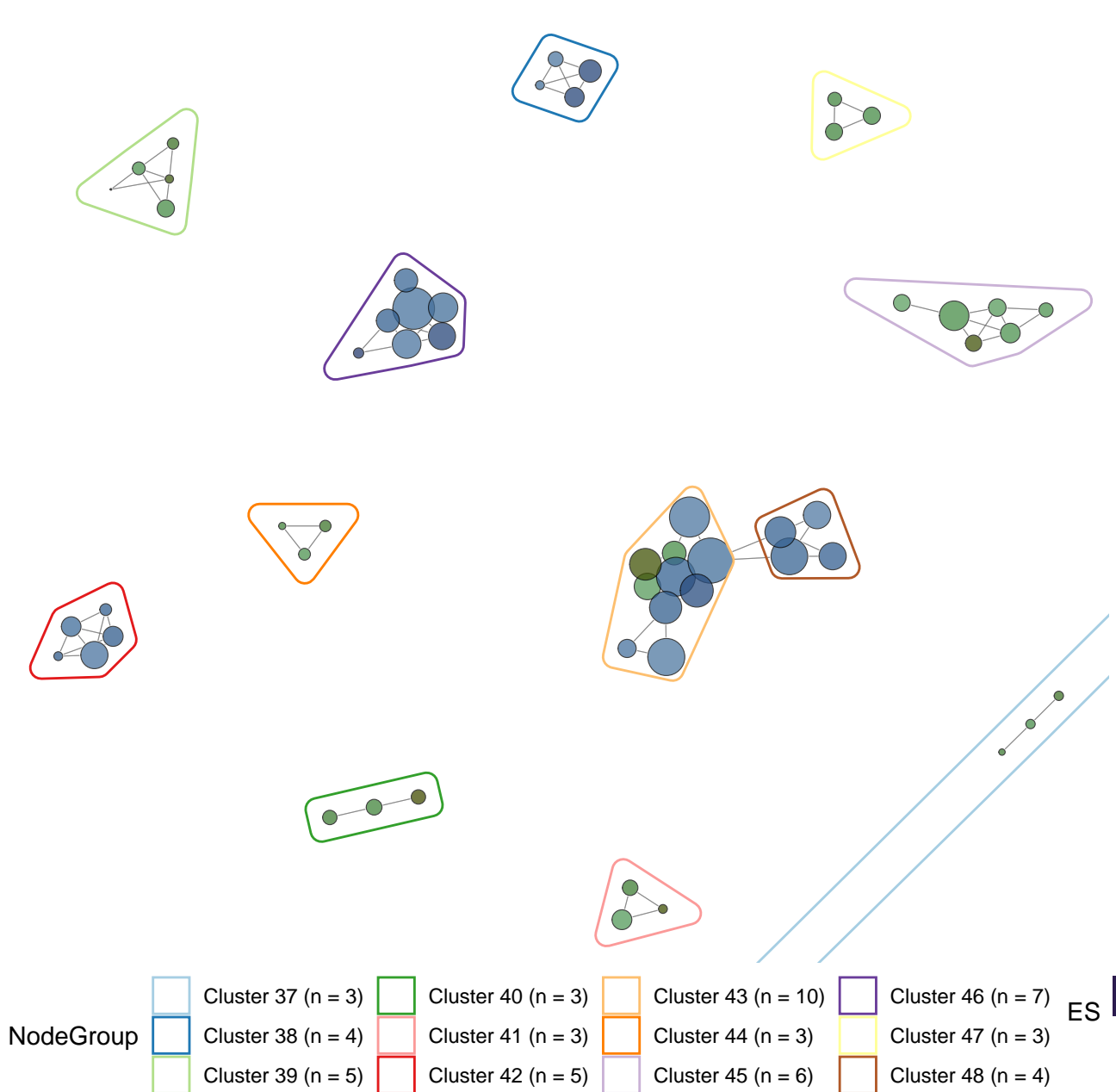

b)

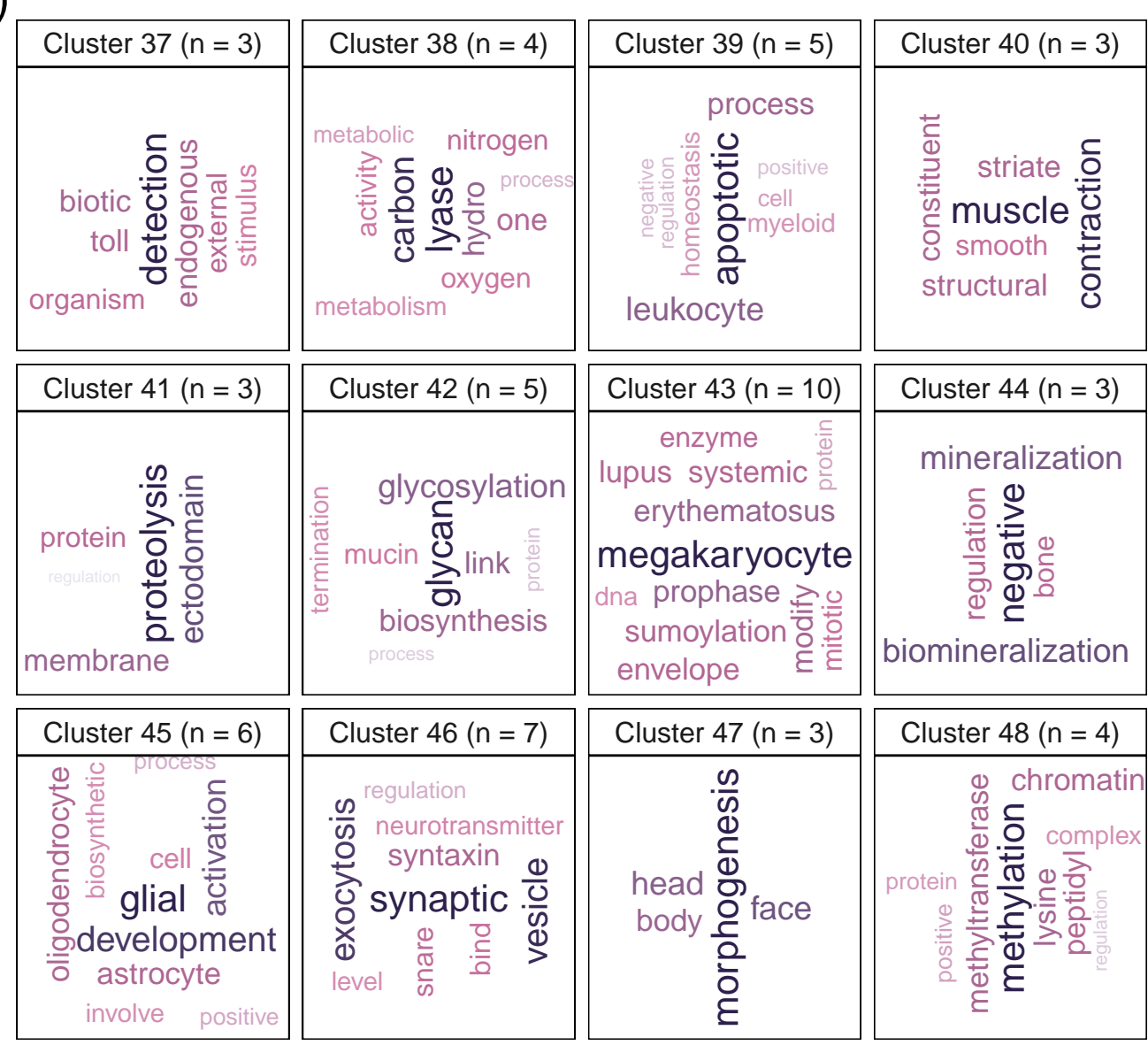

c)

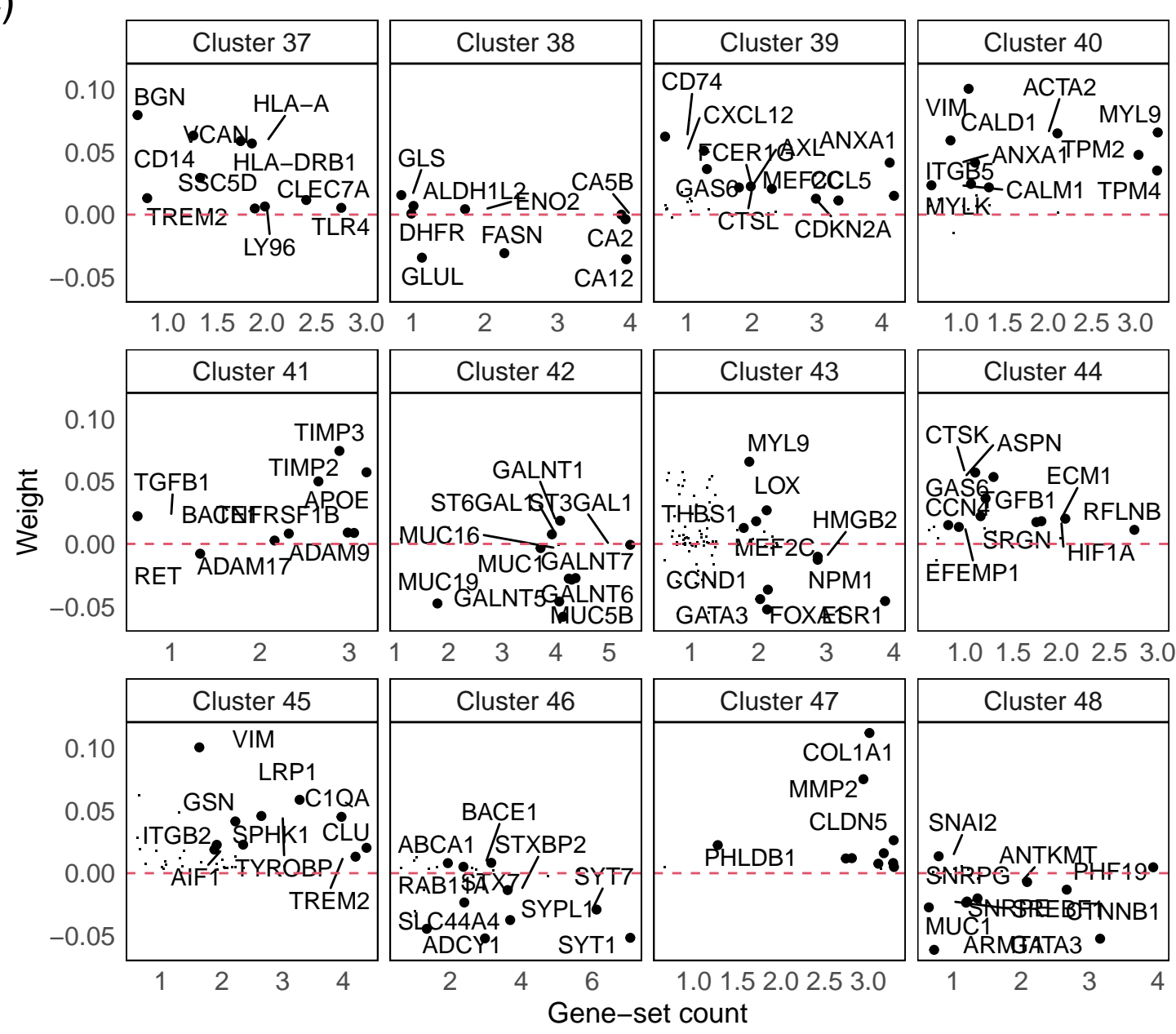

d)

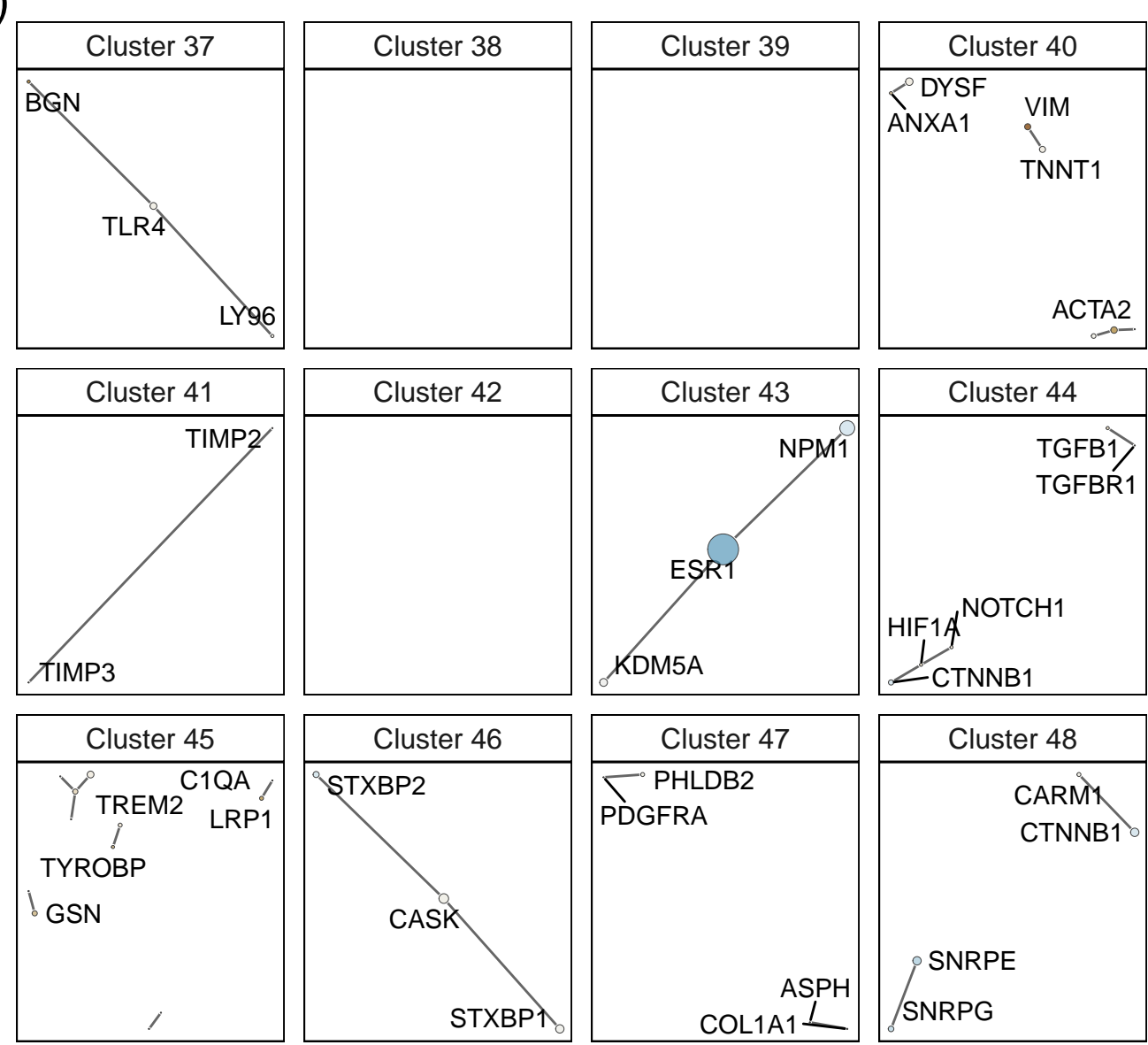

a)

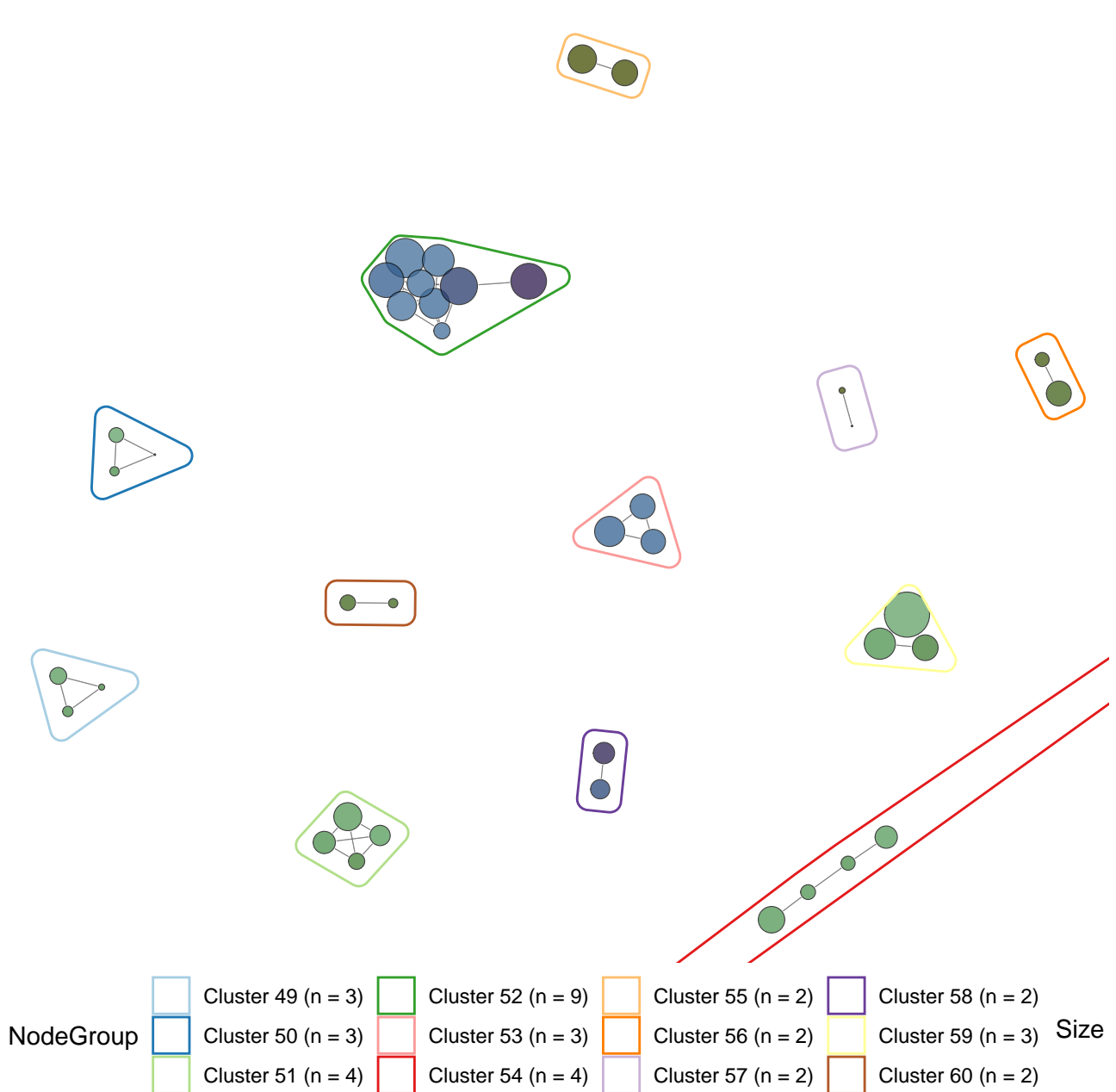

b)

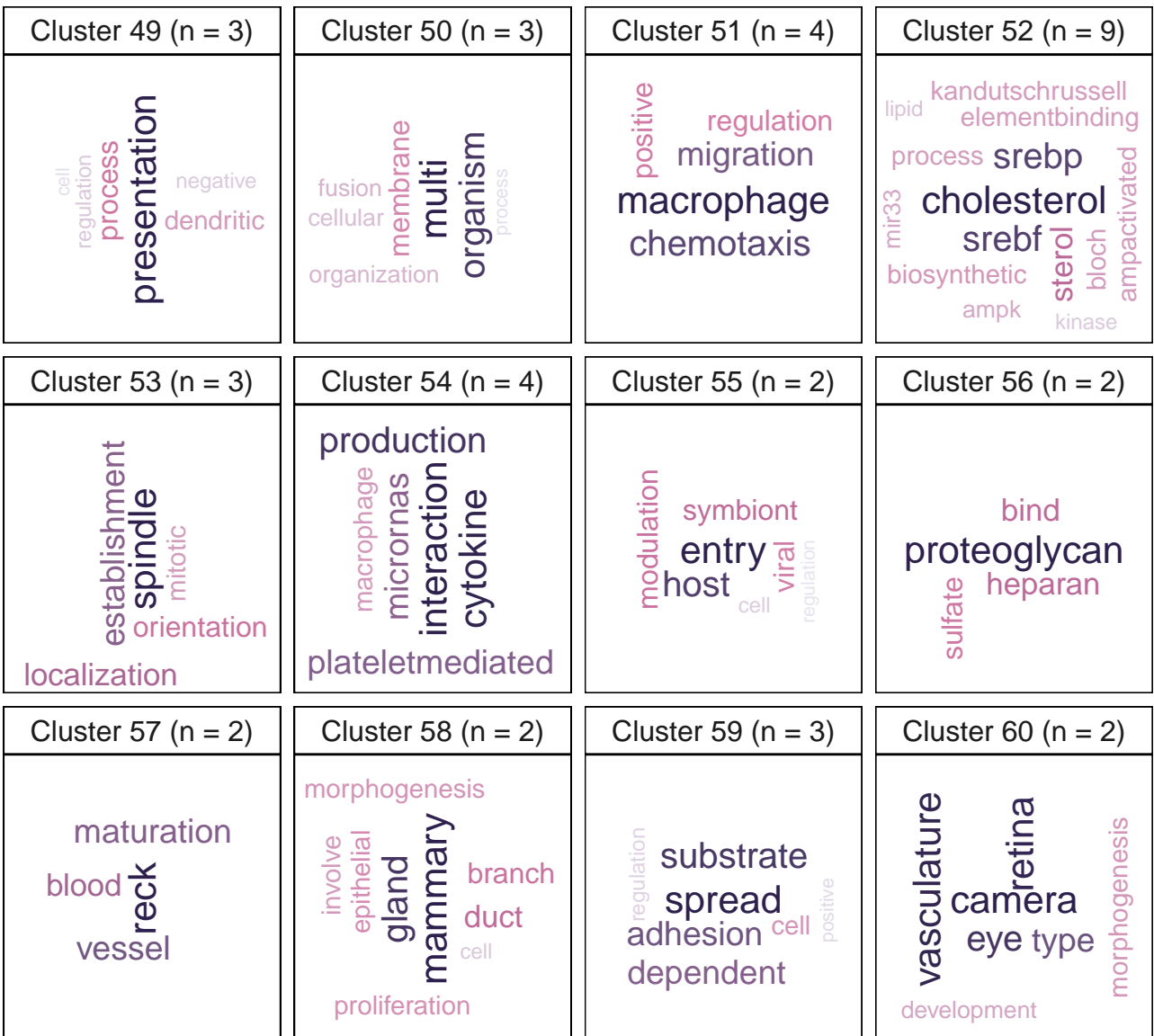

c)

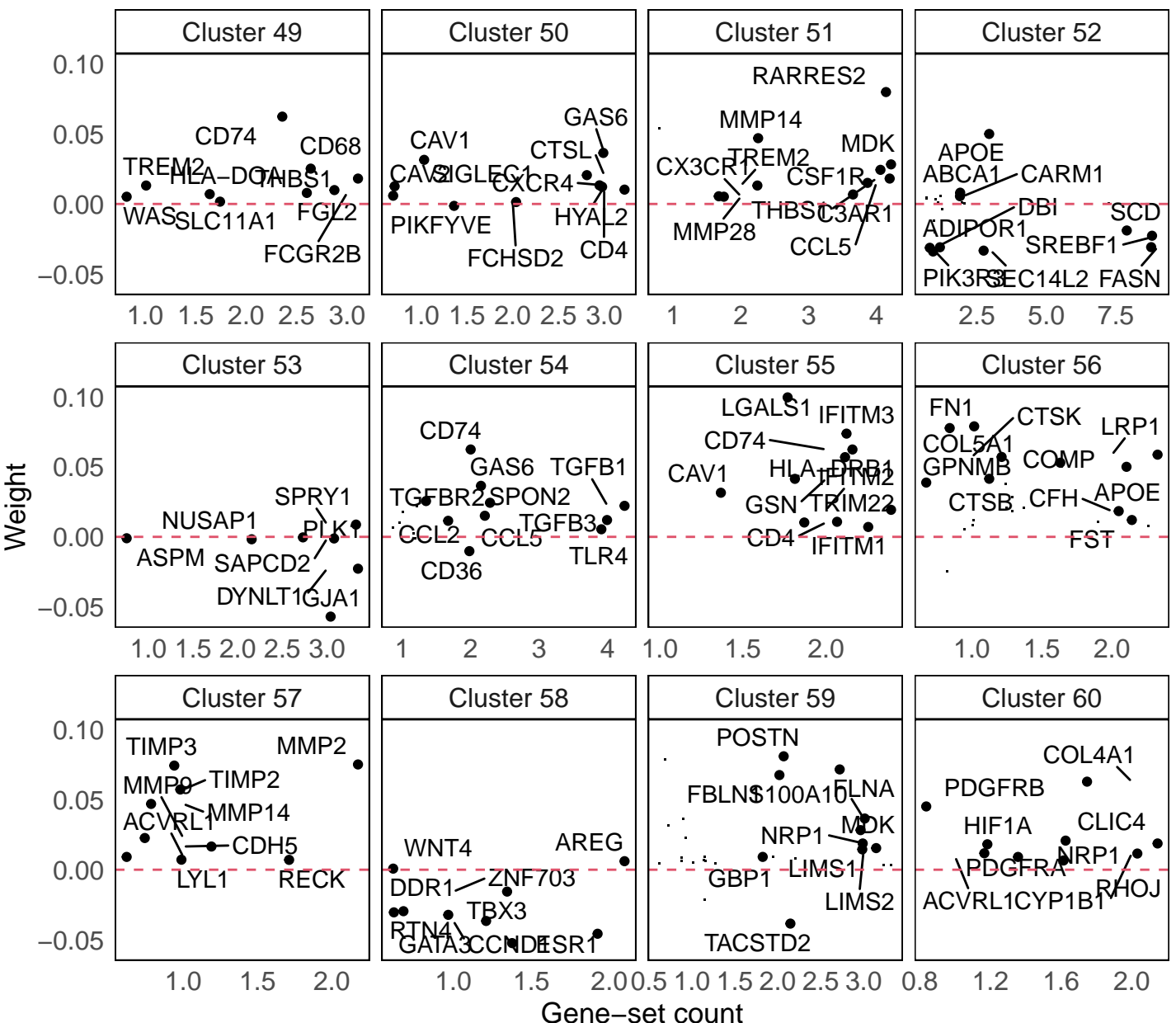

d)

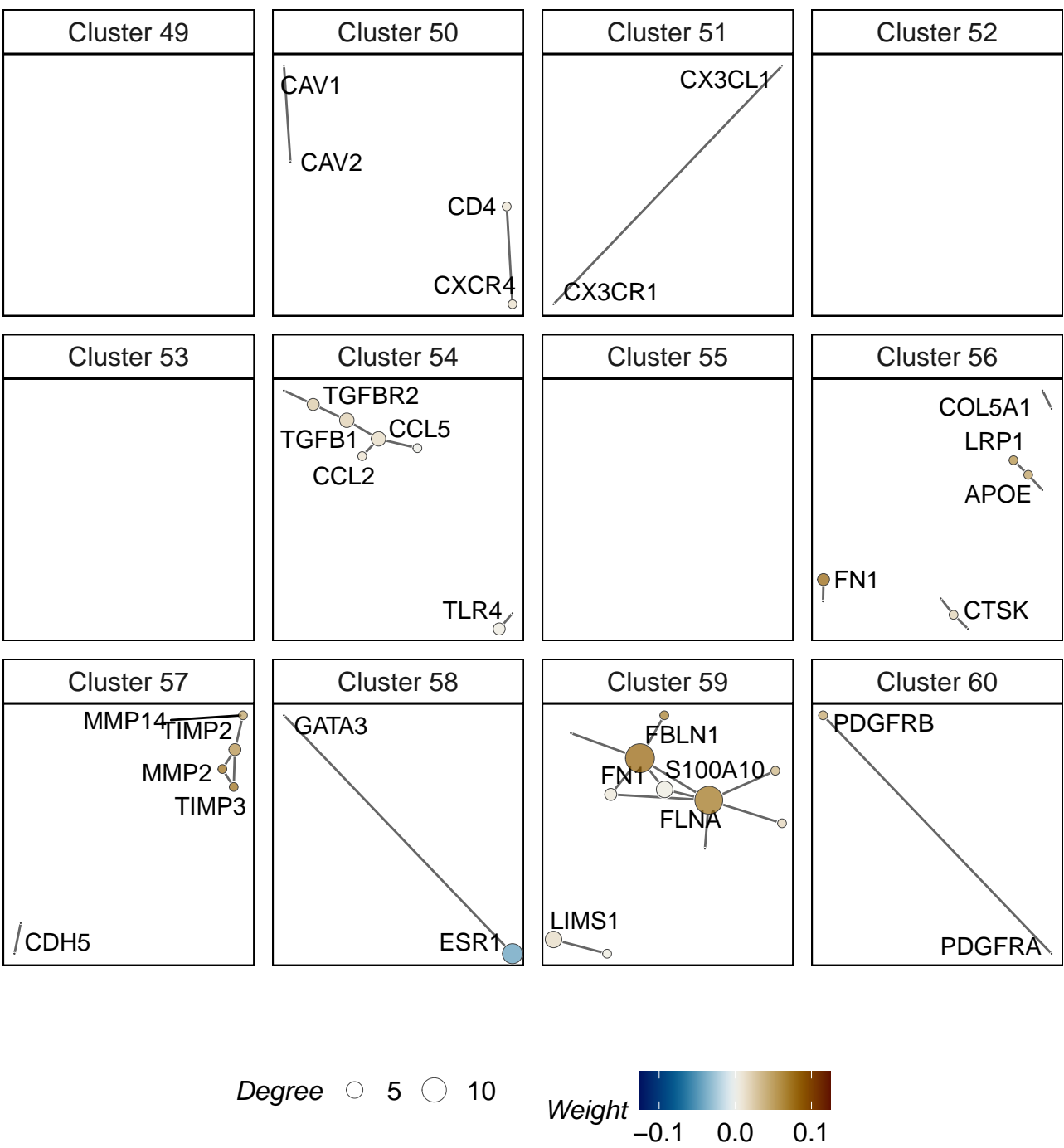

Supplement: Supplementary file 4 — Additional file 4: Top 60 vissE groups identified in the analysis of the spatial RNA-seq dataset. [file 12859_2024_5676_MOESM4_ESM.pdf]
